# Rapid Charge Extraction via Hole and Electron Transfer Layers on Cu_2_O Photocathode for Stable and Efficient Photoelectrochemical Water Reduction

**DOI:** 10.1002/advs.202509030

**Published:** 2025-07-30

**Authors:** Shuangshuang Huai, Xiang Li, Ping Li, Shijian Zhang, Xiuxiu Huang, Wenbin Ruan, Jianli Chen, Zhi Tang, Xiaoli Zhao, Hewen Liu, Xiufang Wang

**Affiliations:** ^1^ Anhui Province Key Laboratory of Advanced Building Materials Anhui Jianzhu University Hefei 230601 China; ^2^ School of Chemistry and Materials Science University of Science and Technology of China Hefei 230026 China; ^3^ State Key Laboratory of Environmental Criteria and Risk Assessment Chinese Research Academy of Environmental Sciences Beijing 100085 China

**Keywords:** Cu_2_O, HER, hole and electron transfer layer, photoelectrochemical water reduction, sandwich structure

## Abstract

Photoelectrochemical (PEC) water reduction offers a promising method for generating “green” hydrogen. The hydrogen evolution reaction (HER) at the photocathode is significantly constrained, primarily because of the rapid recombination of photogenerated electron–hole pairs and the high energy barrier encountered during the water splitting step. Here, a unique “sandwich” structure FeOOH/Cu_2_O/ZnO composite photocathode is fabricated by hydrothermal and electrodeposition methods. Photogenerated holes are extracted and transferred from the Cu_2_O to FTO substrates more easily via the introduction of FeOOH as a hole storage/transport layer. Charge recombination is hindered by the ZnO layer, which functions an electron transfer agent. Hence, the FeOOH/Cu_2_O/ZnO photocathode presents remarkable PEC water reduction capability. The maximum photocurrent density of the FeOOH/Cu_2_O/ZnO photocathode (−2.54 mA·cm^−2^) is 12.7 times greater than that of pristine Cu_2_O (−0.2 mA·cm^−2^) at 0 V_RHE_. The IPCE of FeOOH/Cu_2_O/ZnO reaches 33.7% (455 nm), which is 8.1 times higher than the value of bare Cu_2_O (4.18%). The theoretical calculations reveal that energy barrier of HER on FeOOH/Cu_2_O/ZnO photocathode is dramatically reduced, greatly improving the catalytic activity for HER. This study highlights the crucial functions of solar PEC conversion and offers comprehensive insights into interfacial charge transfer in designing efficient photocathode materials.

## Graphical abstract

A simple strategy is developed to construct a unique “sandwich” structure of FeOOH/Cu_2_O/ZnO composite photocathode. FeOOH as HTL and ZnO as ETL promote the separation of photon‐generated carriers, reduce reaction energy barrier, and enhance the HER kinetic. Therefore, the FeOOH/Cu_2_O/ZnO photocathode material shows excellent performance of PEC water reduction.

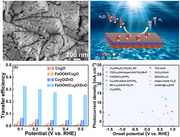

## 1 Introduction

The widespread utilization of H_2_ as a clean fuel plays a pivotal role in transforming the existing energy system that relies on fossil fuels. Hydrogen is also a crucial chemical feedstock that is widely used in the manufacturing of core chemicals, ammonia synthesis, and petroleum refining.^[^ ^1^, ^2^, ^3^ ^]^ PEC water splitting, which combines solar energy collection and water splitting into a single device, is seen to be one of the most promising H_2_ generation technologies in a number of sustainable hydrogen production approaches.^[^ ^4^, ^5^, ^6^ ^]^ The PEC device with p‐type semiconductor as the photocathode (with photogenerated electrons as carriers) directly generates hydrogen on the surface of the photoelectrode by converting light energy into chemical energy and storing it within the molecular bonds of hydrogen. Presently, the reduction of PEC water involves utilizing inorganic p‐type semiconductors, including silicon,^[^ ^7^, ^8^, ^9^ ^]^ metal oxides,^[^ ^10^, ^11^ ^]^ metal phosphides,^[^ ^12^, ^13^ ^]^ and metal sulfides.^[^ ^14^, ^15^ ^]^

Cuprous oxide (Cu_2_O), a naturally occurring p‐type semiconductor, possesses a direct band gap (2 ≈ 2.2 eV). It features suitable conduction and valence band energies, a high theoretical photocurrent density (−14.7 mA·cm^−2^), and an impressive photoconversion efficiency (18%). Additionally, it is abundant in nature and inexpensive. All of these advantages position it as a most promising candidate for photocathode in PEC H_2_ generation. However, the inherent disadvantages of Cu_2_O photocathodes, such as self‐photocorrosion and rapid surface electron–hole recombination, which causes serious stability and inefficiency problems, continue to hinder their practical application for PEC water reduction. In addition, the HER of Cu_2_O photocathode is significantly slower than that of other photocathodes because of a high energy barrier in the water dissociation step (*H*_2_ *O* + *e* ^−^ → H* + *OH* ^−^).^[^ ^16^ ^]^ For overcoming the aforementioned constraints, various surface passivation and engineering approaches have been developed. For example, Luo et al. introduced dual buffer layers to improve the band alignment and photovoltage of photoelectrode for efficient solar power water decomposition.^[^ ^17^ ^]^ Luo et al. designed polycrystalline Cu_2_O photocathode with (111) terminating facets and extraordinarily pure (111) orientation, and it delivered 7 mA·cm^−2^ current density at 0.5  V versus RHE.^[^ ^18^ ^]^ Ren et al. fabricated a Cu_2_O/Ga_2_O_3_/TiO_2_ photocathode modified with Sn/SnO_x_ through simple method. The barrier‐free ohmic contact facilitated electron transfer between the Sn/SnO_x_ and the Cu_2_O/Ga_2_O_3_/TiO_2_ device, achieving a remarkable half‐cell solar‐to‐fuel conversion efficiency.^[^ ^19^ ^]^ Nonetheless, there is a significant difference between the actual performance of Cu_2_O‐based photocathodes and the maximum photocurrent density that can be realized theoretically. Consequently, identifying optimal protective and catalytic overlayers on Cu_2_O photocathodes for efficient PEC water decomposition to produce hydrogen is still a big challenge.

In light of this, it has been established that using engineering interfaces with hole transport layer (HTL) is an effective measure to further promote the separation and transfer of charge. Incorporating HTL within photoelectrode systems plays a critical role in regulating charge transfer dynamics while suppressing detrimental charge recombination processes, thereby enhancing PEC conversion efficiency through optimized interfacial charge management. Thus, many organic and inorganic materials, such as polyaniline,^[^ ^20^ ^]^ pyrrole derivation,^[^ ^21^ ^]^ Co_3_O_4_,^[^ ^22^ ^]^ and FeOOH^[^ ^23^ ^]^ have been taken as HTLs to promote PEC performance. Among them, FeOOH exhibits optimal work function characteristics, inherent stability, and favorable band alignment properties, making it extensively employed in PEC systems. Comprehensive investigations demonstrate that the material's superior PEC performance stems from its nanoscale crystalline framework, which enhances charge carrier mobility and trapping efficiency during water splitting processes. This structural advantage particularly improves hole transfer kinetics at the semiconductor‐electrolyte interface, which is crucial for sustaining catalytic activity. For instance, Xiao et al. introduced FeOOH as a hole transfer layer on Ti‐Fe_2_O_3_, induced a significant band bending effect, and significantly promoted the photocurrent density of NiFeB hydroxide/FeOOH/Ti‐Fe_2_O_3_ photoanodes to 3.29 mA·cm^−2^ at 1.23 V_RHE_.^[^ ^24^ ^]^ Jian et al. demonstrated a strategy of interfered hydrolysis to deposit the mixed‐phase FeOOH(α+β) cocatalysts on BiVO_4_ photoanode. FeOOH (α+β) caused lattice distortion and produced a large number of oxygen vacancies, effectively promoting hole transport.^[^ ^25^ ^]^ Therefore, designing and constructing FeOOH/Cu_2_O composite photoelectrode, in which FeOOH serves as a photogenerated hole‐storage layer for transferring the photogenerated holes from Cu_2_O into the FTO, can not only promote the separation and transport of photogenerated carriers and allow more photogenerated holes to participate PEC water splitting, but also enhance the resistance of Cu_2_O to photocorrosion and improve the stability of photocathode.

The integration of electronic transport layer (ETL) components is a key factor affecting operational efficiency, as it simultaneously improves charge transport dynamics and modifies the microstructure of the active material. Besides, the application of an ETL enhances charge carrier management through ohmic contact formation while providing environmental protection against atmospheric moisture and oxygen. The ETL materials are generally organic molecules^[^ ^26^, ^27^, ^28^ ^]^ and inorganic interface materials (ZnO, MoS_2_, and SnO_2_).^[^ ^29^, ^30^, ^31^ ^]^ Among these electron transport materials, ZnO is a competitive candidate for photoelectrode on account of its excellent electronic and photonic properties. Moreover, because of its high electron mobility, matching work function, appropriate energy level for electron injection and high transparency, ZnO has been employed extensively as an effective ETL material. For example, Yu et al. used ZnO nanoparticles/polystyrene as ETL in the preparation of polymer‐based solar cells to obtain high current density and excellent environmental stability.^[^ ^32^ ^]^ Huang et al. developed a 1.2‐micrometer‐thick inverted ultrathin flexible organic solar cell with In‐doped ZnO as ETL, and achieved the efficiency of 17.0%.^[^ ^33^ ^]^ Meanwhile, ZnO serves as a surface passivation layer (SPL) to eliminate unfavorable surface states while maintaining suitable conduction band alignment and enhanced stability relative to unmodified photoelectrodes. For instance, Tang et al. coated a thin passivation ZnO layer on the WO_3_/BiVO_4_ hybrid nanoplate array through atomic layer deposition process. The WO_3_/BiVO_4_/ZnO photoanode had efficient and stable PEC performance for water splitting.^[^ ^34^ ^]^ Tang et al. constructed an integrated photoanode through using of ZnO and 5,10,15,20‐tetrakis (4‐carboxyphenyl) porphyrin‐cobalt molecules (CoTCPP) on α‐Fe_2_O_3_ photoanodes, and ZnO as the SPL passivated the interfacial states and reduced the level of electron leakage from α‐Fe_2_O_3_ into the electrolyte. The simultaneous incorporation of ZnO and CoTCPP modulated the interfacial charge transfer, boosting a robust PEC reaction.^[^ ^35^ ^]^ Therefore, the modification of ZnO as ETL and SPL on the surface of Cu_2_O photocathode can certainly promote electron transport and enhance the stability of photocathode.

Herein, a sandwich structure FeOOH/Cu_2_O/ZnO photocathode is rationally designed and prepared by simple synthesis method for PEC water splitting. Comprehensive characterizations demonstrate that the FeOOH as an active medium for hole transfer and ZnO as a fast electron transport channel effectively inhibit photocorrosion and photogenerated carrier recombination. The resulting FeOOH/Cu_2_O/ZnO photocathode with excellent long‐term stability exhibits commendable PEC water splitting performance, attaining a current density of 2.54 mA·cm^−2^ at 0 V_RHE_ and boasting an impressively high onset potential (V_on_) of 0.98 V_RHE_. Furthermore, computational analysis through density functional theory (DFT) demonstrates that incorporating FeOOH HTLs and ZnO ETLs facilitates water molecule adsorption and stabilizes HER intermediates, effectively reducing the energy barrier required for water reduction. These results demonstrate that engineering multi‐layer interfaces provides a promising approach for the development of high‐performance PEC materials.

## 2 Results and Discussion

**Figure 1:**
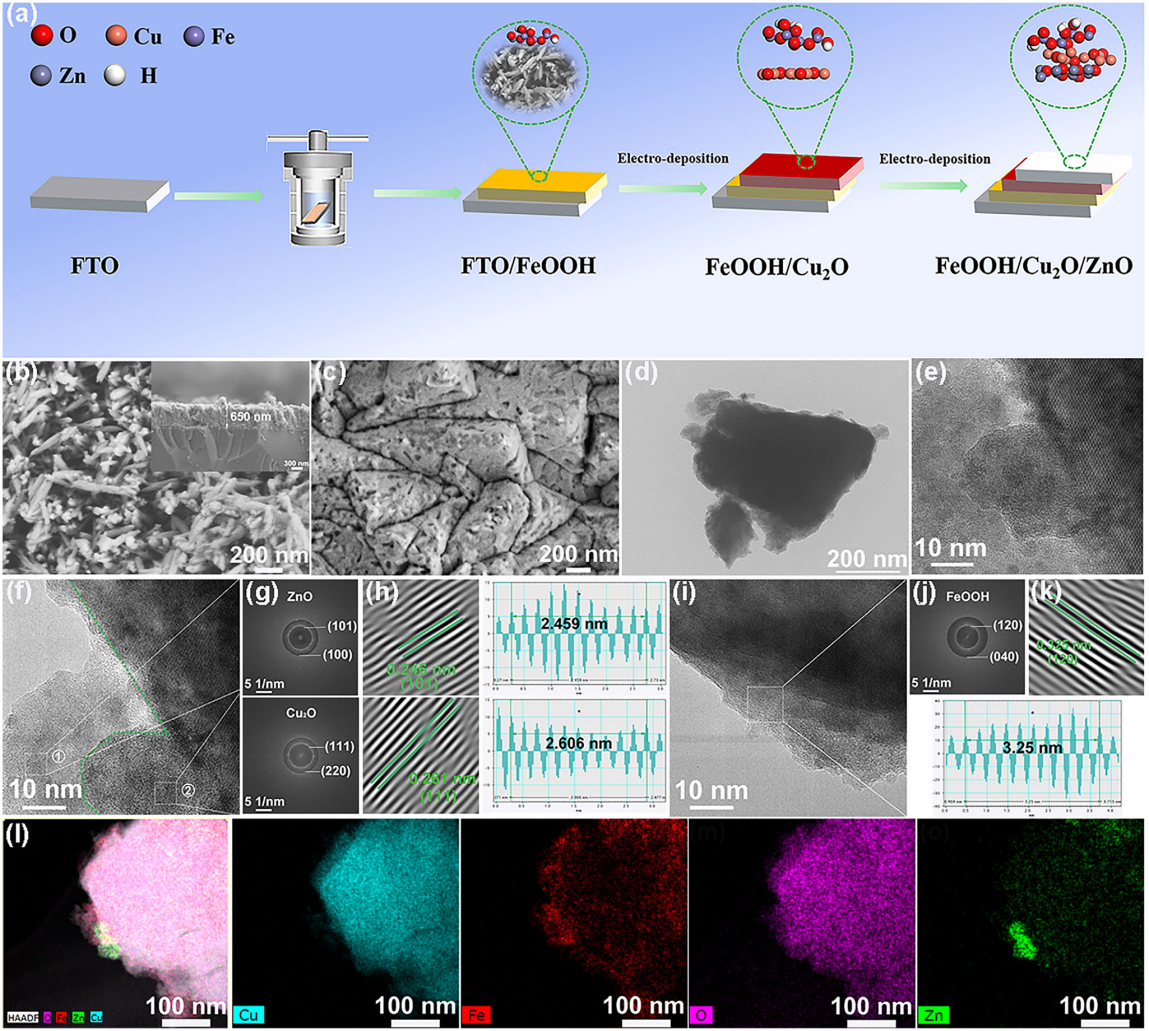
a) Illustration of the synthesis route to the FeOOH/Cu_2_O/ZnO photocathode, top‐view SEM images of b) FeOOH (inset: cross‐sectional SEM image) and c) FeOOH/Cu_2_O/ZnO, d) TEM and e) HRTEM images of FeOOH/Cu_2_O/ZnO, f) enlarged view of the interface between ZnO and Cu_2_O, g) selected‐area FFT patterns and h) inverse FFT patterns and corresponding crystal face spacings of ZnO and Cu_2_O. White square box selected area of dotted lines shows the selected area of electron diffraction (SAED) patterns of ZnO and Cu_2_O. i) Enlarged view of the interface between Cu_2_O and FeOOH, j) selected‐area FFT patterns and k) inverse FFT patterns and corresponding crystal face spacings of FeOOH, and l) EDS elemental distribution images of FeOOH/Cu_2_O/ZnO.

The fabrication procedure for the FeOOH/Cu_2_O/ZnO photocathode is illustrated in **Figure** **1a**, which underwent hydrothermal synthesis and two electrodepositions. The FeOOH exhibits a typical nanorod structure with the diameter of ≈60 nm and length of 200–300 nm. These nanorods interconnect to form ordered arrays, which are uniformly distributed across the FTO substrate (Figure 1b; Figure S1, Supporting Information). Besides, FeOOH nanorod arrays on FTO display an average thickness of 650 nm (the inset in Figure 1b; Figure S2a, Supporting Information). The surface of pure Cu_2_O thin films is composed of dense polyhedral grains hundreds of nanometers in size (Figures S3‐S5, Supporting Information). From the top‐view scanning electron microscopy (SEM) images, the FeOOH/Cu_2_O and Cu_2_O photocathodes exhibit similar morphology and grain size (Figures S4 and S6, Supporting Information). The FeOOH/Cu_2_O photocathode has a thickness of ≈3.5 µm, and the thickness of the Cu_2_O layer on the surface of FeOOH is determined to be ≈2.8 µm (Figures S2b,S7, and S8, Supporting Information), as the cross‐sectional SEM pictures make evident. This result, combined with Figure S9 (Supporting Information), proves that the Cu_2_O film grows on the surface of FeOOH. The morphologies of the Cu_2_O/ZnO and FeOOH/Cu_2_O/ZnO photocathodes display similar triangular planes with a rougher surface as compared to a bare Cu_2_O photocathode in the top‐view SEM images (Figure 1c; Figures S10‐S12b, Supporting Information). The elemental compositions of FeOOH/Cu_2_O/ZnO are detected from energy dispersive X‐ray spectroscopy (EDS) (1l Figure S13, Supporting Information), and the Cu, O, Fe and Zn elements are identified, indicating the coating of ZnO on the surface of FeOOH/Cu_2_O photocathode. The cross‐sectional SEM image clearly shows that the thickness of the FeOOH/Cu_2_O/ZnO photocathode with multilayer structure is ≈4.5 µm and a thin film of ZnO is grown in‐situ on FeOOH/Cu_2_O photocathode, exhibiting a thickness of 1 µm. (Figures S2c and S14, Supporting Information). Moreover, the sectional SEM image with elemental analysis (Figure S15, Supporting Information) for the FeOOH/Cu_2_O/ZnO photocathode can verify that the intended structure is successfully created. The micromorphology is further determined by transmission electron microscopy (TEM) and high resolution TEM (HRTEM). The TEM images indicate that pristine Cu_2_O and FeOOH/Cu_2_O/ZnO have cubic structure (Figure 1d; Figure S16, Supporting Information). The HRTEM images give more detailed details of the existence of Cu_2_O, FeOOH and ZnO in the FeOOH/Cu_2_O/ZnO photocathode (Figure 1e,f,i). Figure 1f is the enlarged view of the interface between ZnO and Cu_2_O in the FeOOH/Cu_2_O/ZnO photocathode, and the corresponding selected FFT‐converted images exhibit the diffraction facets of ZnO and Cu_2_O (Figure 1g). The lattice fringes with interplanar spacings of 2.46 and 2.61 Å are calculated from the noninterface area, which can be attributed to ZnO (101) and Cu_2_O (111) (Figure 1h). Meanwhile, the lattice fringe of 3.25 Å can be distinctly visible in the HRTEM image, which matches the (111) crystal plane of FeOOH (Figure 1i‐k). The consistent distribution of Cu, O, Fe, and Zn on the FeOOH/Cu_2_O/ZnO photocathode is confirmed by the EDS elemental distribution images of FeOOH/Cu_2_O/ZnO (Figure 1l; Figure S17, Supporting Information). These results demonstrate the multilayer FeOOH/Cu_2_O/ZnO photocathode has been successfully prepared.

**Figure 2:**
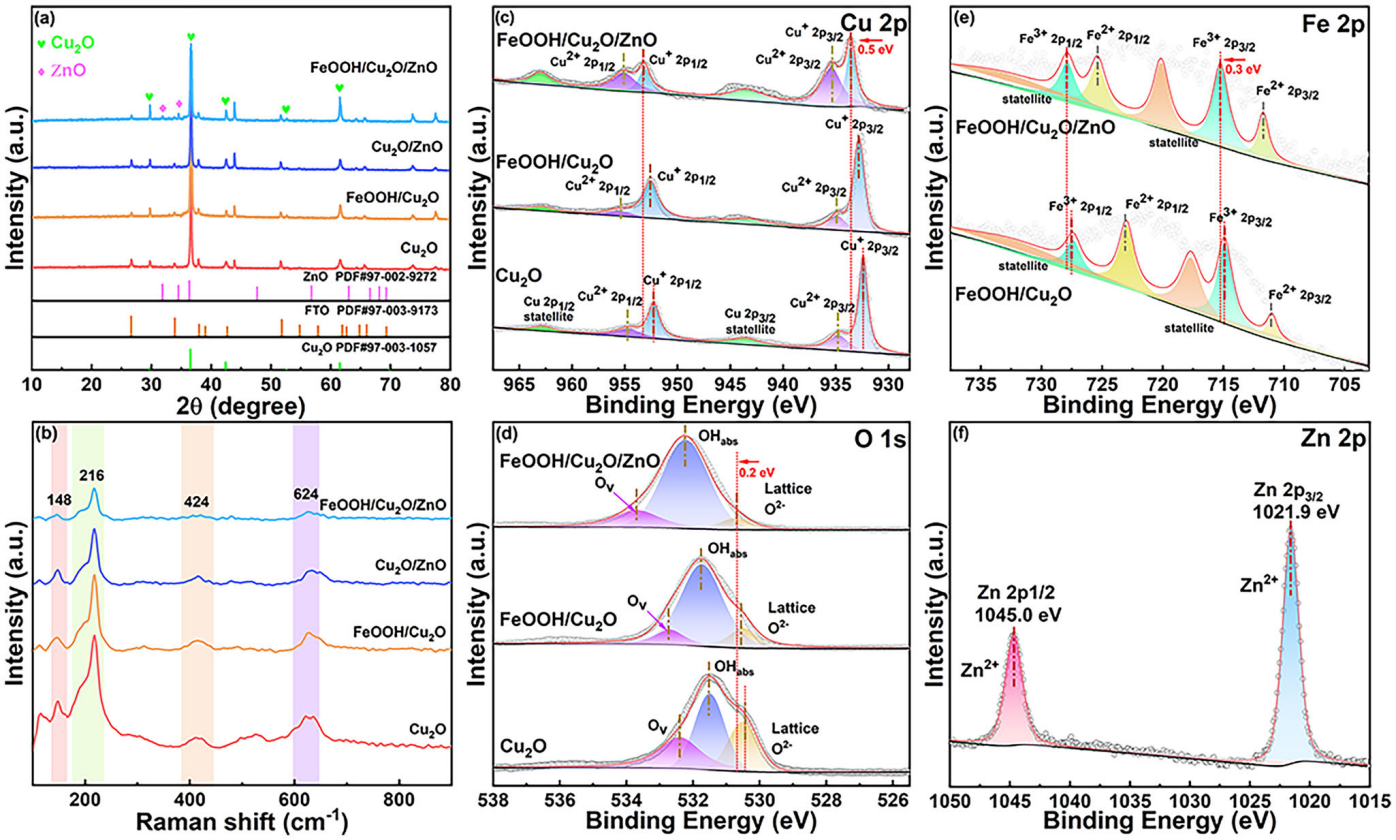
a) XRD patterns, b) Raman spectra of Cu_2_O, FeOOH/Cu_2_O, Cu_2_O/ZnO, and FeOOH/Cu_2_O/ZnO photocathodes, and XPS spectra of Cu_2_O, FeOOH/Cu_2_O and FeOOH/Cu_2_O/ZnO photocathodes: c) Cu 2p, d) O 1s, e) Fe 2p, and f) Zn 2p.

The X‐ray diffraction (XRD) analysis presented in Figure S18a (Supporting Information) indicates that all detected peaks are attributable to FeOOH (PDF#01‐075‐1594), providing clear evidence of the successful growth of crystalline FeOOH on the FTO substrate. From **Figure** **2**a, the XRD patterns indicate that the Cu_2_O has a cubic structure (PDF#97‐003‐1057), which is a polycrystalline thin film with a pronounced [111] preferred orientation. No obvious diffraction peak of FeOOH is detected on the FeOOH/Cu_2_O photocathode, and this may be because the FeOOH layer is thinner and covered by the Cu_2_O layer. The peaks at 31.8° and 36.3° in the FeOOH/Cu_2_O/ZnO are indexed to the characteristic (100) and (101) planes of ZnO (PDF#97‐002‐9272). Raman spectral analysis distinctly displays characteristic crystalline FeOOH peaks in the examined material (Figure S18b, Supporting Information). Figure 2b reveals the Raman spectra of Cu_2_O, FeOOH/Cu_2_O, Cu_2_O/ZnO, and FeOOH/Cu_2_O/ZnO samples. The cubic crystal structure of Cu_2_O, classified under the $O_{h}^{4}$ space group, theoretically permits only the Γ^+^_25_ vibrational mode to exhibit Raman activity among its six fundamental modes.^[^ ^36^ ^]^ Structural imperfections, however, induce symmetry breaking that activates normally forbidden vibrational modes, leading to varied Raman characteristics across different Cu_2_O configurations. The Raman spectral analysis shows the four characteristic peaks at 148, 216, 424, and 624 cm^−1^ (Figure 2b), corresponding to the symmetry Γ^−^_15_, 2Γ^−^_12_, 4Γ^−^_12_, and Γ^−^_12_ + Γ^+^_25_ lattice vibrations of both Cu_2_O and FeOOH/Cu_2_O/ZnO catalysts, respectively.^[^ ^37^ ^]^ The peak shapes of all samples are similar, and the peak positions have not changed obviously, while the intensity of the Raman signals is significantly reduced.

Surface chemical states and composition are further investigated through X‐ray photoelectron spectroscopy (XPS) measurements. The survey spectra further confirm that the composite photocathodes are successfully prepared (Figure S19, Supporting Information). Figure 2c shows detailed Cu 2p spectra. The Cu_2_O photocathode displays primary Cu^+^ signals at 932.4 eV (2p_3/2_) and 952.2 eV (2p_1/2_), accompanied by Cu^2+^ signatures at 934.7 and 954.7 eV respectively. Satellite features observed at 943.7 and 962.8 eV confirm partial surface oxidation to Cu (II) species during fabrication. The FeOOH/Cu_2_O/ZnO composite exhibits analogous Cu^+^ peaks at marginally shifted positions (932.7 and 952.5 eV) and Cu^2+^ components at 934.5 and 954.4 eV. Notably, systematic binding energy upshifts in Cu 2p orbitals relative to bare Cu_2_O and FeOOH/Cu_2_O systems indicate electron transfer toward ZnO layers. The O1s XPS signals of Cu_2_O, FeOOH/Cu_2_O and FeOOH/Cu_2_O/ZnO are respectively shown in Figure 2d. The peaks of O 1s in Cu_2_O at 530.4, 531.50, 532.50 eV indicate the binding energy of lattice O^2−^, surface adsorbed oxygen species (OH_abs_) and oxygen vacancies (O_v_). XPS analysis reveals a 0.2 eV positive shift in lattice oxygen binding energy for the FeOOH/Cu_2_O/ZnO composite, accompanied by significant OOH content elevation and lattice oxygen reduction, indicating enhanced oxidation states through interface engineering. The Fe 2p spectrum (Figure 2e) of FeOOH/Cu_2_O displays characteristic Fe^2+^ signatures at 711.1 and 723.1 eV and Fe^3+^ contributions at 714.9 and 727.5 eV. For FeOOH/Cu_2_O/ZnO, incorporation of ZnO induces 0.3 eV positive shifts in Fe 2p_3/2_ binding energy, 715.2 and 727.8 eV for Fe^3+^; 711.3 and 724.4 eV for Fe^2+^, suggesting improved hole transport properties through valence state modification. These changes also indicate the interaction between the semiconductors.^[^ ^38^ ^]^ Figure 2f indicates that Zinc speciation analysis confirms successful ZnO deposition with Zn^2+^ 2p_3/2_ and 2p_1/2_ peaks observed at 1021.9 and 1045.0 eV respectively. These XPS characterizations indicate the simultaneous presence of Cu^+/2+^ and Fe^2+/3+^ redox pairs in FeOOH/Cu_2_O/ZnO photocathode. This coexistence facilitates the cyclic circulation of photogenerated electrons, thereby promoting the PEC reaction process.

**Figure 3:**
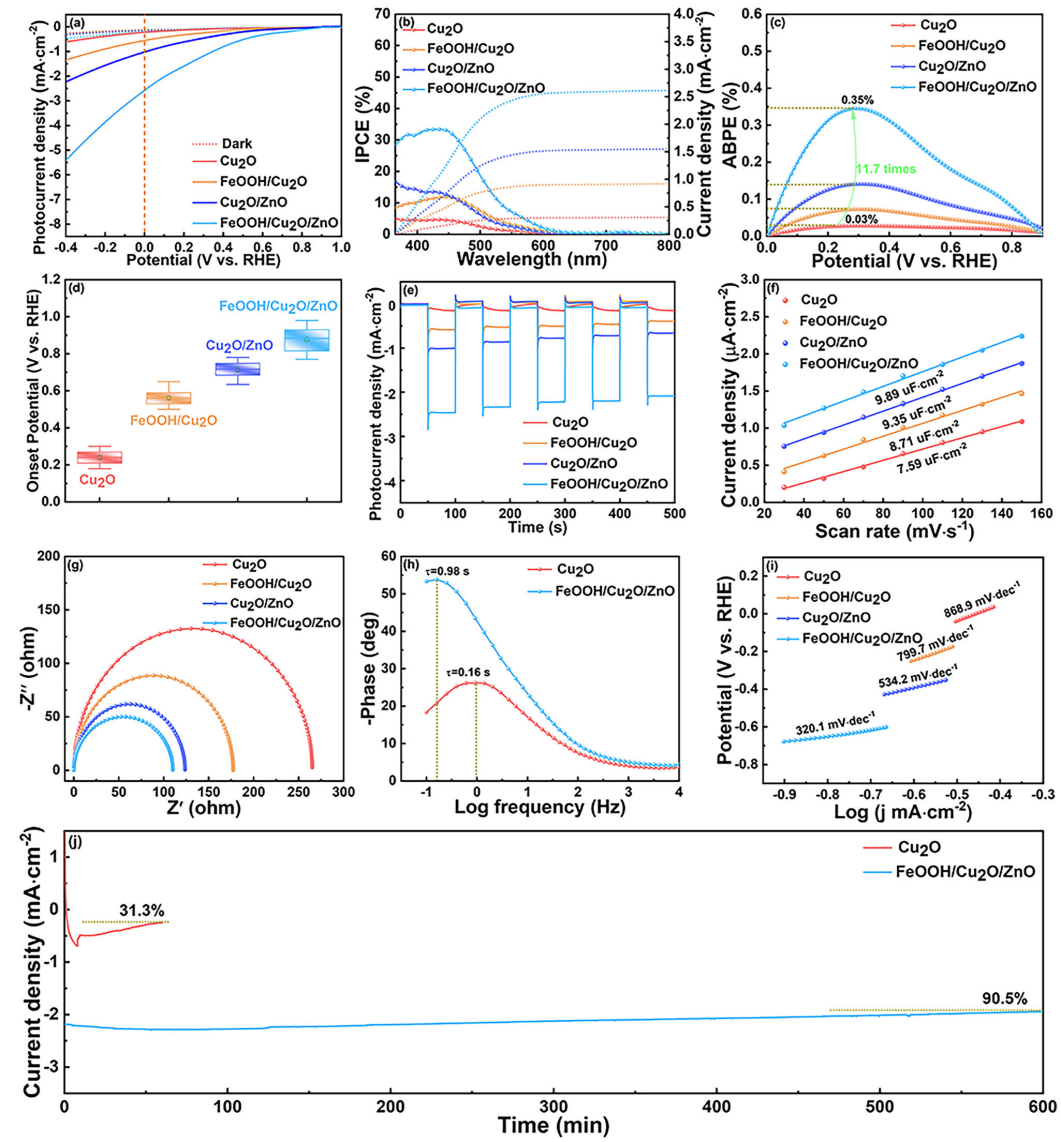
a) LSV plots under dark and light conditions, b) IPCE plots, c) ABPE curves, d) statistical onset potential, e) transient photocurrent responses, f) double layer capacitor (Cdl) values, g) EIS Nyquist plots, h) Bode plots, i) Tafel plots and j) photocurrent stability curves.

The PEC water splitting capacity of photocathodes is evaluated by linear sweep voltammetry (LSV) method. In dark conditions, all of the photocathodes exhibit reduced currents (**Figure** **3**a), indicating that there is no detectable HER activity in the potential range. The outstanding light responsiveness of photocathodes is demonstrated by the quickly increasing current density seen in LSV curves examined under illumination. Additionally, the photocurrent density rises steadily as the bias gets more negative. The optimized FeOOH/Cu_2_O/ZnO photocathode shows a remarkable photocurrent density (−2.54 mA·cm^−2^) at 0 V versus RHE. This value is 12.7 times higher than that of bare Cu_2_O (−0.2 mA·cm^−2^), 4.88 times greater than that of Cu_2_O/ZnO (−1.01 mA·cm^−2^), and 2.54 times superior to that of FeOOH/Cu_2_O (−0.52 mA·cm^−2^). Compared to previously documented Cu_2_O‐based photocathodes, this one produces a comparatively greater photocurrent density (Figure S20 and Table S1, Supporting Information). This suggests the recombination of photogenerated carriers in FeOOH/Cu_2_O/ZnO catalyst is effectively inhibited.

The photon‐to‐current conversion efficiency, also known as incident photon‐to‐current efficiency (IPCE), of various photocathodes with varying wavelengths was measured (Figure 3b). The value of IPCE can be determined by employing the subsequent equation:

(1) $$\mathrm{IPCE}\left(\%\right)=\frac{\left(1240\cdot J_{\mathrm{light}}\right)}{\lambda P_{\mathrm{light}}}\;\times 100\%$$

where *J*_light_, *P*_light_ and λ are the photocurrent density, incident light irradiance and incident light wavelength (nm). Within the spectral range spanning 365 nm to 455 nm, every photocathode demonstrates a high quantum efficiency; nevertheless, as the wavelength surpasses 455 nm, there is a significant decline in the IPCE values. Compared with the Cu_2_O, FeOOH/Cu_2_O, and Cu_2_O/ZnO photocathodes, the IPCE of the FeOOH/Cu_2_O/ZnO photocathode reaches a maximum of up to 33.7%. This demonstrates that the introduction of the FeOOH HTL and ZnO ETL effectively enhances the photoelectric conversion of the FeOOH/Cu_2_O/ZnO photocathode and reduces the carrier recombination. Additionally, the integrated photocurrent densities of Cu_2_O, FeOOH/Cu_2_O, Cu_2_O/ZnO and FeOOH/Cu_2_O/ZnO are 0.31, 0.91, 1.55 and 2.61 mA·cm^−2^, respectively, which correspond to the photocurrent density in Figure 3a.

The applied bias photon‐to‐current efficiency (ABPE) is determined using photocurrent density measurements (Figure 3c). The equation below can be utilized to calculate the value of the ABPE.

(2) $$\mathrm{ABPE}\left(\%\right)=\frac{|J_{\mathrm{Iight}}|\times \left(V_{\mathrm{RHE}}-0\right)}{P_{\mathrm{light}}}\;\times 100\%$$

where *V*_RHE_, *J*_light_, and *P*_light_ are the applied potential, current density under illumination (300 W Xe lamp) and incident light power density. The ABPE of the FeOOH/Cu_2_O/ZnO photocathode reaches 0.35% at 0.297 V_RHE_, which is 11.7 times that of pure Cu_2_O photocathode (0.03% at 0.226 V_RHE_). FeOOH/Cu_2_O/ZnO achieves substantially enhanced performance compared to Cu_2_O/ZnO (0.14% at 0.283 V_RHE_) and FeOOH/Cu_2_O (0.07% at 0.262 V_RHE_). This improvement suggests that incorporating FeOOH and ZnO layers effectively optimizes charge carrier separation and transportation. Analysis of onset potentials across various photocathodes (Figure 3d) confirms that both FeOOH HTLs and the ZnO ETLs increase the photovoltage of the Cu_2_O photocathodes, thereby reducing the energy barrier required for PEC water reduction. Chopped light chronoamperometry measurements of the Cu_2_O photocathodes at 0 V_RHE_ (Figure 3e) reveal enhanced operational stability and photoresponse characteristics. Notably, the FeOOH/Cu_2_O/ZnO configuration demonstrates ≈12.5‐fold greater photocurrent density than bare Cu_2_O, indicating prolonged charge carrier lifetimes and improved charge separation efficiency. Electrochemical surface area (ECSA) evaluation through cyclic voltammetry (CV) measurements (0.7–0.8 V_RHE_) (Figure S21, Supporting Information) demonstrates a direct correlation between double‐layer capacitance and active site density. The linear slope magnitude in these measurements serves as a quantitative indicator of available catalytic sites, with steeper gradients corresponding to greater surface reactivity. The FeOOH/Cu_2_O/ZnO suggests the largest ECSA (9.89 µF·cm^−2^) compared to Cu_2_O/ZnO (9.35 µF·cm^−2^), FeOOH/Cu_2_O (8.71 µF·cm^−2^) and pristine Cu_2_O (7.59 µF·cm^−2^) (Figure 3f), which demonstrates the increased active sites induced by introducing the FeOOH and ZnO layers.

The Nyquist plots derived from electrochemical impedance spectroscopy (EIS) measurements for all four photocathode systems are presented in Figure 3g, and Figure S22 (Supporting Information) is the equivalent circuit. Quantitative parameters obtained through EIS analysis are detailed in Table S2 (Supporting Information). R_0_ and CPE and R_ct_ are the resistance of the electrolyte, constant phase element and electron transfer resistance. The findings indicate that the R_ct_ of the FeOOH/Cu_2_O/ZnO photocathode is 9.03 KΩ, which is much smaller than those of Cu_2_O/ZnO (16.0 KΩ), FeOOH/Cu_2_O (18.7 KΩ) and Cu_2_O (29.0 KΩ). This confirms that the FeOOH/Cu_2_O/ZnO photocathode facilitates accelerated interfacial charge transfer and efficient separation of photoexcited charge carriers. This process makes a significant contribution to the enhancement of PEC performance. Moreover, Bode phase plots (Figure 3h) for Cu_2_O and FeOOH/Cu_2_O/ZnO photocathodes are obtained from the impedance data (Tables S3 and S4, Supporting Information). Analysis of Bode phase diagrams reveals that the frequency corresponding to the peak maximum in Bode phase plots serves as a critical parameter for determining charge carrier lifetime (τ) through the following relationship.

(3) $$\tau =\frac{1}{2\pi f_{\max}}$$

where *f*_max_ and *τ* are the frequency at the peak maxima and the lifetime of the electron. The longevity parameters of charge carriers provide critical insights into recombination dynamics within the semiconductor structure and at the material‐electrolyte interface. Computational analysis yields charge carrier lifetimes of 0.16 ms for pristine Cu_2_O and 0.98 ms for the FeOOH/Cu_2_O/ZnO photocathode.

Figure 3i demonstrates that the FeOOH/Cu_2_O/ZnO photocathode exhibits a Tafel slope of 320.1 mV·dec^−1^, exhibiting a markedly lower value compared to Cu_2_O/ZnO (534.2 mV·dec^−1^), FeOOH/Cu_2_O (799.7 mV·dec^−1^), and pure Cu_2_O (868.9 mV·dec^−1^) within identical current density parameters. This suggests lower overpotential and faster reaction kinetics in the HER process. Operational durability represents a critical factor for implementing PEC systems, particularly when Cu_2_O‐based photocathode materials are used. The temporal variation in photocurrent density under sustained illumination conditions (0 V vs RHE) is presented in Figure 3j. The PEC stability of Cu_2_O and FeOOH/Cu_2_O/ZnO is further assessed utilizing steady‐state chronoamperometry. The photocurrent values on Cu_2_O rapidly decay and remain 31.3% after 1 h illumination. Nevertheless, the photocurrent on FeOOH/Cu_2_O/ZnO maintains 90.5% of its initial value after 10 h of continuous light exposure, which proves excellent stability. After 10 h of PEC test, the results of SEM, XPS, and XRD also demonstrate the high stability of the FeOOH/Cu_2_O/ZnO photocathode (Figures S12,S23 and S24, Supporting Information), which is superior to the stability of previously reported Cu_2_O‐based catalysts (Table S1, Supporting Information). The minor reduction and variation in photocurrent density observed over 10 h can be primarily attributed to the presence of bubbles adhering to the surface of photocathode.

Concurrently, the photoelectric performance of the synthesized FeOOH/ZnO/Cu_2_O has dramatically declined in comparison to the FeOOH/Cu_2_O/ZnO photocathode (Figure S25, Supporting Information), indicating that the sequence of these layers has an impact on performance. This also proves that ZnO as ETL on the FeOOH/Cu_2_O can promote the separation and transport of charges.

**Figure 4:**
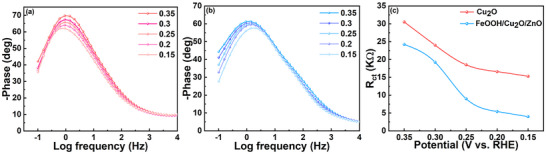
Bode phase plots of a) Cu_2_O and b) FeOOH/Cu_2_O/ZnO photocathodes at different potentials, and c) R_ct_ values derived from EIS data by fitting the equivalent circuits of photocathodes at different potentials.

The EIS curves of Cu_2_O and FeOOH/Cu_2_O/ZnO photocathodes at different application potentials are plotted, and the dependence of geometric configuration on HER dynamics is further studied. The magnitude of the phase Angle reflects how much charge is involved in the Faraday process. The smaller the phase Angle, the more charge is involved in the Faraday process. FeOOH/Cu_2_O/ZnO exhibits a smaller phase angle than Cu_2_O under the same bias, and this means that more of the charge on the FeOOH/Cu_2_O/ZnO surface is involved in the Faraday reaction, rather than being stored in the electrode/electrolyte interface (**Figure** **4**a,b). Therefore, the introduction of the FeOOH HTL and the ZnO ETL accelerates not only electron transport but also the reaction kinetics of the PEC water reduction. The charge‐transfer resistance (R_ct_) in the EIS plots follows the order Cu_2_O > FeOOH/Cu_2_O/ZnO under the same bias (Figure 4c), indicating that FeOOH/Cu_2_O/ZnO has smaller R_ct_ values than Cu_2_O. The curve shows the lower the potential, the lower the resistance, because the driving force for charge transfer and separation is enhanced by introducing FeOOH and ZnO. Therefore, under the reaction condition of the HER, especially a highly negative potential, PEC water reduction reaction of FeOOH/Cu_2_O/ZnO may occur. The experimental results confirm that FeOOH/Cu_2_O/ZnO photocathode can promote the surface electron transfer process and has excellent catalytic performance.

**Figure 5:**
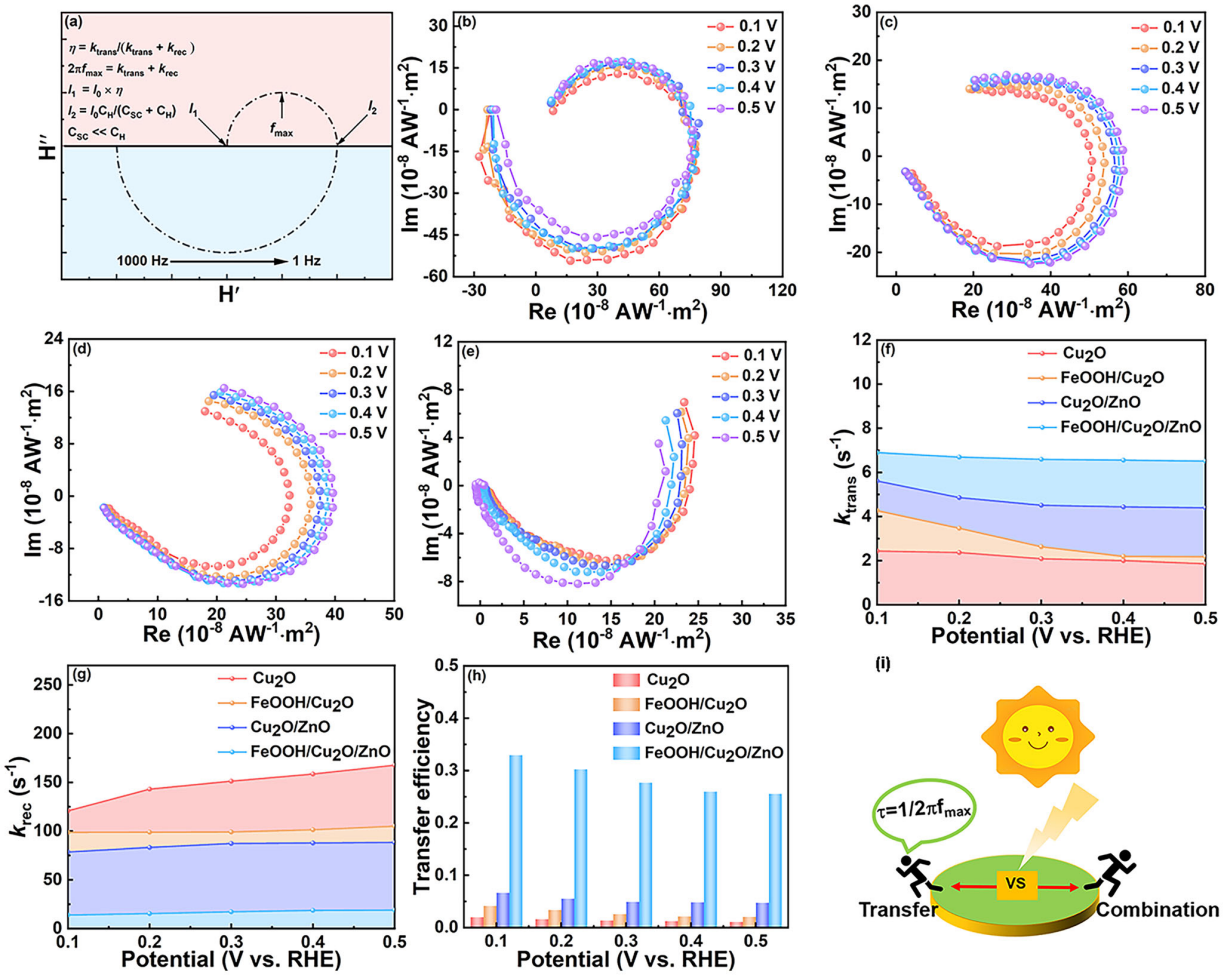
a) Calculated example of the intensity modulated photocurrent spectroscopy (IMPS), IMPS responses of b) Cu_2_O, c) FeOOH/Cu_2_O, d) Cu_2_O/ZnO and e) FeOOH/Cu_2_O/ZnO at various potentials, f) rate constants for charge transfer (*k*_trans_), g) rate constants for charge recombination (*k*_rec_), and h) charge transfer efficiencies extracted from the IMPS spectra of different photocathodes, and i) principle of the IMPS setup.

In order to reveal the mechanism of PEC water reduction process at the photoanode/electrolyte interface, the charge transfer process, surface carrier recombination kinetics, and the effects of FeOOH HTL and ZnO ETL on the adjustment of interface properties are analyzed by IMPS (**Figure** **5**; Figure S26, Supporting Information). The simplified expression for the perturbed photocurrent *j*(*ω*) associated with *k*_trans_ and *k*_rec_ is shown in Equations (4) and (5).

(4) $$\frac{j_{\mathrm{photo}}\left(\omega \right)}{j_{h}\left(\omega \right)}=\frac{k_{\mathrm{trans}}+i\omega \frac{C}{C_{\mathrm{SC}}}}{k_{\mathrm{trans}}+k_{\mathrm{rec}}+i\omega }\;\left(\frac{1}{1+i\omega RC}\right)$$

(5) $$C=\frac{C_{\mathrm{SC}}C_{H}}{C_{\mathrm{SC}}+C_{H}}\times 100\%$$

where *j*_h_(*ω*), *ω* and *R* are the periodic flux of photogenerated holes corresponding to the Gartner equation, light modulation frequency and total series resistance, *C* is the effective capacitance, *C*_H_ is the Helmholtz capacitance and *C*_SC_ is the space charge capacitance (*C_SC_* ≪ *C_H_*). Based on IMPS theory, with the frequency increasing, the relaxation of the photo‐generated hole concentration on the semiconductor surface is expressed by *f*_max_ (at the apex of the upper semicircle), where 2π*f*_max _ = *k_trans_* +*k_rec_*. The efficiency of charge transfer (*k_trans_*/(*k_trans_* + *k_rec_*)) is derived from the intersection of the semicircle and the real axis at low and high frequence (i.e., *I*_1_ and *I*_2_), where *I*_1_ = *I*_0_ *k_trans_*/(*k_trans_* + *k_rec_*) and *I*_2_ = *I*_0_ *C_H_*/(*C_SC_* + *C_H_*) (Figure 5a). Figure 5b‐e shows typical IMPS responses of different photocathodes under the applied potential region (0.1–0.5 V vs RHE). The two semi‐circles of the IMPS spectrum represent the competition between charge transfer and recombination (upper quadrant) and resistance‐capacitance attenuation (lower quadrant). A smaller ratio of *k_rec_*/*k_trans_* shows that charge transfer is faster than charge recombination, and it is proportional to the semicircular radius in the upper quadrant. The large upper semicircle radius of Cu_2_O photocathode indicates that there is serious charge recombination on the surface of Cu_2_O. In addition, with the decrease of applied potential, the change of upper semicircle radius is not obvious, indicating that Cu_2_O has rapid charge recombination at low potential. After the loading of FeOOH HTL and ZnO ETL on the Cu_2_O, with the decrease of applied potential, the upper semicircle radius further decreases, and the change is obvious. The semicircle radius of FeOOH/Cu_2_O/ZnO photocathode is the smallest, indicating that the charge transfer is accelerated and charge recombination is inhibited effectively. Interestingly, the surface recombination semicircle of FeOOH/Cu_2_O/ZnO disappears in IMPS response, and it further indicates that the presence of FeOOH and ZnO can effectively hold back the recombination of interface charge.

The values of *k*_trans_ and *k*_rec_ for photocathodes at different potentials are shown in Figure 5f,g. FeOOH/Cu_2_O/ZnO, Cu_2_O/ZnO and FeOOH/Cu_2_O own higher values of *k*_trans_ and lower values of *k*_rec_ than that of Cu_2_O photocathode under all applied potentials. FeOOH/Cu_2_O/ZnO has the lowest *k*_rec_ and highest *k*_trans_ among these photocathodes, proving that FeOOH HTL greatly accelerates the hole extraction from Cu_2_O, and ZnO ETL boosts the charge transfer to the surface in a wide range of applied potentials. Noteworthily, FeOOH/Cu_2_O/ZnO shows the highest charge transfer efficiency (Figure 5h), and it means higher PEC water reduction performance. These results demonstrate that FeOOH and ZnO, serving as the HTL and ETL, facilitate the transfer of electrons and holes in opposite directions.

**Figure 6:**
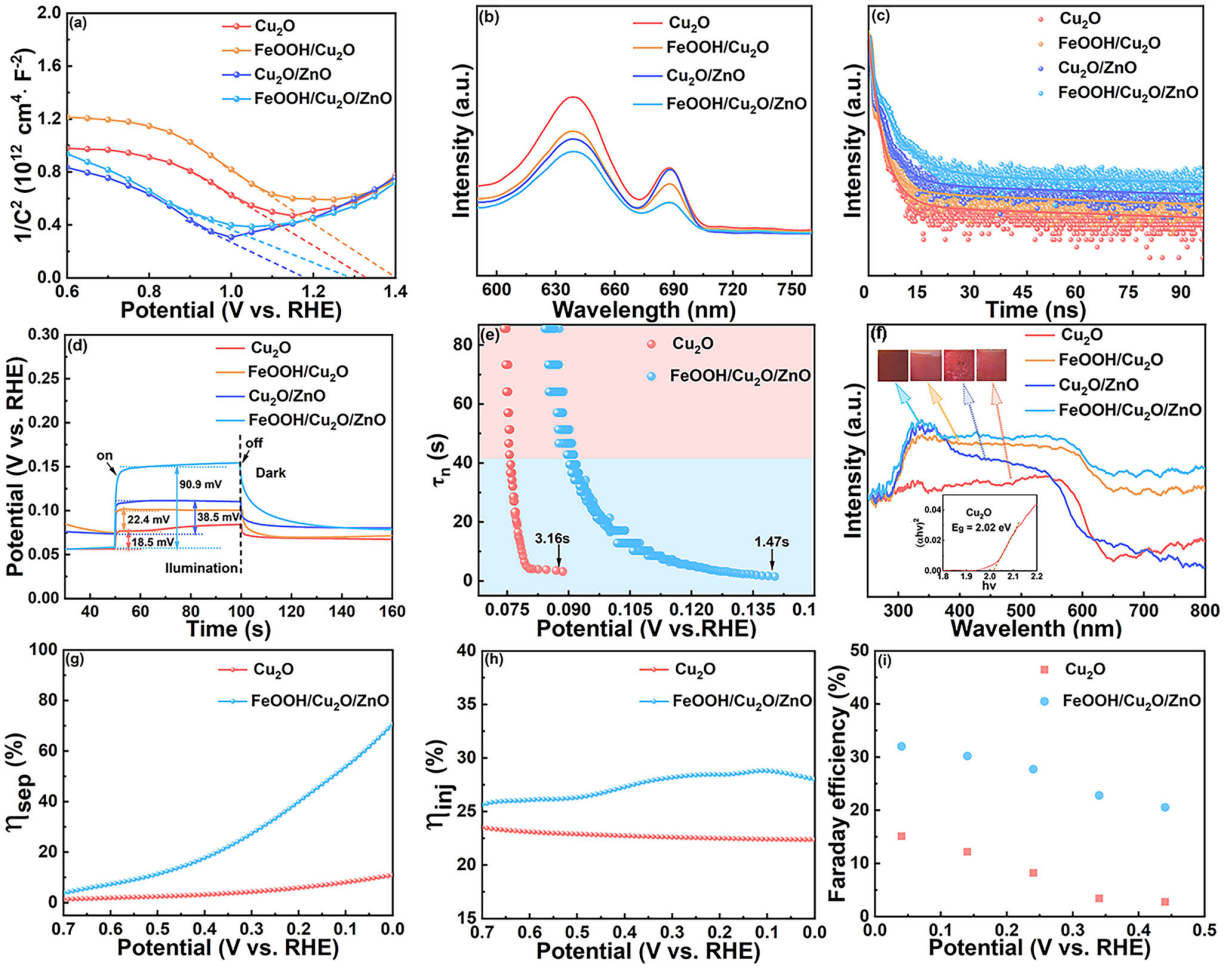
a) Mott‐Schottky (M‐S) plots measured at 1 kHz under dark condition, b) PL spectra, c) TRPL decay curves, d) OCP measurements under dark and light condition, e) carrier lifetime derived from the OCP decay curves, f) UV‐Vis diffuse reflectance spectra (the inset is band gap energy of Cu_2_O), g) photogenerated charge separation efficiency (𝜂_sep_), h) charge injection efficiencies (𝜂_inj_) and i) Faraday efficiency.

Additionally, to elucidate the cause of PEC enhancement, charge transfer kinetic analysis is conducted. The M‐S curves (**Figure** **6**a) of Cu_2_O, FeOOH/Cu_2_O, Cu_2_O/ZnO, and FeOOH/Cu_2_O/ZnO photocathodes are studied at 1 kHz as the following variable form of the M‐S equation:

(6) $$\frac{1}{C^{2}}=\frac{2}{e\varepsilon \varepsilon_{0}N_{d}A^{2}}\;\times \left[\left(V-V_{\mathrm{fb}}\right)-\frac{K_{B}T}{e}\right]$$

(7) $$N_{d}=\frac{2}{\mathrm{qe}\varepsilon \varepsilon_{0}}{\left(\left|\frac{d\left(\frac{1}{C^{2}}\right)}{dV_{s}}\right|\right)}^{-1}$$

where $\frac{d(\frac{1}{C^{2}})}{dV_{s}}$ is the slope of the M‐S plot, *C* is the space charge region capacitance, *ε* is the relative dielectric constant (for Cu_2_O is 18.1), *ε*_0_ is the absolute dielectric constant, *V* is the applied bias, *k*_B_ is the Boltzmann constant, *V*_fb_ is the flat band potential and *T* is the temperature. The carrier densities (*N*_d_) are inversely proportional to the slope of the M‐S plot. The negative slopes in the M‐S curves of all photocathodes indicate the typical features of p‐type semiconductors. Notably, compared with Cu_2_O photocathode, the flat band potential of FeOOH/Cu_2_O/ZnO and Cu_2_O/ZnO are significantly shifted to the negative direction, indicating that the recombination of photoexcited electron–hole can be suppressed.^[^ ^39^ ^]^ The slope of the FeOOH/Cu_2_O/ZnO is much smaller than those of the Cu_2_O, FeOOH/Cu_2_O and Cu_2_O/ZnO photocathodes. *N*_d_ are calculated based on the slopes: Cu_2_O (3.48 × 10^28^ m^−3^) < FeOOH/Cu_2_O (3.56 × 10^28^ m^−3^) < Cu_2_O/ZnO (4.37 × 10^28^ m^−3^) < FeOOH/Cu_2_O/ZnO (5.42 × 10^28^ m^−3^), which indicates that FeOOH as a hole transport and ZnO as an electron transport layer can significantly improve the photocathode's charge density and electronic conductivity.

To delve deeper into the efficiency of charge separation and photocarrier dynamics, photoluminescence spectroscopy (PL) measurements are conducted on the fabricated photocathodes, utilizing an excitation wavelength of 400 nm. FeOOH/Cu_2_O/ZnO photocathode has a much lower PL intensity (Figure 6b), proving the existence of FeOOH HTL and ZnO ETL effectively inhibits the charge recombination. In addition, the time‐resolved photoluminescence (TRPL) attenuation curve is drawn by fitting the double exponential function model, which can be used to decipher charge dynamics in real time (Figure 6c; Table S5, Supporting Information). Using Equation (8), an exponential decay model is employed to fit the TRPL date.

(8) $$\Delta A_{\mathrm{decay}}=A_{1}\;\exp\left(-\frac{t}{\tau_{1}}\right)+A_{2}\exp\left(-\frac{t}{\tau_{2}}\right)+\Delta A_{0}$$

The *τ*_1_ and *τ*_2_ are the lifetimes of the fast and slow decay processes. The fitted data of the intensity‐average lifetime of carriers are calculated based on the foumula (9).

(9) $$\tau =\left(A_{1}\tau_{1}^{2}+A_{2}\tau_{2}^{2}\right)/\left(A_{1}\tau_{1}+A_{2}\tau_{2}\right)$$

The lifetime of Cu_2_O photocathode is ≈3.70 ns, and the average carrier lifetimes increase to 4.21, 4.97 and 7.69 ns for FeOOH/Cu_2_O, Cu_2_O/ZnO and FeOOH/Cu_2_O/ZnO photocathodes. These results represent that the average carrier life is obviously prolonged, which promotes carrier separation and mobility for the interfacial reduction reaction.

To further reveal the charge transfer dynamics displayed by various photocathodes, open‐circuit potential (OCP) measurements are used to detect the surface recombination process. In addition, based on the OCP measurement, the carrier lifetime (*τ*) can be calculated from Equation (10).

(10) $$\tau =-\frac{k_{B}T}{e}{\left(\frac{dOCP}{dt}\right)}^{-1}$$

where *k*_B_T, *e*, and *τ* are thermal energy, positive element charge and carrier lifetime. The OCP difference of the FeOOH/Cu_2_O/ZnO photocathode (90.9 mV) is larger than those of the Cu_2_O/ZnO photocathode (38.5 mV), FeOOH/Cu_2_O photocathode (22.4 mV) and the Cu_2_O photocathode (18.5 mV), which further suggests that the FeOOH/Cu_2_O/ZnO photocathode has enhanced charge carrier separation efficiency and photovoltage (Figure 6d). The presence of FeOOH and ZnO as the HTL and ETL promotes the transport of electrons and increases the photovoltage of photocathode. After the light is turned off, the FeOOH/Cu_2_O/ZnO shows a transient carrier lifetime of 1.47 ms (Figure 6e), and it is less than that of Cu_2_O (*τ* = 3.16 ms). The results indicate that the incorporation of FeOOH HTL and ZnO ETL enables more efficient transport of photoinduced holes and electrons. This reduces the PEC reaction barrier of the photocathode and inhibits the recombination of surface carriers.

The light absorption edge of pure Cu_2_O is ≈650 nm, and it has a strong ability to capture visible light (Figure 6f). The tauc diagram for Cu_2_O is shown in the inset with a band gap of ≈2.02 eV. For as‐prepared Cu_2_O‐based photocathodes, the absorption edges have different levels of redshift. The FeOOH/Cu_2_O/ZnO has higher optical absorption from UV to visible light, and this is related to the deep color of FeOOH/Cu_2_O/ZnO. These results suggest that FeOOH/Cu_2_O/ZnO can increase PEC capacity by improving light utilization efficiency. Then, *η*_sep_ is determined for the FeOOH/Cu_2_O/ZnO photocathode. Using Na_2_S_2_O_8_ as sacrificial electrolyte, because the reduction kinetics of electron acceptor Na_2_S_2_O_8_ is very fast, the photocurrent separated (=*J*_sep_) is all involved in the cathode reaction. Therefore, *η*_sep_ can be determined from the ideal photocurrent of photocathode (= *J*_abs_) and measured photocurrent in Na_2_S_2_O_8_ electrolyte (=*J*_sep_) (Figure S27, Supporting Information).

For FeOOH/Cu_2_O/ZnO photocathode, the quantum efficiency for *η*_sep_ and the ratio of the separated electrons used for chemical reactions is calculated using the following Equations (11)‐(14).

(11) $$J_{\mathrm{abs}}=J_{\mathrm{sep}}+J_{\mathrm{br}}$$

(12) $$J_{\mathrm{sep}}=J_{\mathrm{ir}}+J_{\mathrm{FE}}$$

(13) $$\eta_{\mathrm{sep}}=J_{\mathrm{sep}}\;/J_{\mathrm{abs}}$$

(14) $$\mathrm{FE}\;=J_{\mathrm{FE}}\;/J_{\mathrm{sep}}$$

As illustrated in Figure S28 (Supporting Information), *J*_sep_ represents the part of photogenerated current that are transported to the semiconductor surface. It is measured by LSV in 0.5 M Na_2_S_2_O_8_ sacrificial agent. All the charges transferred to the surface of photocathode can be detected by the LSV, assuming interfacial recombination is fully suppressed (*J*_ir_ = 0). *J*_br_ and *J*_ir_ are photocurrents consumed by bulk recombination and surface recombination respectively. *J*_FE_ is the photocurrent used for Faradaic reactions, measured by LSV in 0.5 M Na_2_SO_4_ electrolyte for PEC water reduction. *J*_abs_ is the ideal photocurrent density (−9.1 mA·cm^−2^) calculated from UV‐vis absorption spectra of Cu_2_O under AM 1.5G solar irradiation and an assumed absorbed photon conversion efficiency of 100%.

FeOOH/Cu_2_O/ZnO photocathode has a high initial potential of 0.98 V and a high η_sep_ of 4.54 ≈ 71.2% at PEC water reduction potential (Figure 6g). This indicates the superior ability of the incorporation of FeOOH HTL and ZnO ETL to effectively extract the photogenerated charge carriers from the Cu_2_O. What is more, *η*_inj_ can be obtained from Equation (15).

(15) $$\eta_{\mathrm{inj}}=J_{H_{2}O}\;/J_{\mathrm{sep}}$$

*J*_H2O_ and *J*_sep_ are the photocurrent densities in Na_2_SO_4_ solution (0.5 M) without and with the addition of Na_2_S_2_O_8_ sacrificial agent (0.5 M). Hence, the photocurrent density (*J*_H2O_) of PEC water reduction under electrolyte solution (without e^−^ scavengers) can be expressed by the following equation:

(16) $$J_{H_{2}O}=J_{\mathrm{abs}}\times \eta_{\mathrm{sep}}\;\times \eta_{\mathrm{inj}}$$

FeOOH/Cu_2_O/ZnO has a higher 𝜂_inj_ charge injection capacity (at 0.11 V_RHE_, 29.0%) at a relatively low potential than Cu_2_O (at 0.7 V_RHE_, 23.6%) (Figure 6h). This is consistent with the higher onset potential of the electrode, and indicates that the ZnO layer facilitates charge separation. As the potential is increased, the 𝜂_inj_ tends to be stable and no longer increases significantly, proving that the water reduction reaction is close to saturation (Figure S29, Supporting Information).

During the PEC water reduction test, the percentage of separated photocurrent used for surface Faradaic reaction (Figure 6i), expressed as Faraday efficiency (FE), is ≈32.1% for FeOOH/Cu_2_O/ZnO photocathode at 0 V_RHE_, and it is 2 times higher than that of the Cu_2_O photocathode (FE = 15.2%). Notably, in the Cu_2_O photocathode, more than 84% of the photogenerated electrons are lost. This is because of severe photogenerated electron–hole recombination and the relatively sluggish H_2_O reduction reaction kinetics during the PEC process. Therefore, these results verify that the loading of FeOOH and ZnO as the HTL and ETL in the FeOOH/Cu_2_O/ZnO system synergistically promotes charge separation and transport, thus improving the PEC performance.

**Figure 7:**
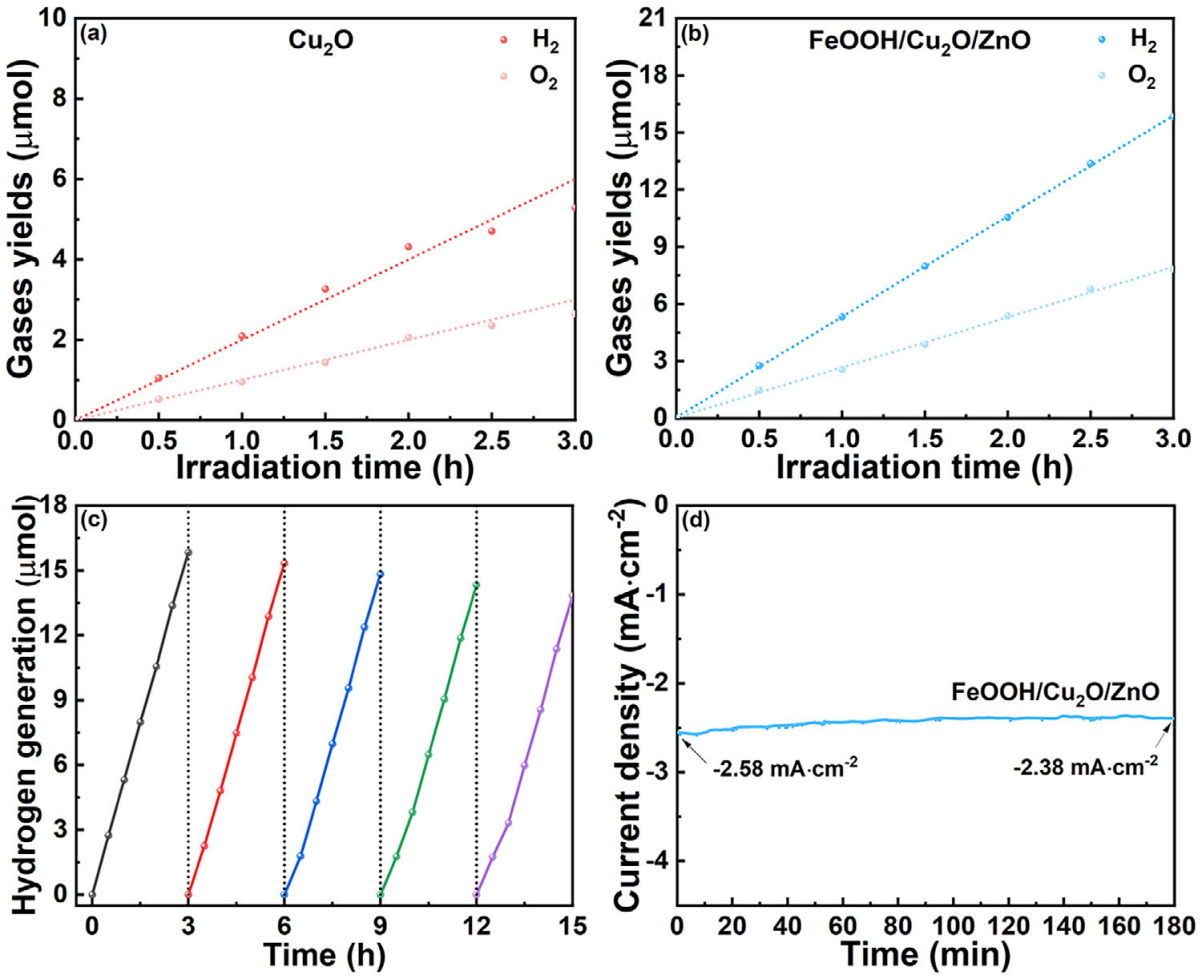
PEC water reduction test of a) Cu_2_O, b) FeOOH/Cu_2_O/ZnO (the dotted lines in the Figure indicate theoretical gas generation and the dots indicate actual gas generation), c) hydrogen generation on FeOOH/Cu_2_O/ZnO photocathode at potential of 0 V versus RHE under illumination of simulated solar light, and d) long‐term PEC stability test.

In order to further clarify the stability of Cu_2_O based photocathodes and the performance of PEC water resolution hydrogen and oxygen evolution, a three‐electrode system is used to carry out the PEC water decomposition reaction, and the water decomposition products are analyzed qualitative and quantitative in real‐time by gas chromatography (**Figure** **7**a,b; Figure S30, Supporting Information). The total amount of H_2_ and O_2_ produced by FeOOH/Cu_2_O/ZnO photocathodes within 3 h is 15.84 and 7.78 umol, which is higher than those of Cu_2_O photocathode. The stoichiometric ratio of H_2_ to O_2_ produced in each time node is ≈2:1, which is consistent with the total decomposition reaction of water. The above results indicate that FeOOH/Cu_2_O/ZnO photocathode has excellent PEC water decomposition performance. The H_2_ production experiments are repeated for 5 cycles, each for 3 h, and an impressive H_2_ generation rate of 13.5 µmol· h^−1^·cm^−2^ is obtained (Figure 7c). Long‐term PEC stability tests on FeOOH/Cu_2_O/ZnO are evaluated with a steady‐state chronoamperometry under light (Figure 7d). After 3 h of testing, the high optical current density does not decrease significantly. The shapes of XRD and Raman peaks as well as micromorphology before and after PEC measurements are similar, and the peak positions have not changed obviously, indicating the FeOOH/Cu_2_O/ZnO photocathode still maintains excellent stability after water reduction reaction (Figures S31 and S32, Supporting Information). In general, FeOOH as HTL and ZnO as protective layer, ETL and kinetic catalytic layer contribute to efficient PEC water reduction hydrogen production.

**Figure 8:**
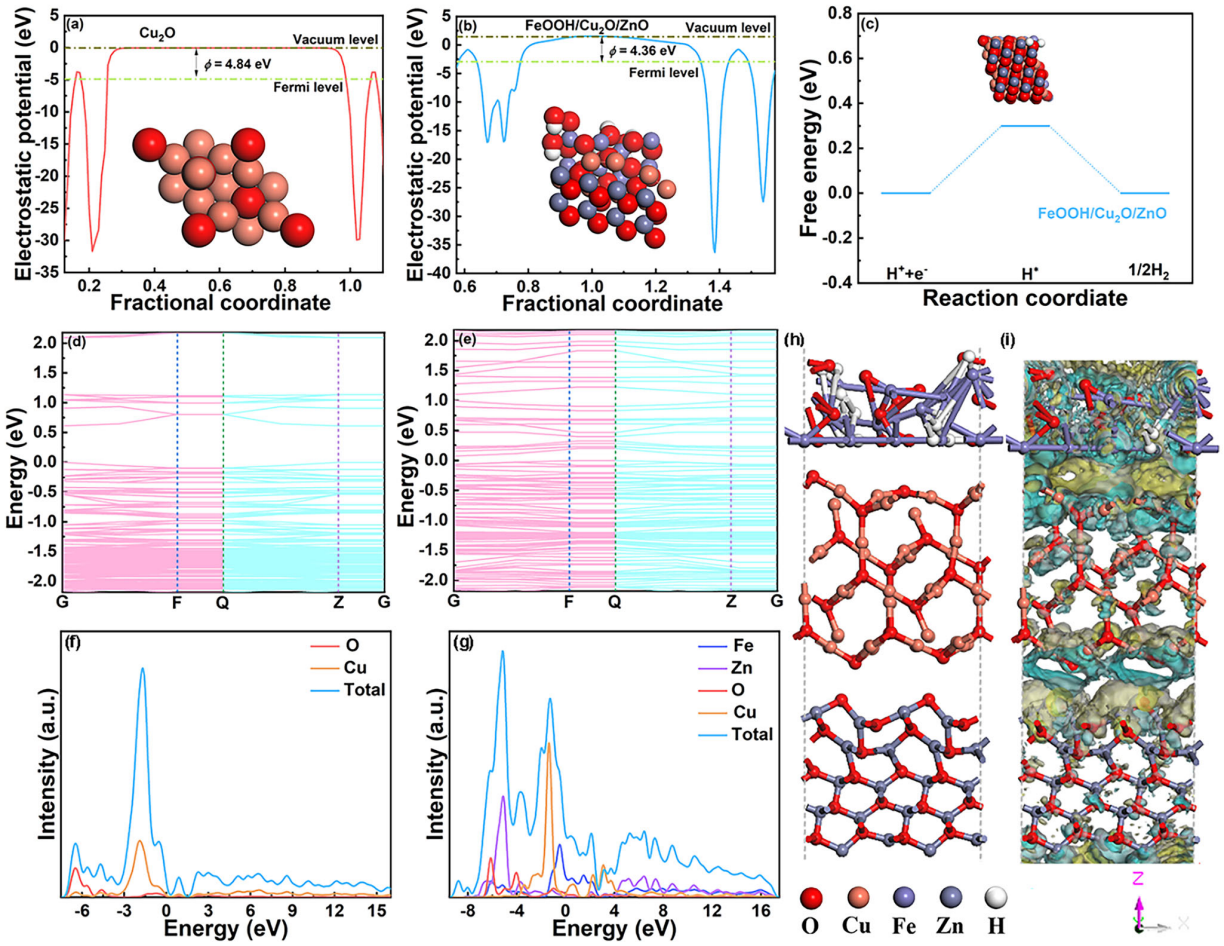
The electrostatic potential distribution of a) Cu_2_O and b) FeOOH/Cu_2_O/ZnO, c) HER reaction steps (Inset images are the corresponding optimal theoretical structure), the energy band structure of d) Cu_2_O and e) FeOOH/Cu_2_O/ZnO, PDOS of f) Cu_2_O and g) FeOOH/Cu_2_O/ZnO h) optimal theoretical structure of FeOOH/Cu_2_O/ZnO along Y direction and i) charge density difference of FeOOH/Cu_2_O/ZnO along Y direction.

In order to further reveal the mechanism of PEC performance enhancement, the electronic structure property was calculated using DFT. The work functions of Cu_2_O and FeOOH/Cu_2_O/ZnO are revealed by electrostatic potential distribution along Z direction, where the FeOOH/Cu_2_O/ZnO suggests a smallest work function (ϕ) (4.36 eV) among these photocathodes (**Figure** **8**a,b; Figure S33, Supporting Information). The ϕ follows Equation (17):

(17) $$\phi =E_{\mathrm{vac}}\;-E_{F}$$

The *E*_F_ and *E*_vac_ represent the Fermi level and vacuum level. The vacuum energy level is the standard, and the Fermi level of FeOOH/Cu_2_O/ZnO downshifts after FeOOH and ZnO introduction, and this facilitates the transfer of electrons through the catalyst‐electrolyte interface.

The ultraviolet photoelectron spectroscopy (UPS) plot is further used to verify the calculation. *E*_cutoff_ of Cu_2_O is 15.52 eV and *E*_cutoff_ of FeOOH/Cu_2_O/ZnO is 15.46 eV.

(18) $$\phi \;=21.22-\left|E_{\mathrm{cutoff}}-E_{\mathrm{fermi}}\right|$$

According to the Equation (18), the work function of Cu_2_O is calculated to be 6.10 eV (relative to the vacuum level), here, 21.22 eV is the energy of the UV excitation source. Figure S34 (Supporting Information) suggests that Cu_2_O has a larger work function compared to FeOOH/Cu_2_O/ZnO, and it is consistent with the theoretical calculation results in Figure 8a,b.

In addition to the aforementioned findings, the free energy, a vital part associated with the HER route of catalysts (Figure S35, Supporting Information), is estimated to analyze the H_2_ generation activities. The closer the free energy is to zero, the smaller the barrier in the reaction. For Cu_2_O modified by FeOOH HTL and ZnO ETL, the free energy is lower those of FeOOH/Cu_2_O, Cu_2_O/ZnO and Cu_2_O (Figure 8c; Figure S36, Supporting Information), which is therefore thermodynamically favorable for H* formation and for fast HER dynamics. This structure effectively lowers the reaction barriers and produces high intrinsic activity for HER. The Cu_2_O suggests a typical indirect band gap, while the FeOOH/Cu_2_O/ZnO shows a state similar to direct band gap (Figure 8d,e; Figure S37, Supporting Information). The band gap width of FeOOH/Cu_2_O/ZnO is obviously narrowed, and it is conducive to the separation of electron–hole pairs.

The density of states (DOS) of photocathodes are shown in Figure 8f,g and Figure S39 (Supporting Information). In the presence of FeOOH and ZnO layers, a new strong DOS peak appears near −1.0 eV for Cu 3d, indicating that the higher the energy level of the Cu 3d atomic orbital, the stronger the chemical redox ability. After FeOOH and ZnO introduction, both Fe sites and Zn sites indicate the decreased energy band center, and it is consistent with the average valence increase in XPS spectra and the increase of charge carriers. The optimized atomic configuration of the composite is visualized in Figure 8h, which is excellent correlation with the cross‐sectional SEM images of the real photocathodes. The differential charge density values clearly show the remarkable distribution of electron clouds at the interface (Figure 8i; Figure S40, Supporting Information). The yellow electron cloud indicates electron accumulation, while the cyan electron cloud represents electron dissipation. The electron accumulation and dissipation indicate a solid foundation for forming a stable interface through substantial electron transfer at the interface. The electron depletion region appears around the Cu atoms, while, the electron enrichment region is located above the adjacent Zn atoms. The local electric polarization is thermodynamically favorable for the FeOOH and ZnO layers to strongly combine with H_2_O molecules. Thus, it is conducive to the dissociation of hydrogen‐oxygen bonds and the rapid reduction of water. Furthermore, compared with FeOOH/Cu_2_O and Cu_2_O/ZnO, the charge density of FeOOH/Cu_2_O/ZnO photocathodes increases significantly, which indicates the introduction of FeOOH HTL and ZnO ETL can provide more charge carriers to directly participate in the surface HER reaction.

The surface hydrophilic‐hydrophobic properties of the Cu_2_O and FeOOH/Cu_2_O/ZnO photocathodes are characterized through measuring the contact Angle (CA) (Figure S41, Supporting Information). As expected, the unmodified Cu_2_O photocathode exhibits a CA value of 48.5°, a typical surface hydrophilic feature. On the FeOOH/Cu_2_O/ZnO film, the CA value decreases to 18.5°. The FeOOH/Cu_2_O/ZnO photocathode is essentially fully wet in the aqueous solution environment. The good surface wettability of FeOOH/Cu_2_O/ZnO photocathode in aqueous solution is a prerequisite before further water reduction reaction can occur, facilitating the adsorption of H_2_O molecules. As expected, the energy of H_2_O molecules is discrete before adsorption, but continuous after adsorption on Cu_2_O and FeOOH/Cu_2_O/ZnO, indicating that electrons are transferred to the antibonding orbitals of H_2_O molecules to participate in the water splitting (Figures S42 and S43, Supporting Information). Among them, the occupied electron orbitals of H_2_O on FeOOH/Cu_2_O/ZnO (111) show the most obvious shift to the left, indicating that the electrons on FeOOH/Cu_2_O/ZnO (111) are more easily transferred to the antibonding orbitals of H_2_O molecules. The H_2_O molecules on FeOOH/Cu_2_O/ZnO (111) also have greater adsorption energy. These evidences prove that the optimized adsorption strength for H_2_O molecules and the intermediate of HER process can be effectively achieved via the FeOOH HTL and ZnO ETL introduction, and the charge transfer can be increased, reducing the reaction energy barrier.

**Figure 9:**
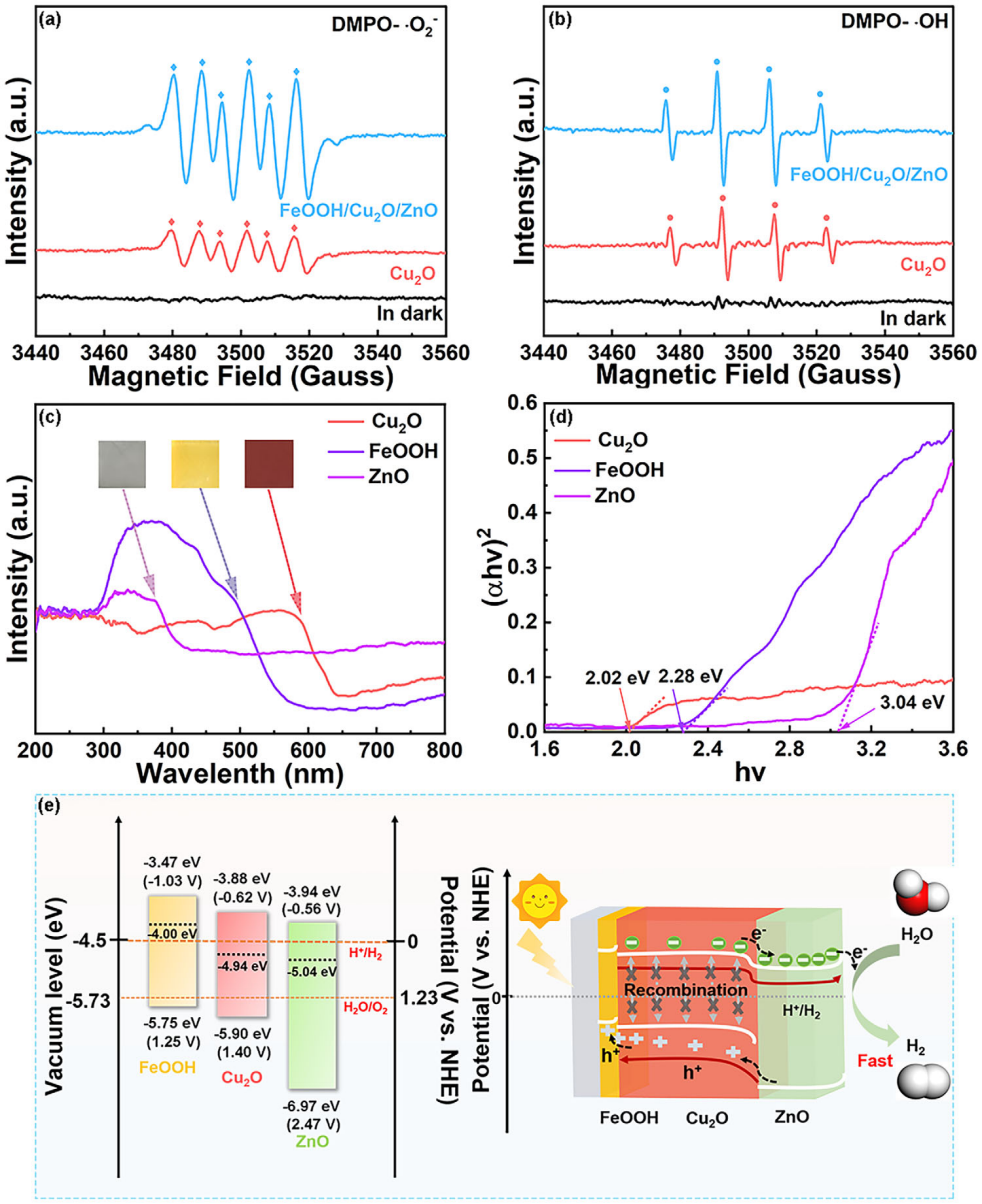
Spin‐trapping EPR spectra: a) DMPO‐·O_2_^−^ and b) DMPO‐·OH, c) the UV‐Vis diffuse reflectance spectra, d) the band gap energy, and e) energy diagrams and schematic diagram of the photogenerated carrier transport behavior in the FeOOH/Cu_2_O/ZnO photocathode.

The electron paramagnetic resonance (EPR) test is carried out to reveal the charge transfer mechanism in the PEC process, and 5,5‐dimethyl‐1‐pyrroline‐N‐oxide (DMPO) serves as a spin capture reagent to detect ·O_2_^−^ or ·OH. There are no peaks corresponding to ·O_2_^−^ and ·OH without light irradiation. The typical six peaks verify that ·O_2_^−^ is produced as an active substance in the PEC process (**Figure** **9**a). Compared with Cu_2_O, FeOOH/Cu_2_O/ZnO shows the strong DMPO‐·O_2_^−^ characteristic peaks. The redox potentials of O_2aq_/·O_2_^−^ (−0.16 V vs NHE) and O_2g_/·O_2_^−^ (−0.33 V vs NHE) are larger than the E_CB_ of ZnO (−0.35 V vs NHE), and FeOOH/Cu_2_O/ZnO demonstrates enhanced oxygen activation by increased ·O_2_^−^ intensity and the induced electrons accumulated on the conduction band (CB) of ZnO can generate sufficient ·O_2_^−^. Thus, electrons will flow from Cu_2_O to ZnO in the FeOOH/Cu_2_O/ZnO photocathode. The standard quartet of four equidistant peaks associated with DMPO‐·OH is detected under light (Figure 9b). The ·OH characteristic signal intensity generated by Cu_2_O is 1.5 times lower than that of FeOOH/Cu_2_O/ZnO. For FeOOH/Cu_2_O/ZnO photocathode, the increased ·OH evolution could attribute to the H_2_O oxidization. Because the oxidation capacity of valence band (VB) for hole generation in Cu_2_O is not enough to convert OH^−^ to ·OH (2.37 V vs NHE), Cu_2_O will not be able to produce ·OH. As a result, the weak DMPO‐ ·OH signal in Cu_2_O is caused by a small number of ·OH radicals originated from the oxidization of H_2_O by O_2_^−^. According to these results, it can be shown that ·O_2_^−^ plays the major role in the PEC process, and ·OH is the second most important active specie. Therefore, FeOOH/Cu_2_O/ZnO has strong DMPO‐·O_2_^−^ and DMPO‐·OH signals, which attribute to the improvement of the electron–hole pair separation efficiency and electron migration efficiency.

From the UV‐Vis absorption spectra and Tauc plots of FeOOH, Cu_2_O and ZnO (Figure 9c,d), the bandgaps of FeOOH, Cu_2_O and ZnO are ≈ 2.28, 2.02 and 3.04 eV. The positive slopes in the M‐S curves of FeOOH and ZnO photoelectrodes indicate the typical features of n‐type semiconductors (Figure S44, Supporting Information). The mechanism proposed for charge transfer in the FeOOH/Cu_2_O/ZnO photocathode is show in Figure 9e. The band edge positions of FeOOH, Cu_2_O, and ZnO are critical to understanding the charge transfer and substance formation mechanisms involved in PEC reactions. The valence band maximum (VBM) positions measured by UPS (Figure S45, Supporting Information) are consistent with the MS results. The positions of the CB and VB of FeOOH, Cu_2_O and ZnO are calculated with the following equations:

(19) $$E_{\mathrm{CB}}=E_{\mathrm{VB}}\;-E_{g}$$

Under light, the photoelectrons and holes are produced in the CB and VB of Cu_2_O. The high electron–hole recombination rate of bare Cu_2_O greatly limits the PEC water reduction efficiency (Figure S46, Supporting Information). In integrated FeOOH/Cu_2_O/ZnO photocathode, due to the drive of the hole storage/transport layer, ETL and the favorable energy level arrangement, the photogenerated holes are transferred to the substrate through the FeOOH hole extraction, and the photoinduced electrons spontaneously migrate to ZnO. So, the dissociation and migration efficiency of photoexcited carriers are obviously enhanced (Figure 9e). Furthermore, thermodynamically speaking, the matching energy levels are also beneficial for HER.

## 3 Conclusion

In summary, we have developed a durable and efficient Cu_2_O‐based photocathode system for PEC water reduction. The FeOOH and ZnO are selected as hole storage/transport layer, electron transport layer and protective layer, which boost separation and transport of photoinduced charge carriers and effectively inhibits photocorrosion of Cu_2_O. Therefore, the FeOOH/Cu_2_O/ZnO photocathode shows excellent PEC water splitting performance and excellent stability. The maximum photocurrent density of reaches −2.54 mA cm^−2^ at 0 V_RHE_, and it is 12.7 times greater than that of Cu_2_O photocathode (−0.2 mA cm^−2^). At 0 V_RHE_ (455 nm), the IPCE reaches 33.7%, and it is 8.1 times greater than that of pure Cu_2_O (4.18%). DFT calculations further reveal that this special sandwich structure is favorable for charge transport, enhances the adsorption of water molecules and reduces the free energy of hydrogen production, thus boosting PEC hydrogen production activity. This work provides a good guide for the design and construction of PEC materials under feasible process conditions.

## 4 Experimental Section

### Materials

Fluorine‐doped tin oxide (FTO) was bought from Wuhan Jingge Solar Technology Co. Ltd. Copper sulfate pentahydrate (CuSO_4_·5H_2_O), DL‐Lactic acid (C_3_H_6_O_3_), sodium hydroxide (NaOH), sodium sulfate (Na_2_SO_4_), 5,5‐dimethyl‐1‐pyrroline N‐oxide, zinc nitrate hexahydrate (Zn(NO_3_)_2_·6H_2_O) and ferric chloride hexahydrate (FeCl_3_·6H_2_O) were bought from Macklin firm, China. All aqueous solution was prepared using deionized (DI) water. None of the chemicals and reagents have been further purified.

### Preparation of FeOOH

The preparation of FeOOH was referred to the reported literature.^[^ ^40^ ^]^ FTO glass substrates (7 Ω, active area 10 × 15 × 2.2 mm, light transmittance 80%) used in the experiment were ultrasonically cleaned in ethanol, acetone, and DI water before use. 0.4055 g of FeCl_3_·6H_2_O and 1.4204 g of Na_2_SO_4_ were dissolved in 10 mL of DI water and magnetically stirred for 30 min. After that, the solution was added into a Teflon‐lined steel autoclave reactor. The FTO glass substrate was placed in the reaction vessel with the conductive side facing down. The autoclave was maintained at 100 °C (4 h), and then cooled down to the room temperature. Subsequently, the sample was taken out, washed, and dried.

### Preparation of FeOOH/Cu_2_O Photocathode

The three‐electrode system was applied in the process of electrodepositing Cu_2_O on FTO glass substrate (Pt counter electrode, Ag/AgCl reference electrode and FTO working electrode with the area of 1.0 cm^2^). The solution used for electrodeposition of Cu_2_O was obtained by mixing 20 mL of lactic acid (3 M) and 20 mL of CuSO_4_ (0.4 M), and the pH was adjusted to 11.5 by NaOH (5 M). The configured electrolyte was deoxygenated in a nitrogen atmosphere for 20 min. The electrodeposition potential was controlled at −0.5 V for 15 min. During deposition, the electrolyte was maintained at different temperatures (55, 60, and 65 °C, respectively). After the electrodeposition, the surface of the Cu_2_O photocathode was washed, and then dried in vacuum at 60 °C. Notably, pristine Cu_2_O became smaller and more uniform at 60 °C (Figures S3 and S4, Supporting Information). So, the FeOOH/Cu_2_O photocathode was prepared at 60 °C with other experimental parameters remained unchanged, and the working electrode was changed to FTO/FeOOH. To assist in the deposition of Cu_2_O, the FTO/FeOOH photoelectrodes were exposed to the designated temperature for a series of durations (10, 15, and 20 min). The samples of varying thicknesses obtained at 60 °C were referred to as FeOOH/Cu_2_O‐10, FeOOH/Cu_2_O‐15 and FeOOH/Cu_2_O‐20 based on the electrodeposition time (Figure S7, Supporting Information). While compared to other photocathodes, FeOOH/Cu_2_O‐15 exhibited superior PEC performance (Figure S8a, Supporting Information). Therefore, optimal FeOOH/Cu_2_O photocathodes were prepared under the condition of 60 °C during 15‐min sedimentation time for subsequent experiments.

### Preparation of FeOOH/Cu_2_O/ZnO Photocathode

The three‐electrode system has been applied in the process of electrodepositing ZnO on optimal FeOOH/Cu_2_O. The solution used for electrodeposition was obtained by dissolving 1.4875 g of Zn(NO_3_)_2_·6H_2_O in 100 ml of DI water at 70 °C, and the pH was adjusted to 5.5. The electrodeposition potential was controlled at −1.0 V for several durations (100, 150, and 200 s) while the electrolyte bath was concurrently held at a constant temperature of 70 °C. According to the electrodeposition time, the samples of different thicknesses obtained at 60 °C were labeled as FeOOH/Cu_2_O/ZnO‐100, FeOOH/Cu_2_O/ZnO‐150 and FeOOH/Cu_2_O/ZnO‐200 (Figure S14, Supporting Information). Notably, FeOOH/Cu_2_O/ZnO‐150 exhibited the best PEC performance compared with other photocathodes (Figure S8b, Supporting Information). For the purpose of the next studies, ideal FeOOH/Cu_2_O/ZnO photocathodes were thus made at 70 °C for 150 s of sedimentation. After the electrodeposition, the surface of the optimal FeOOH/Cu_2_O/ZnO photocathode was washed and dried in vacuum at 60 °C. For comparison, pure ZnO photocathode was also prepared under the same condition, and FTO glass was used as the working electrode.

### Characterization Techniques

The crystalline structure of synthesized samples was analyzed using XRD(D8‐ADVANCE). Raman spectral data was collected using a Renishaw InVia system (532 nm excitation source). UV‐3600 spectrophotometry measurements were performed to evaluate the optical absorption characteristics of the synthesized materials across 250–800 nm wavelengths. Morphological characteristics and elemental composition were examined through SEM(JSM‐7500F) and TEM(JEM‐2100F) coupled with EDS（JEM‐F200). Surface chemical states and electronic properties were investigated using XPS and UPS(ESCALABMK‐MKII). Photoluminescence properties were investigated through steady‐state PL and TRPL measurements conducted on an FLS920 spectrometer. The electron paramagnetic resonance spectrometer (Bruker EMXplus‐6/1) was used to performEPRmeasurements with water or methanol in the presence of DMPO.

The details of computational methods, PEC measurements, and PEC water splitting test over these prepared samples can be found in the Supporting Information.

## Conflict of Interest

The authors declare no conflict of interest.

## Supporting information



Supporting Information

## Data Availability

Research data are not shared.
