# Supplementary material for: Rapid Charge Extraction via Hole and Electron Transfer Layers on Cu2O Photocathode for Stable and Efficient Photoelectrochemical Water Reduction
Source: Adv Sci (Weinh). 2025 Jul 30;12(40):e09030. doi: 10.1002/advs.202509030 (PMC12561211; doi:10.1002/advs.202509030)
Supplement: Supplementary file 1 — Supporting Information [file ADVS-12-e09030-s001.docx]

Supporting Information

**Rapid Charge Extraction via Hole and Electron Transfer Layers on Cu_2_O Photocathode for Stable and Efficient** **Photoelectrochemical Water Reduction**

*Shuangshuang Huai, Xiang Li, Ping Li, Shijian Zhang, Xiuxiu Huang, Wenbin Ruan, Jianli Chen, Zhi Tang, Xiaoli Zhao,^*^ Hewen Liu,**^*^ Xiufang Wang^*^*

S. Huai, X. Li, S. Zhang, X. Huang, W. Ruan, J. Chen, P. Li, X. Wang

Anhui Province Key Laboratory of Advanced Building Materials

Anhui Jianzhu University

Hefei, 230601, China

E-mail: wxfrye159@sina.com

J. Chen, H. Liu

School of Chemistry and Materials Science

University of Science and Technology of China

Hefei, 230026, China

E-mail: lhewen@ustc.edu.cn

Z. Tang, X. Zhao

State Key Laboratory of Environmental Criteria and Risk Assessment

Chinese Research Academy of Environmental Sciences

Beijing, 100085, China

E-mail: zhaoxiaoli_zxl@126.com

**Experimental Section**

**1.** **Computational methods**

DFT calculation was built in Materials Studio and was utilized to do all calculations. The structural relaxations were performed using the *ab initio* calculations of it. To optimize the structural model and calculate energy, Ultrasoft pseudopotentials with valence electrons 1*s*^1^ for H, 2*s*^2^2*p*^4^ for O, 3*d*^6^4*s*^2^ for Fe, 3*d*^10^4*s*^1^ for Cu, and 3*d*^10^4*s*^2^ for Zn were employed with a kinetic cutoff energy 400 eV. The Birllouin zone was sampled with a *k*-point mesh of 2 × 2 × 1 to ensure that the enthalpy calculations converge to less than 1 meV/atom. The exchange-correlation functional was described using the Perdew-Burke-Ernzerhof of generalized gradient approximation (PBE-GGA). In particular, DFT-D functional of TS is used to account for the vander Waals interaction. To avoid interactions between periodic pictures, a vacuum zone of 20 Å was introduced normally to the surface.

By adjusting the adsorption energy of H*, one may get the indicator of HER performance known as the adsorption free energy of H* (${\Delta G}_{H}$). For describing the adsorption state of H atoms, as specified by equation (1), the differential binding energy was often used:

$\Delta E_{H}=E_{\mathrm{adsorption}}-E_{\mathrm{surfance}}-1/{2E_{H_{2}}}$ (1)

where *E*_adsorption_, *E*_surfance_ are the total energy for the photocatalyst with or without one adsorbed H atoms, and *E*_H2_ represents the energy of the H_2_ molecule.

The Gibbs free energy was calculated using the following equation:

${\Delta G}_{H}^{*}=\Delta E_{H}+0.24eV$ (2)

**2.** **PEC measurements**

A conventional three-electrode cell was performed to examine PEC on the electrochemical workstations Zennium (Zahner-Electrik, Germany) and CHI760E (Shanghai Chenhua, China), where Pt, Ag/AgCl, and sample-coated FTO were treated as the reference, auxiliary, and photocathode, respectively. 0.5 M of Na_2_SO_4_ and a 300 W Xe lamp were served as the electrolyte and light source. To obtain a well-adhered coating, the working electrode was dried in the air for 12 h at 60 °C. The following equation (3) can be applied to derive the E_RHE_.

$E_{\mathrm{RHE}}=E_{Ag/AgCl}+0.0592\mathrm{pH}+0.197$ (3)

**3. PEC water splitting test**

The PEC reaction occurred in a sealed quartz reactor equipped with a typical three-electrode cell connected to an Ar cycle and a cooling device. In this cell, Na_2_SO_4_ (0.5 M) and a 300 W Xe lamp were served as the electrolyte and light source. The catalyst-coated FTO and Pt wire were used as the photocathode and the auxiliary electrode. The reference electrode was a saturated calomel electrode. Before lighting, pure Ar was injected into the device to ensure that the whole process happened in a noble gas atmosphere. A vacuum pump was then used to extract the gas from the experimental system in order to produce a negative-pressure atmosphere about -100 KPa. A recirculated cooling system maintained a 4 °C temperature in the reaction device. Approximately 10 cm away from the reactor, a 300 W Xe lamp was used to provide light. Simultaneously, the electrochemical workstation supplied a -0.5 V_RHE_ DC bias. The thermal conductivity detector and 5 Å molecular sieve column of the gas chromatograph (Mc-SCX300P) were both equipped with the carrier gas Ar. It was used to investigate the gas generated by the PEC process, and a known volume of pure H_2_ was used to calibrate the standard gas chromatographic signal. The PEC rate was determined by measuring the H_2_ created at 30 min intervals (for a total of 3 h). The photocathode's HER was repeated three times and averaged.


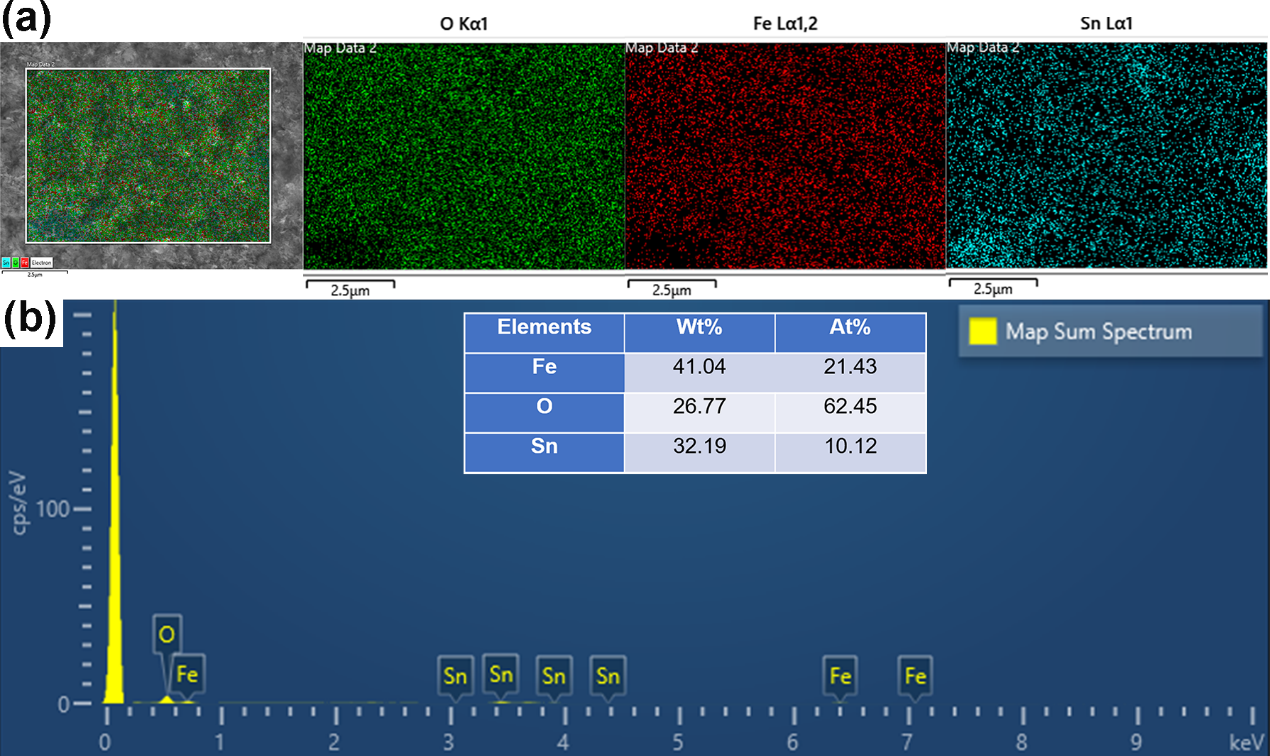


**Figure S1.** EDS mapping (a) and the corresponding EDS spectrum (b) of the FeOOH thin film.


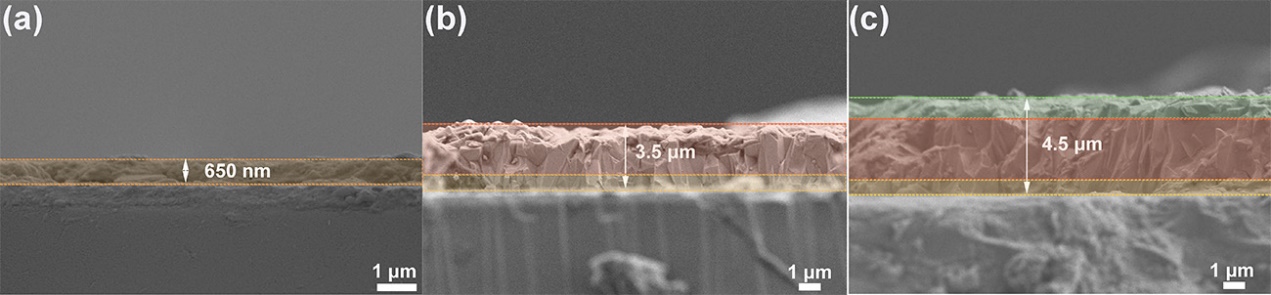


**Figure S2.** Cross-sectional SEM images of FeOOH (a), FeOOH/Cu_2_O (b) and FeOOH/Cu_2_O/ZnO (c).

_
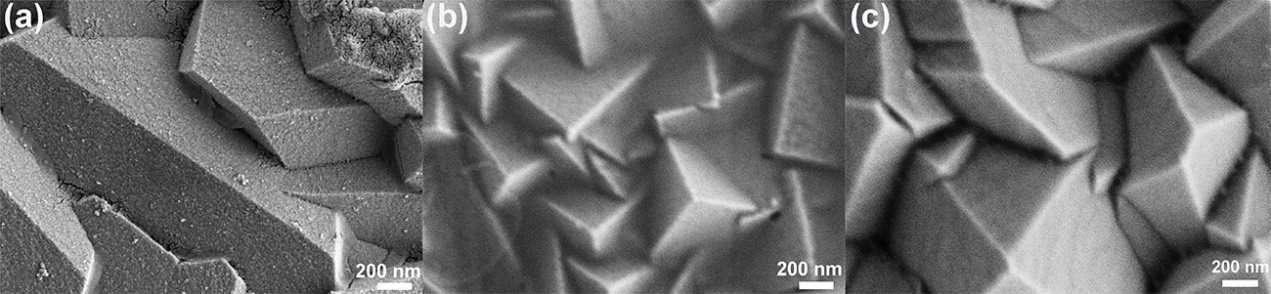
_

**Figure S3.** Top-view SEM images of Cu_2_O prepared at different temperature: 55 °C (a), 60 °C (b) and 65 °C (c).

_
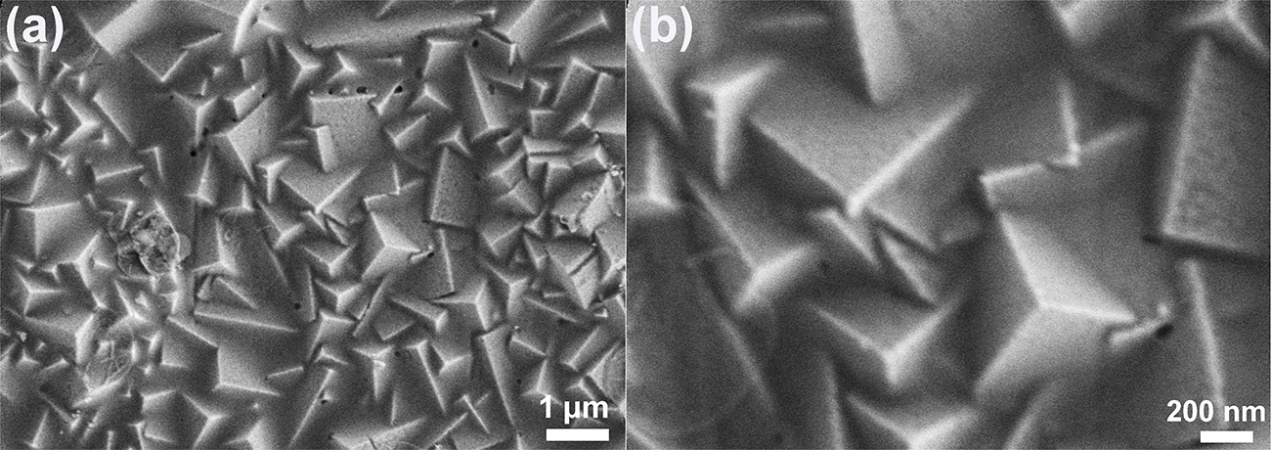
_

**Figure S4.** Low-resolution and high-resolution SEM images of Cu_2_O prepared at 60 °C (a, b).


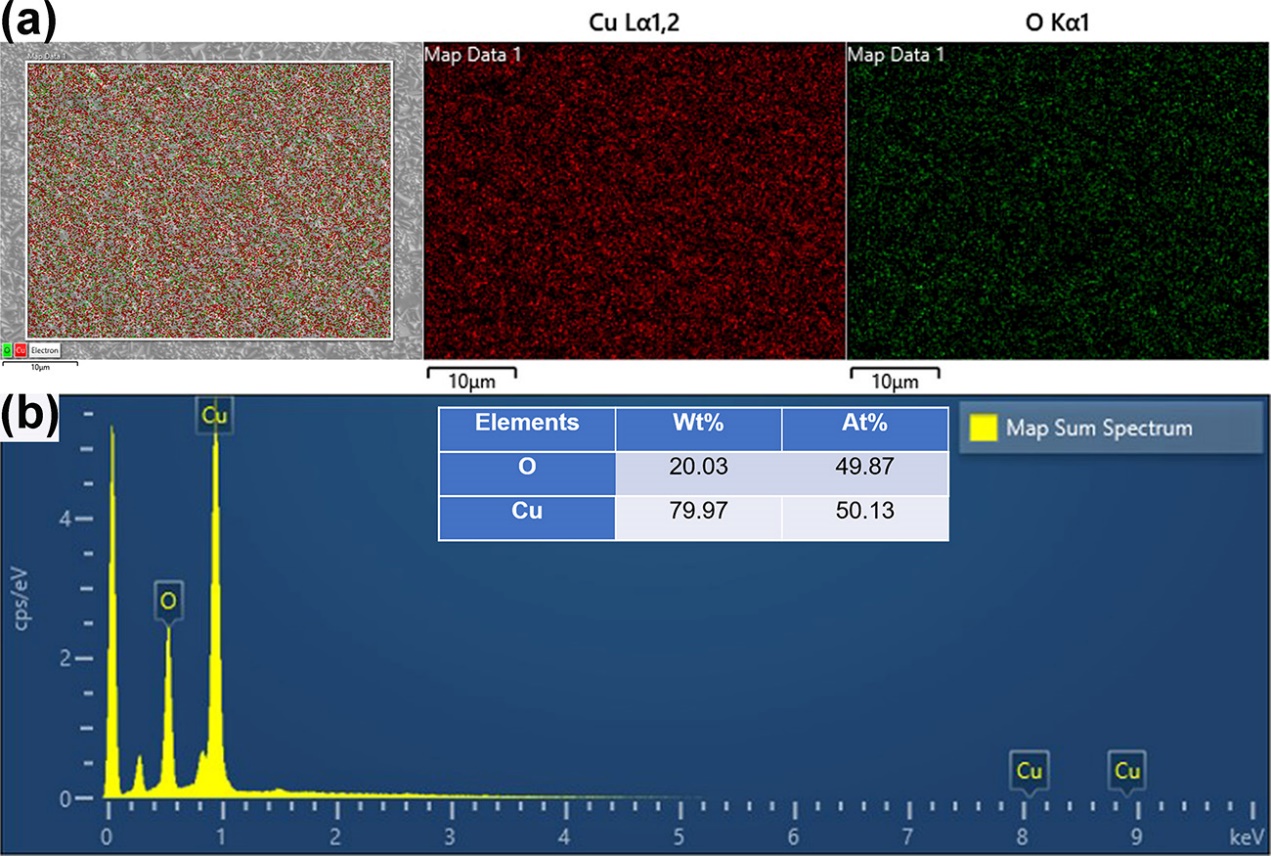


**Figure S5.** EDS mapping (a) and the corresponding EDS spectrum (b) of the Cu_2_O thin film prepared at 60 °C.


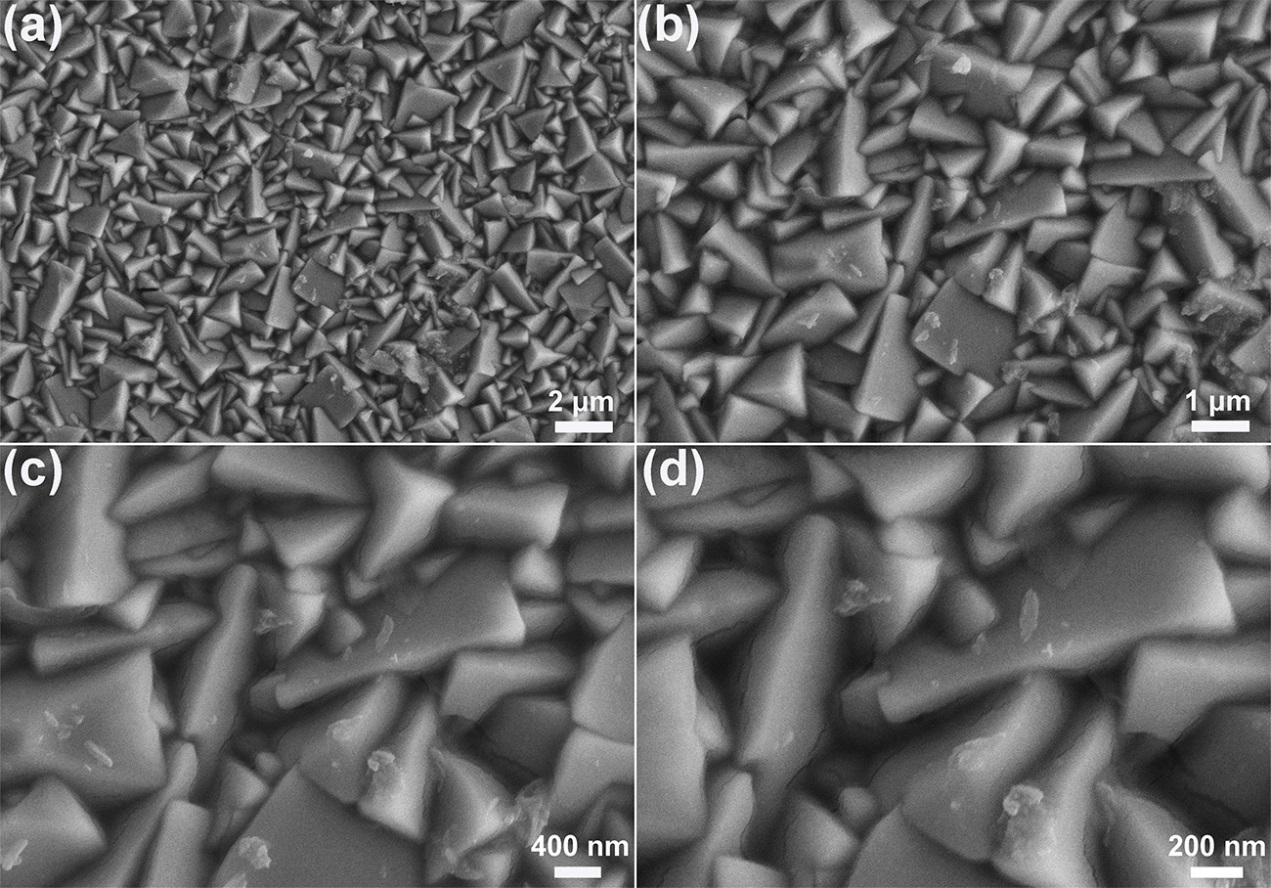


**Figure S6.** Low-resolution (a, b) and high-resolution (c, d) SEM images of FeOOH/Cu_2_O.


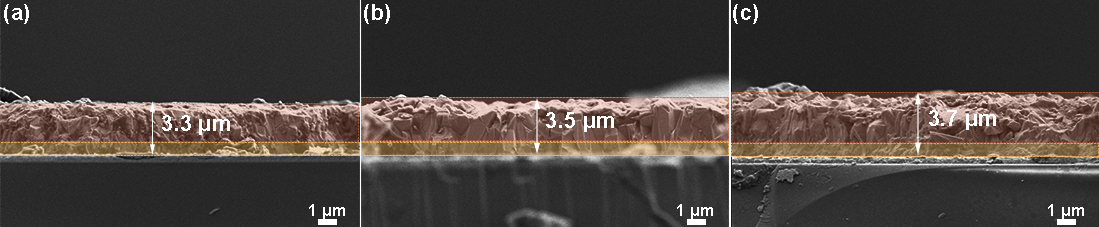


**Figure S7.** Cross-sectional SEM images of FeOOH/Cu_2_O prepared at different electrodeposition time: 10 min (a), 15 min (b) and 20 min (c).


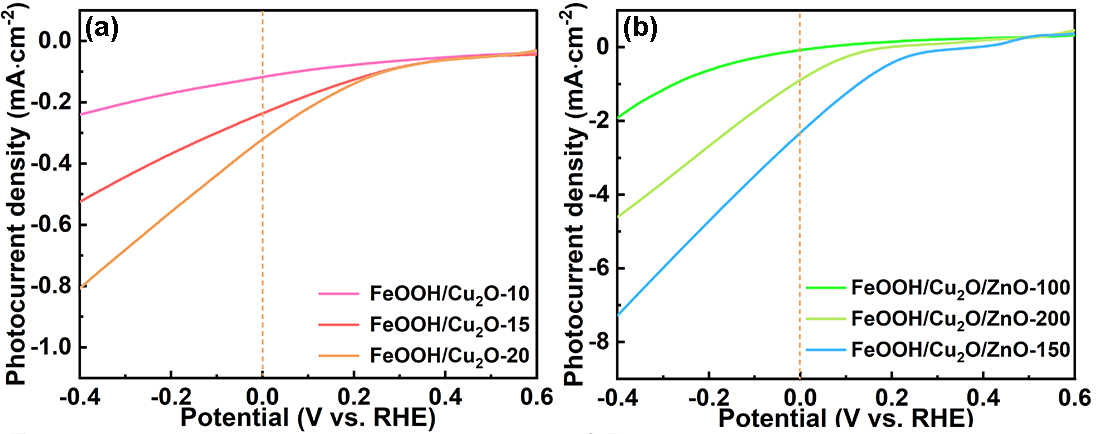


**Figure S8.** LSV plots of FeOOH/Cu_2_O-10, FeOOH/Cu_2_O-15 and FeOOH/Cu_2_O-20 (a), FeOOH/Cu_2_O/ZnO-100, FeOOH/Cu_2_O/ZnO-150 and FeOOH/Cu_2_O/ZnO-200 (b).


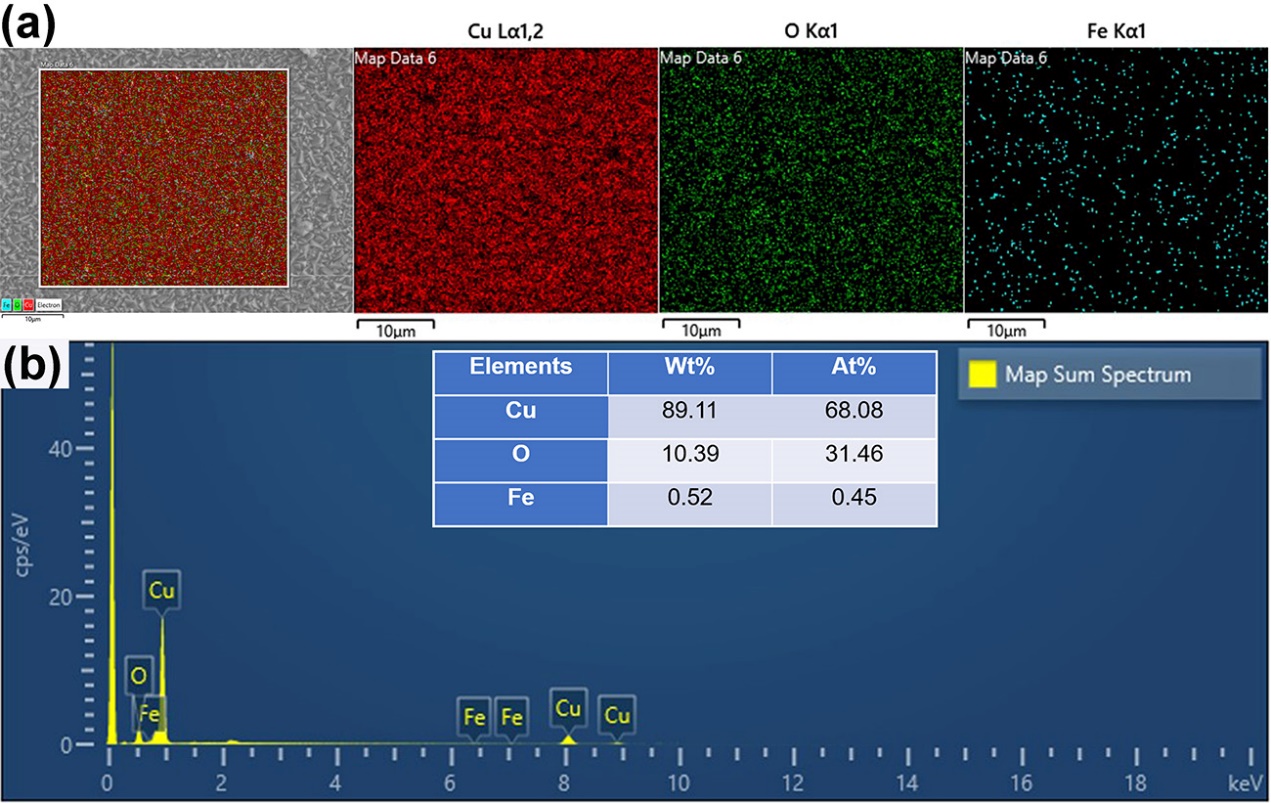


**Figure S9.** EDS mapping (a) and the corresponding EDS spectrum (b) of the FeOOH/Cu_2_O photocathode.


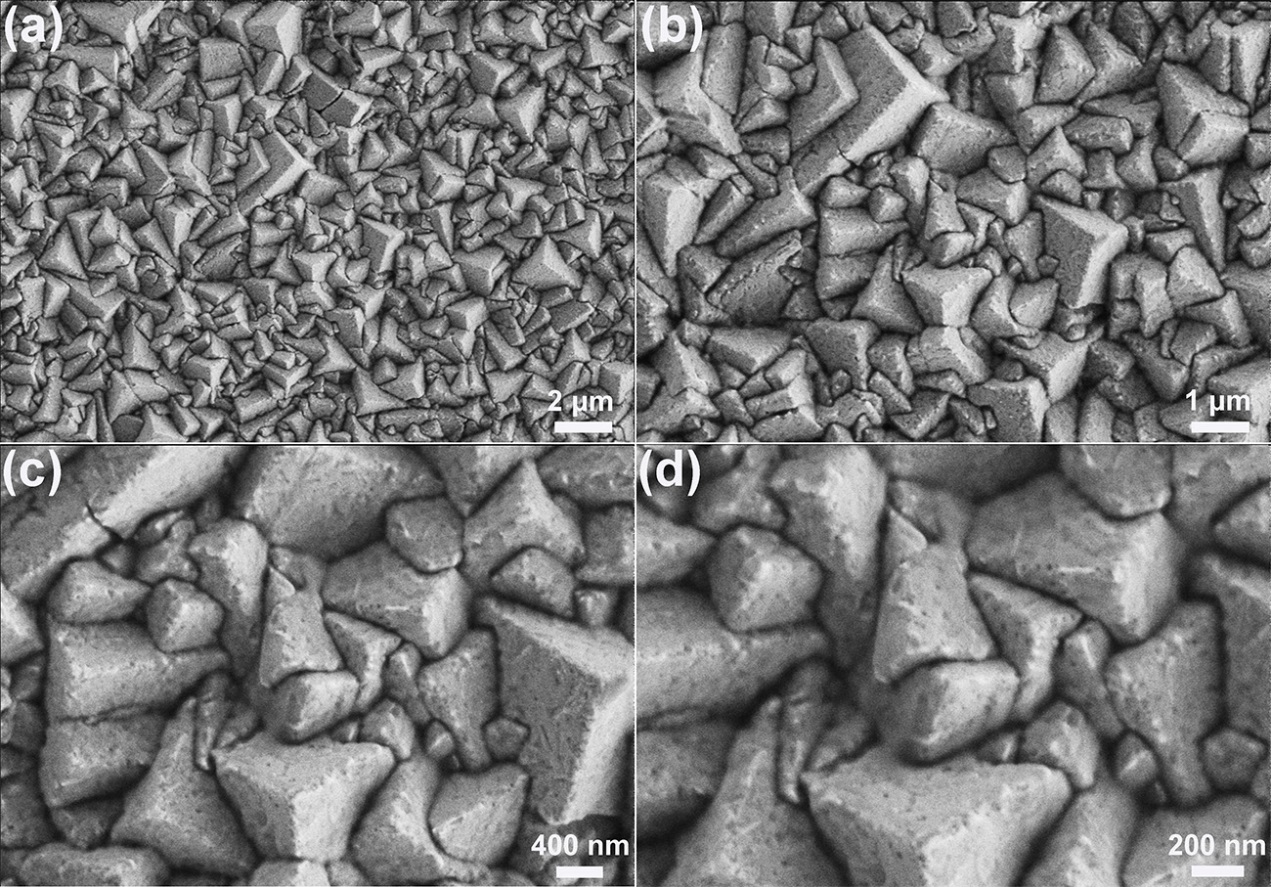


**Figure S10.** Low-resolution (a, b) and high-resolution (c, d) SEM images of Cu_2_O/ZnO.


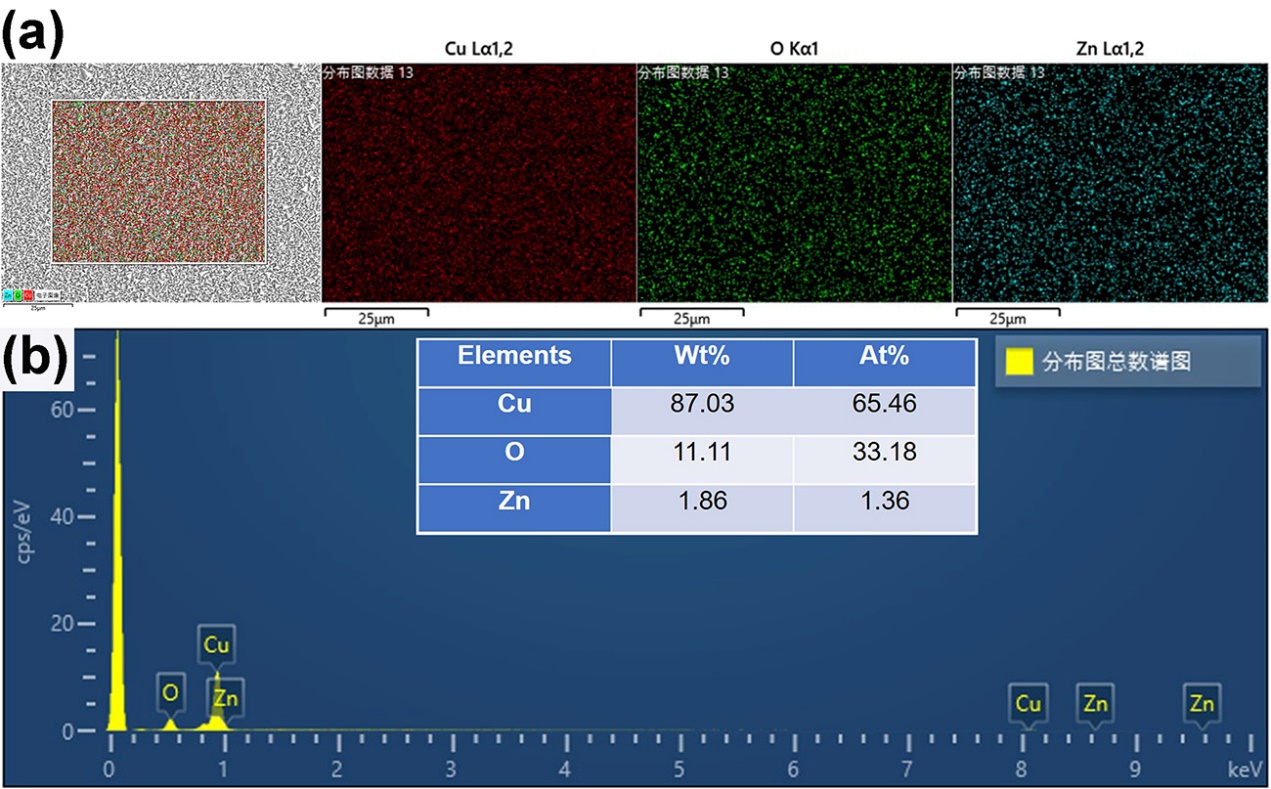


**Figure S11.** EDS mapping (a) and the corresponding EDS spectrum (b) of the Cu_2_O/ZnO photocathode.


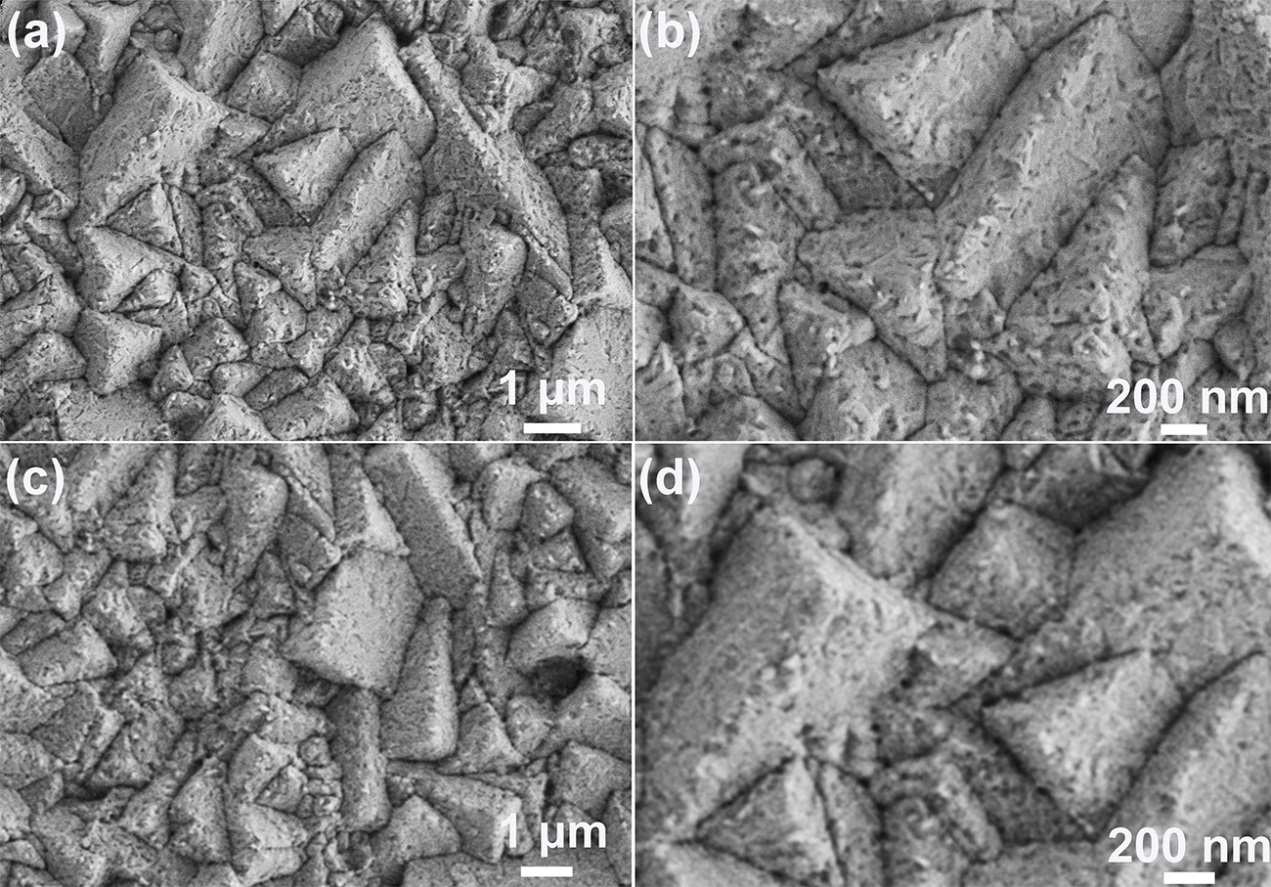


**Figure S12.** Top-view SEM images before PEC measurements (a, b) and high-resolution SEM images after PEC measurements (c, d) of FeOOH/Cu_2_O/ZnO.


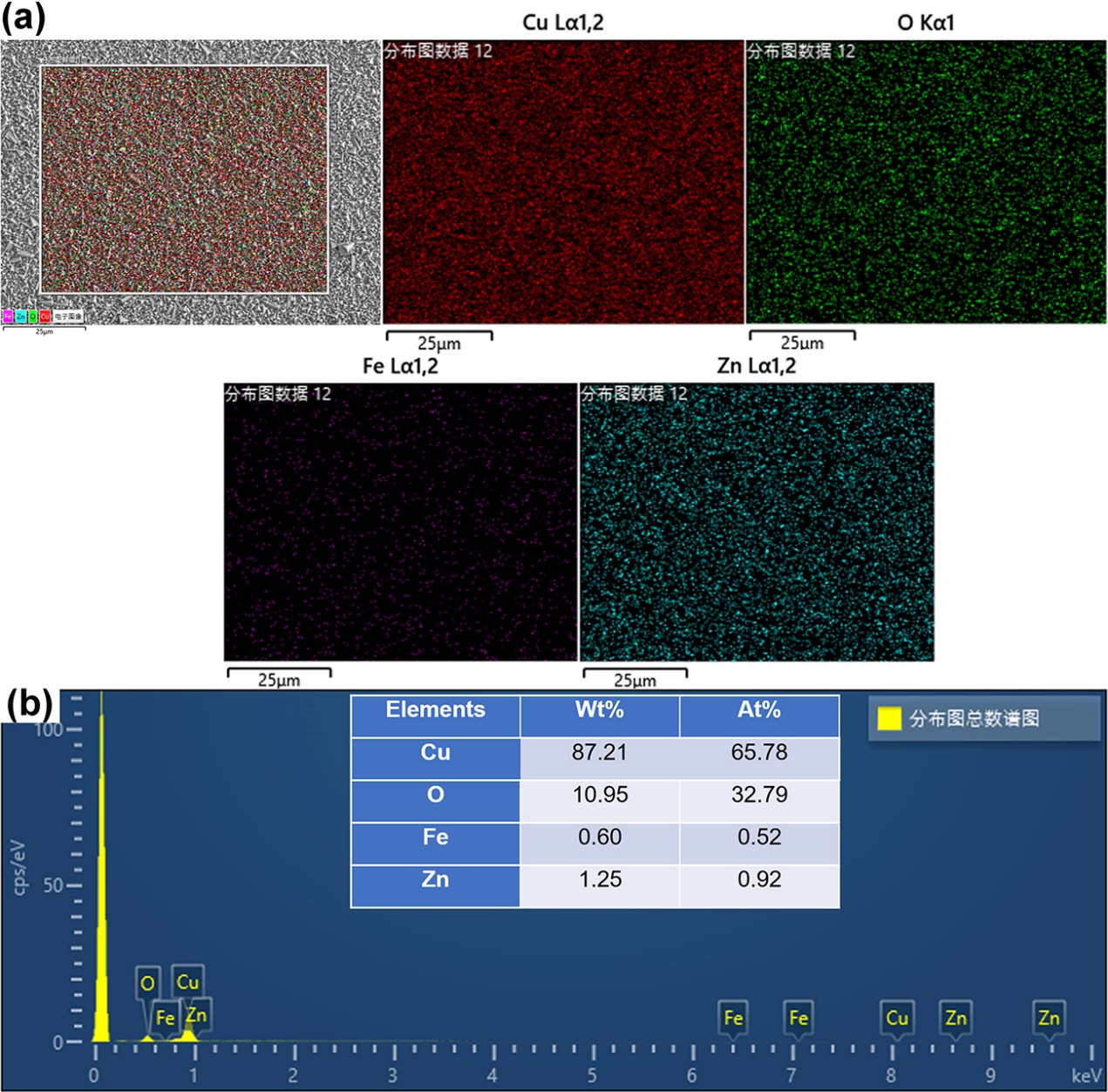


**Figure S13.** EDS mapping (a) and the corresponding EDS spectrum (b) of the FeOOH/Cu_2_O/ZnO photocathode.


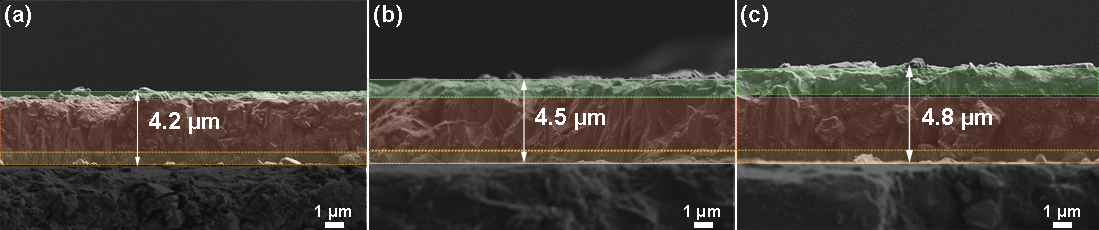


**Figure S14.** Cross-sectional SEM images of FeOOH/Cu_2_O/ZnO prepared at different electrodeposition time: 100 s (a), 150 s (b) and 200 s (c).


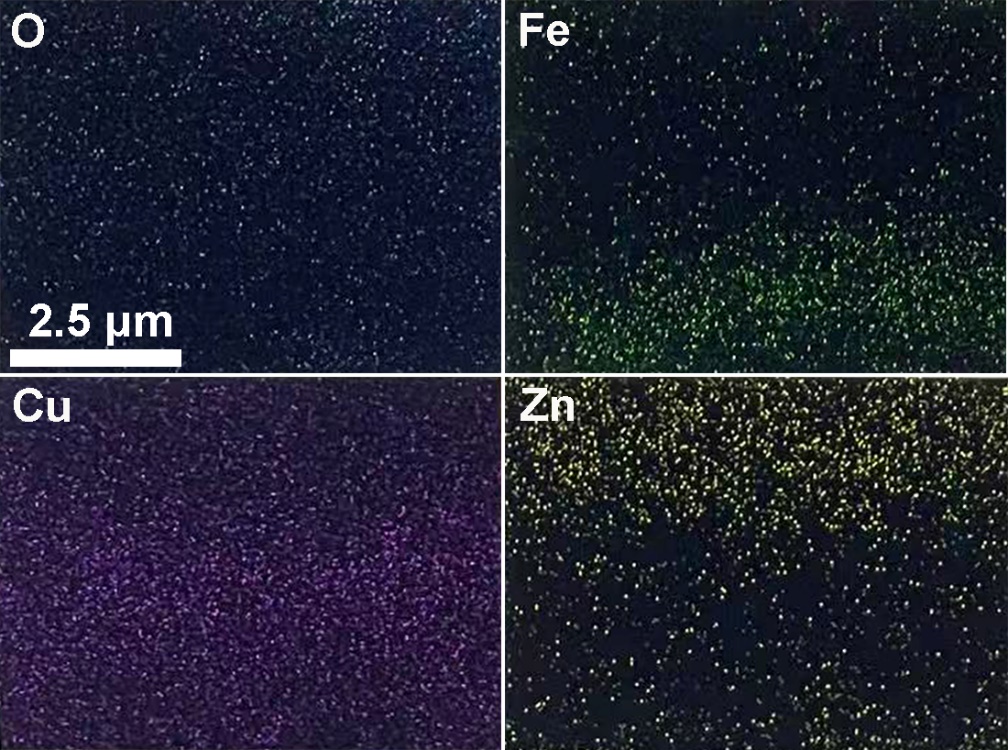


**Figure S15.** EDS mapping of the cross-sectional SEM images of the FeOOH/Cu_2_O/ZnO photocathode.


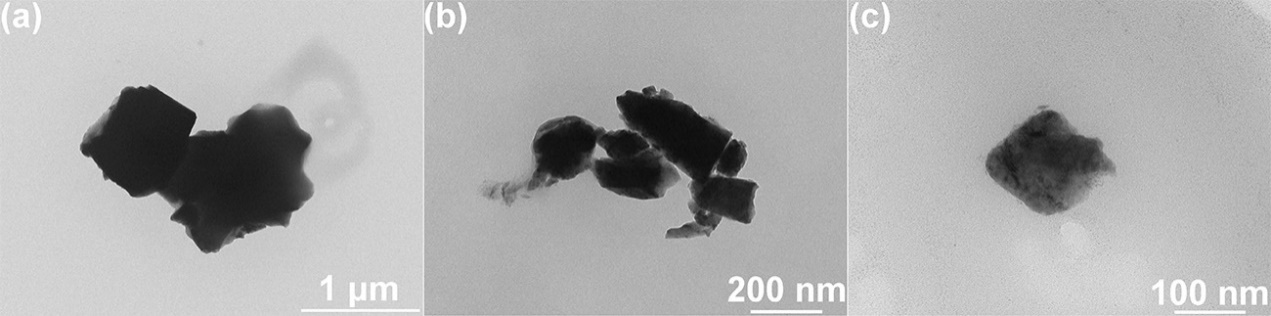


**Figure S16.** TEM images of Cu_2_O prepared at 60 °C.


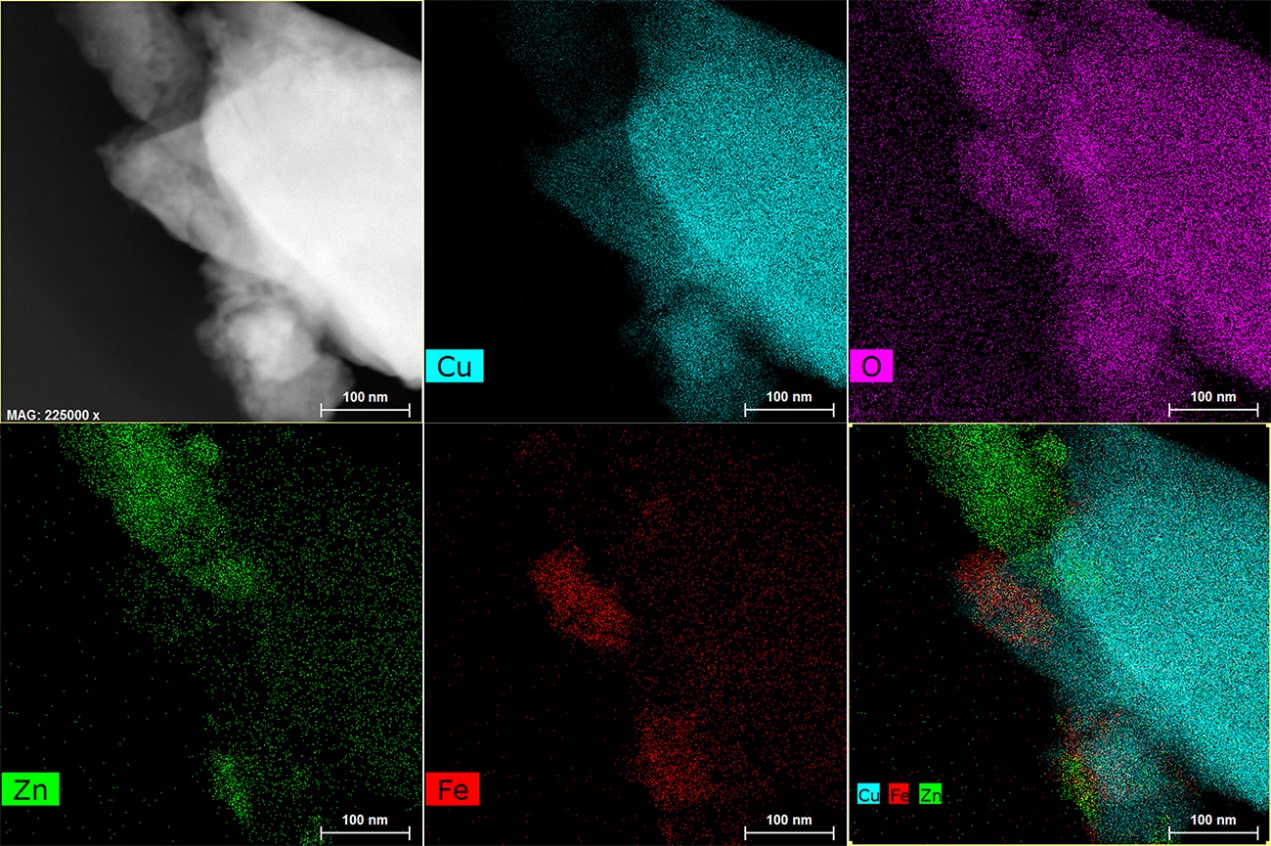


**Figure S17.** EDS mapping image of FeOOH/Cu_2_O/ZnO.


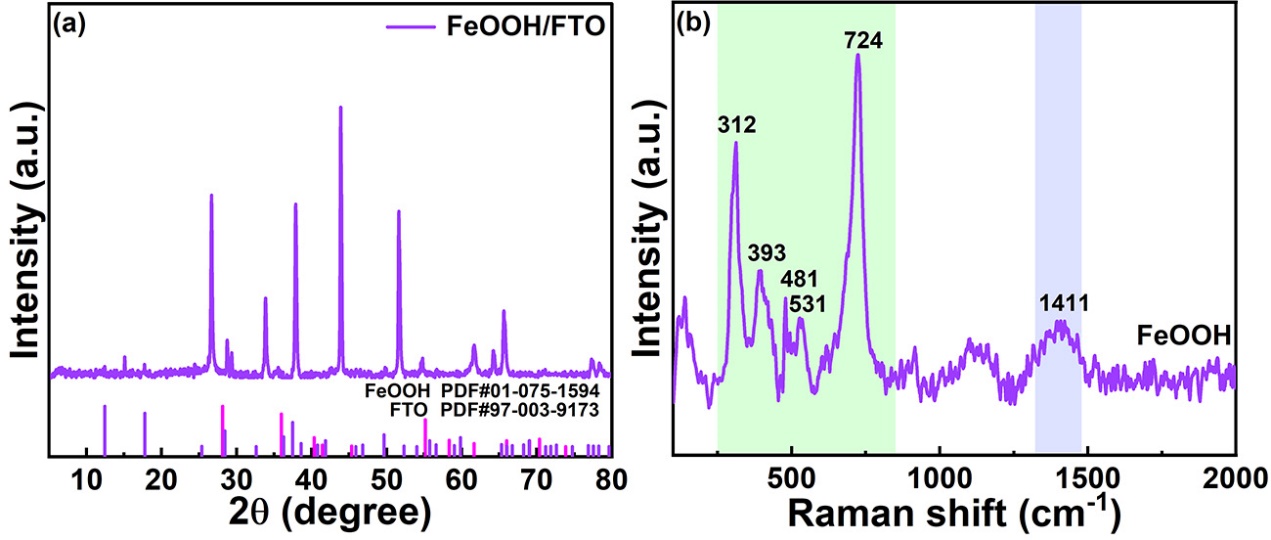


**Figure S18.** XRD patterns (a) and Raman spectrum (b) of FeOOH/FTO.


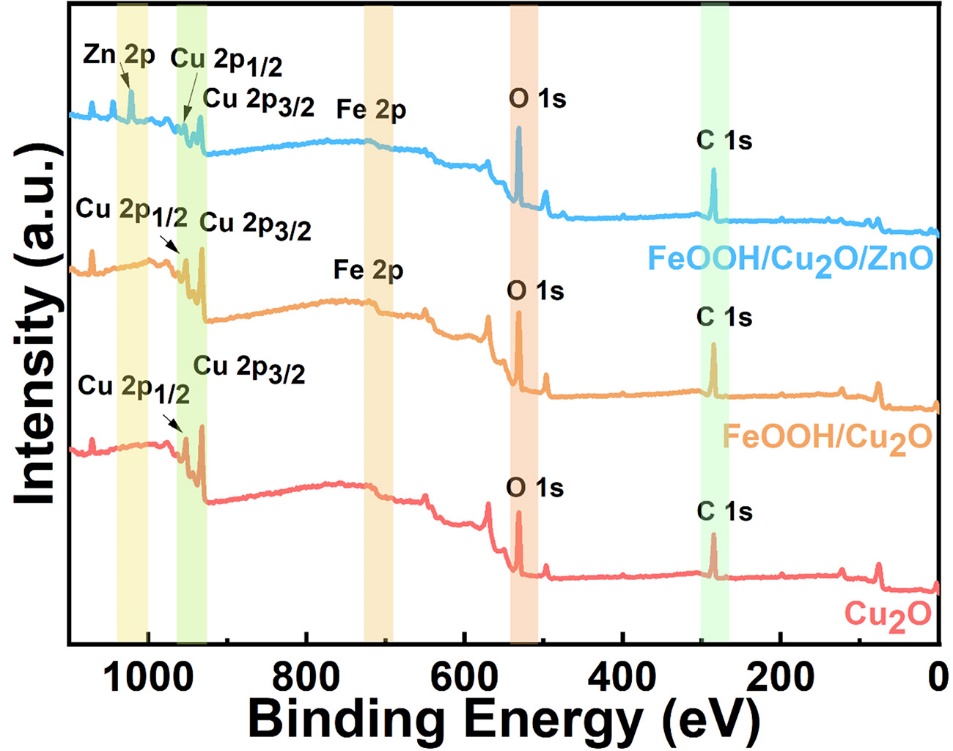


**Figure S19.** XPS survey spectra of Cu_2_O, FeOOH/Cu_2_O and FeOOH/Cu_2_O/ZnO.


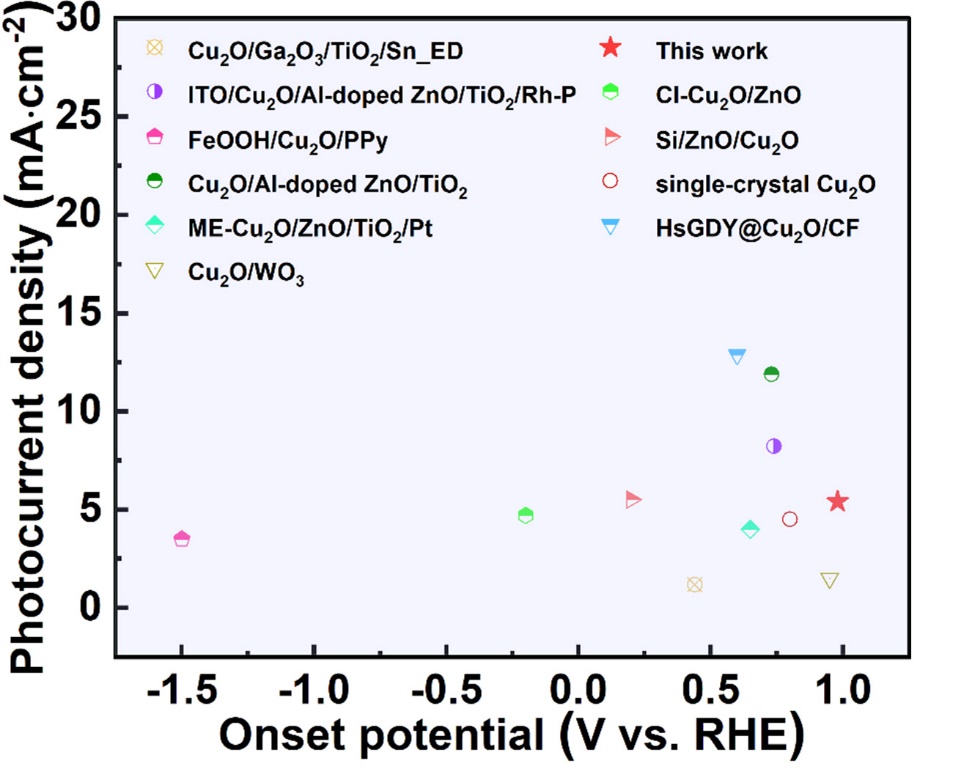


**Figure S20.** Comparison of our work with representative literature reports.


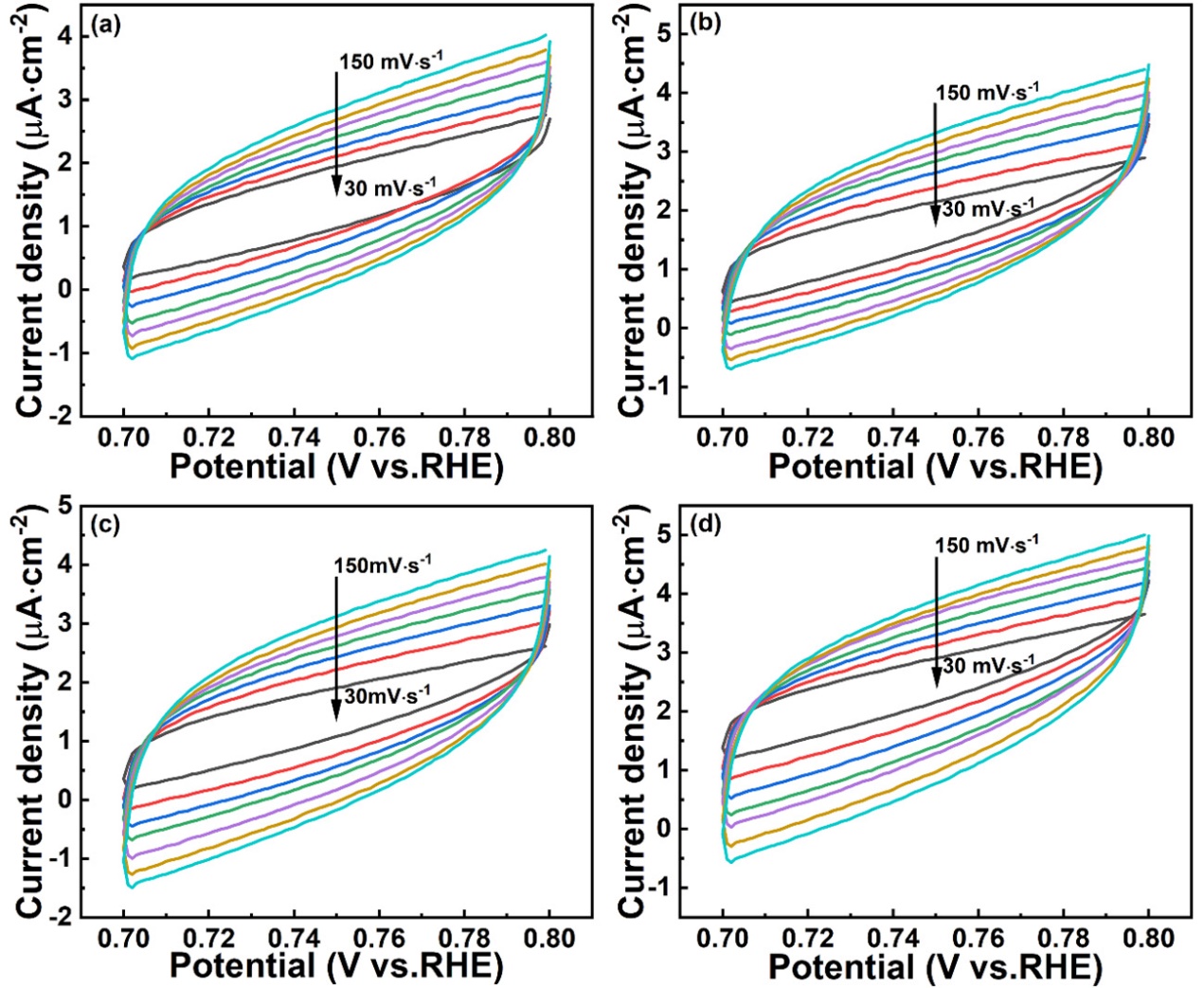


**Figure S21.** CV curves of Cu_2_O (a), FeOOH/Cu_2_O (b), Cu_2_O/ZnO (c) and FeOOH/Cu_2_O/ZnO (d).


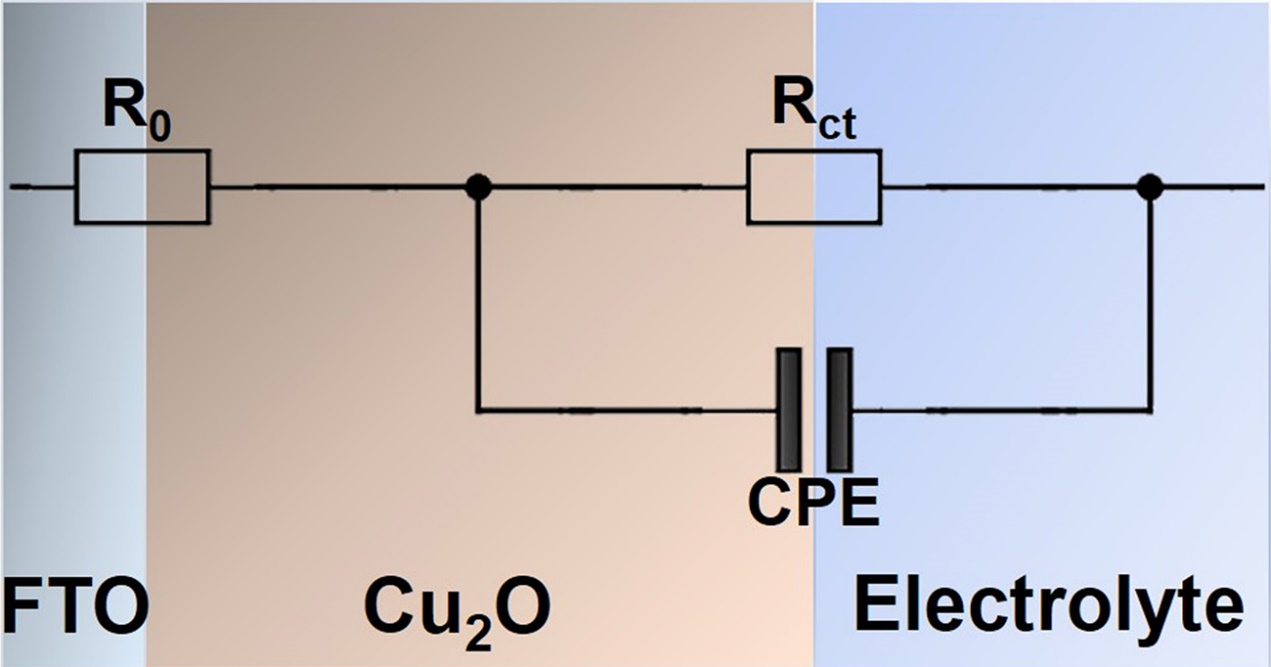


**Figure S22.** The fitting equivalent circuit diagram.


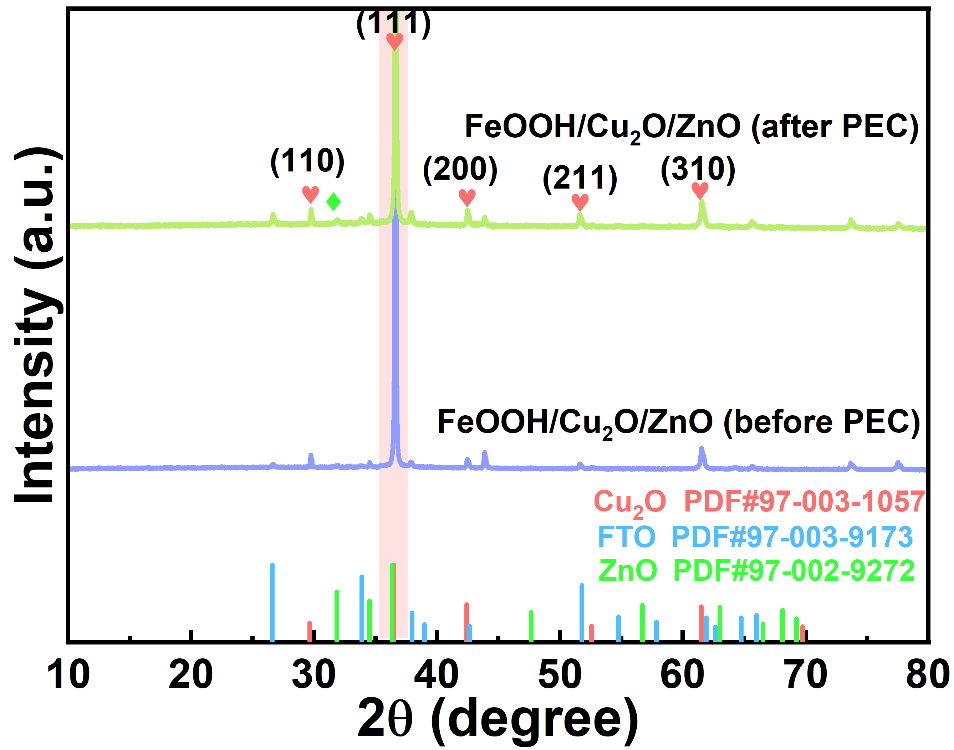


**Figure S23.** XRD patterns of FeOOH/Cu_2_O/ZnO before and after long-term PEC measurements.


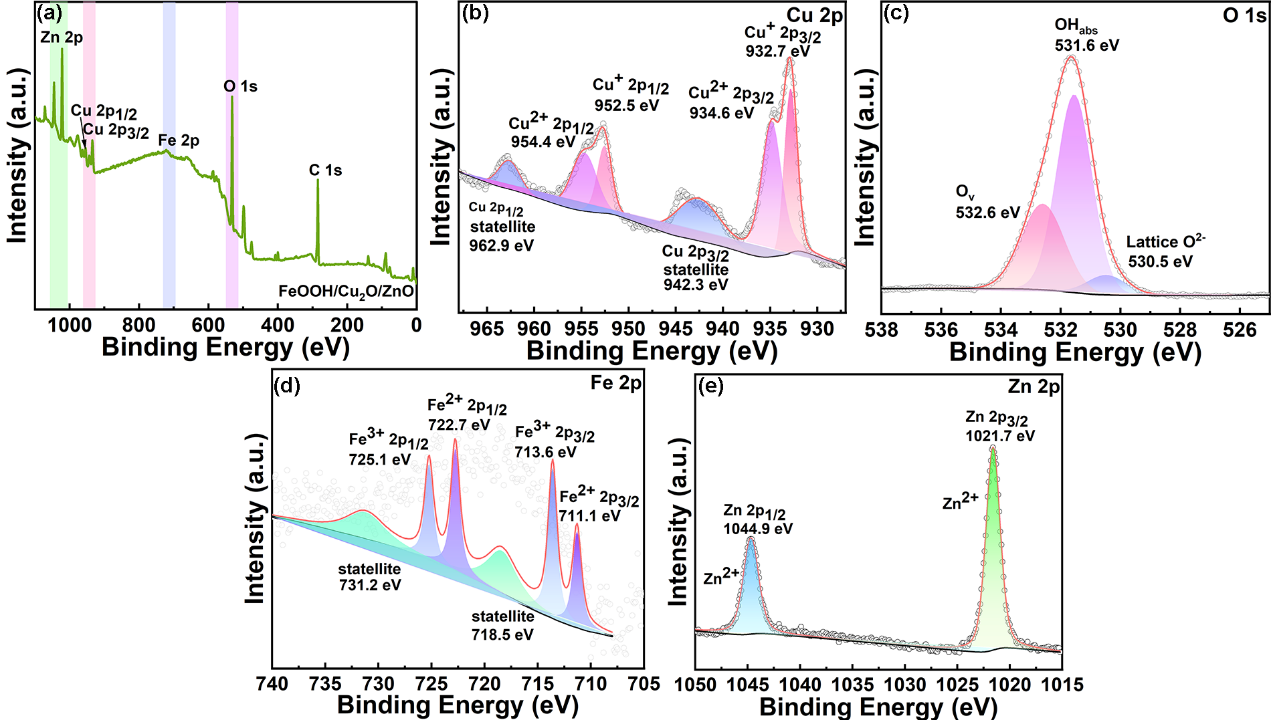


**Figure S24.** Survey XPS spectra (a), Cu 2p (b), O 1s (c), Fe 2p (d), and Zn 2p (e) high-resolution XPS spectra of FeOOH/Cu_2_O/ZnO after long-term PEC test.


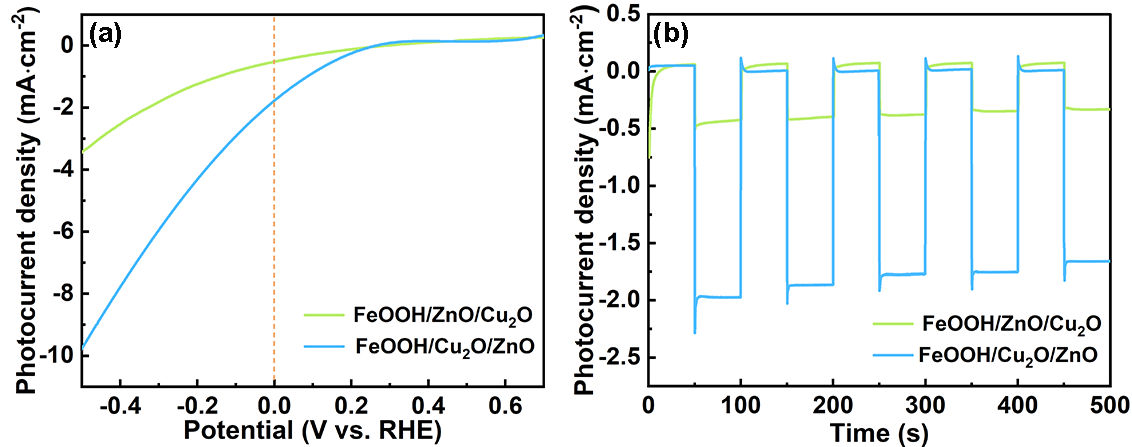


**Figure S25.** LSV plots (a) and transient photocurrent responses (b) of FeOOH/ZnO /Cu_2_O and FeOOH/Cu_2_O/ZnO.


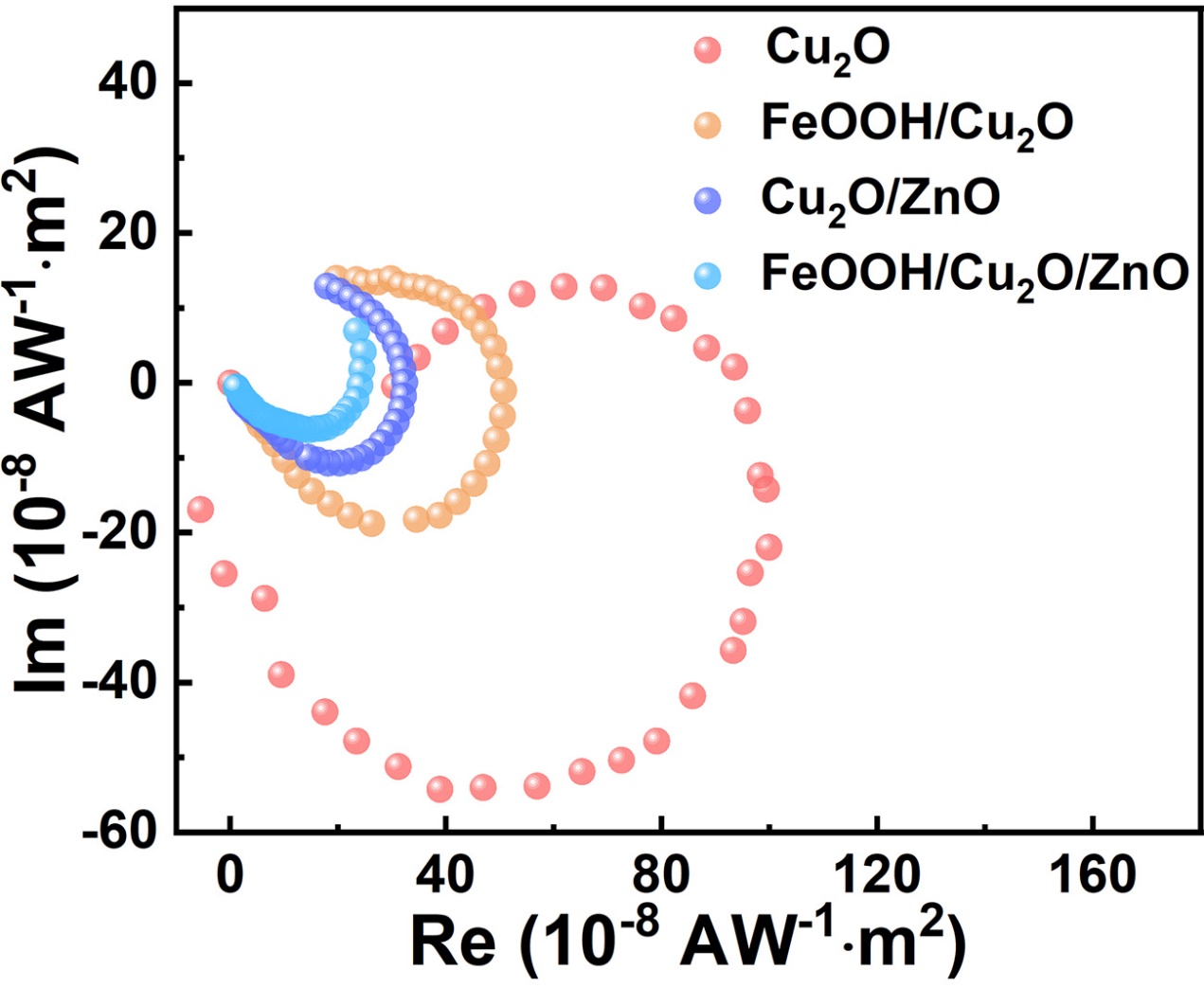


**Figure S26.** IMPS for Cu_2_O, FeOOH/Cu_2_O, Cu_2_O/ZnO, and FeOOH/Cu_2_O/ZnO photocathodes tested under one sun illumination in 0.5 M Na_2_SO_4_ electrolyte.


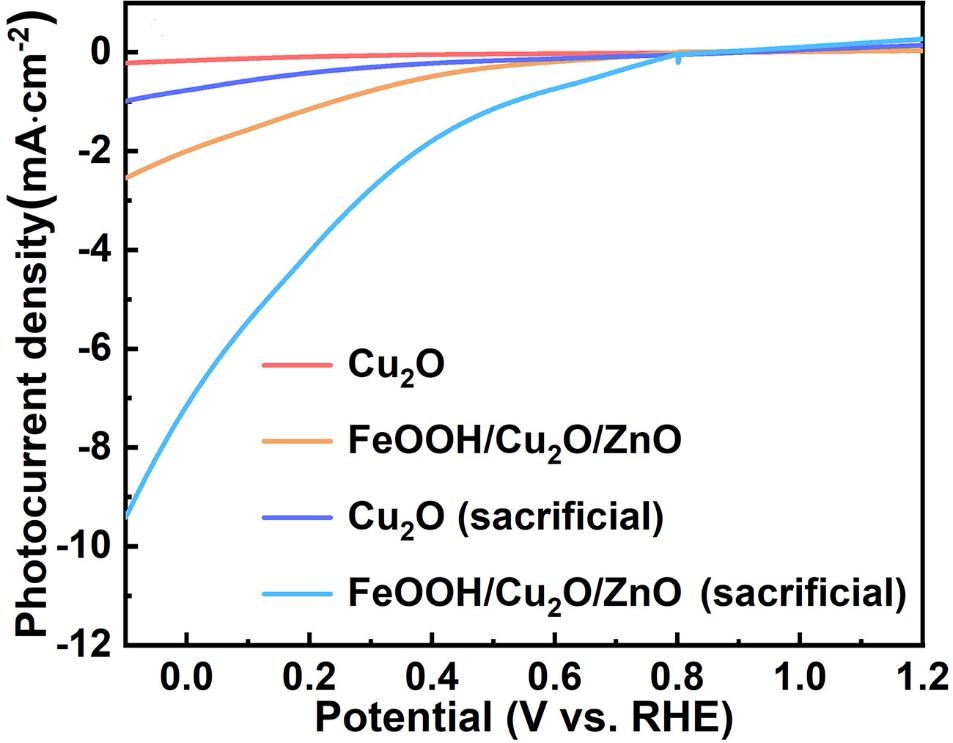


**Figure S27.** LSV of FeOOH/Cu_2_O/ZnO and Cu_2_O photocathodes measured 0.5 M Na_2_S_2_O_8_ electrolyte, compared with the LSV of FeOOH/Cu_2_O/ZnO and Cu_2_O photocathodes measured in 0.5 M Na_2_SO_4_ electrolyte under AM 1.5 G chopped light illumination.


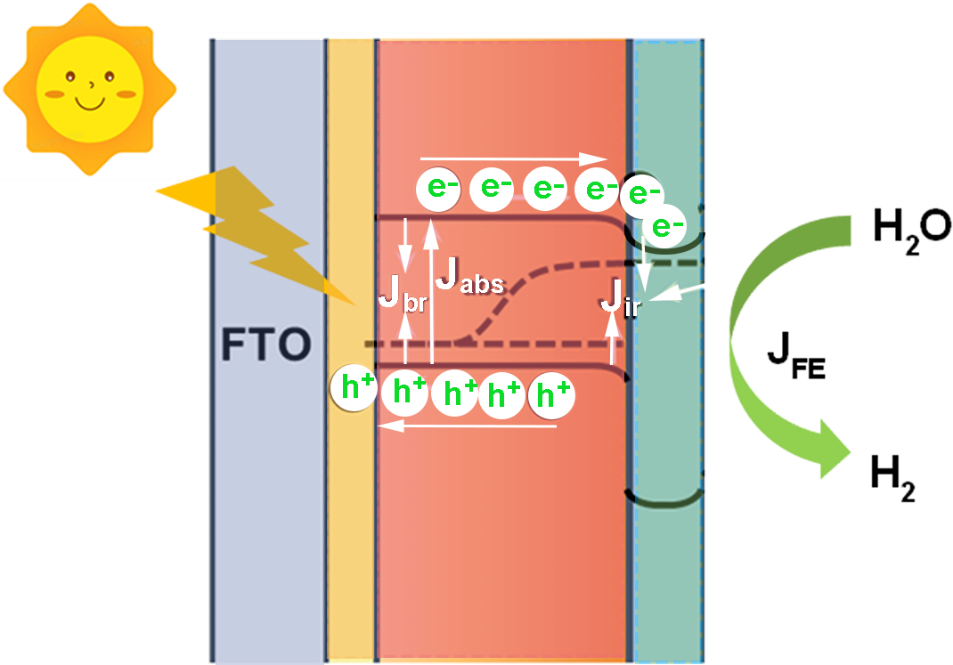


**Figure S28.** Illustration the FeOOH/Cu_2_O/ZnO photocathode with the charge photogeneration, transfer and recombination process during the PEC water reduction.


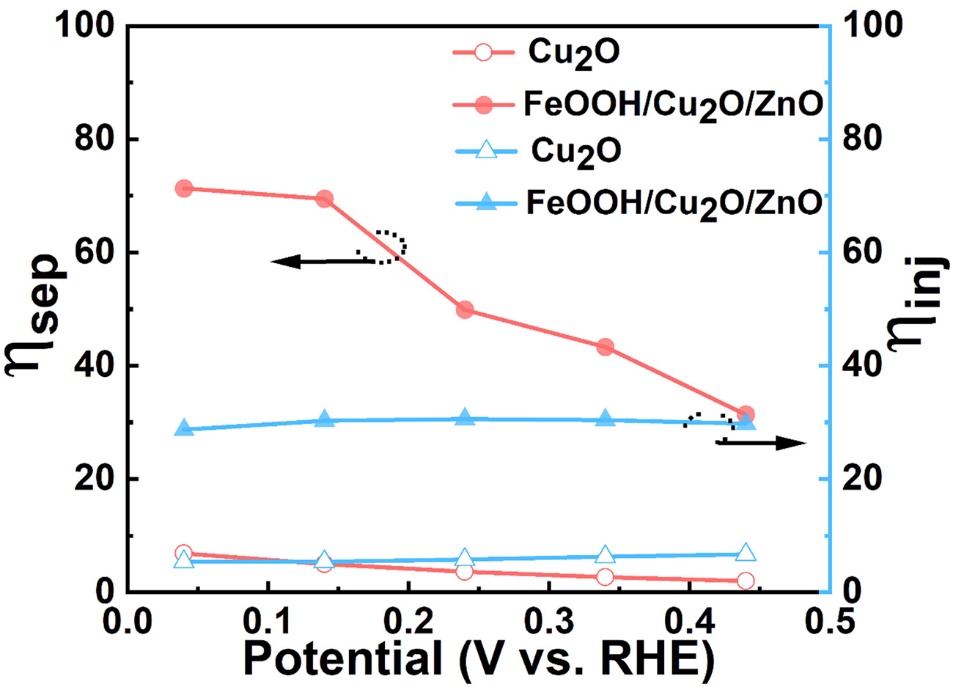


**Figure S29.** *η*_sep_ and *η*_inj_ of Cu_2_O and FeOOH/Cu_2_O/ZnO photocathodes calculated with LSV measured in different electrolytes.


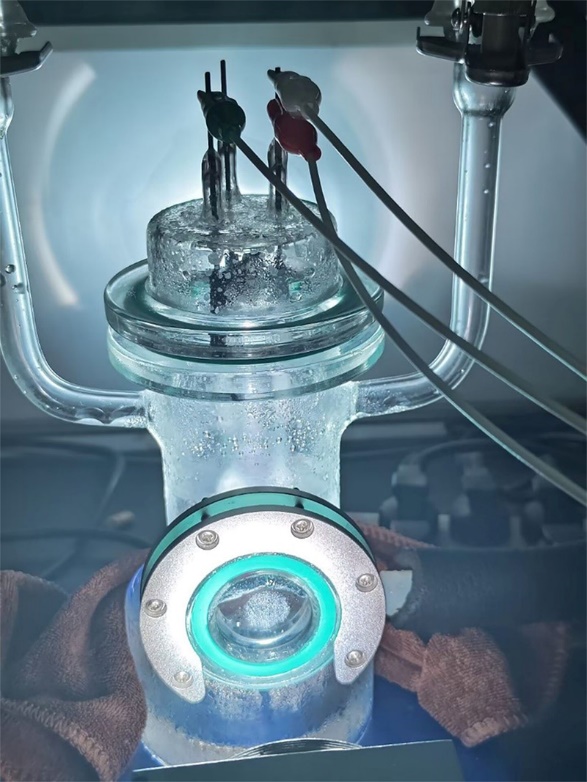


**Figure S30.** Schematic illustration of PEC water reduction device with concentrated solar light illumination.


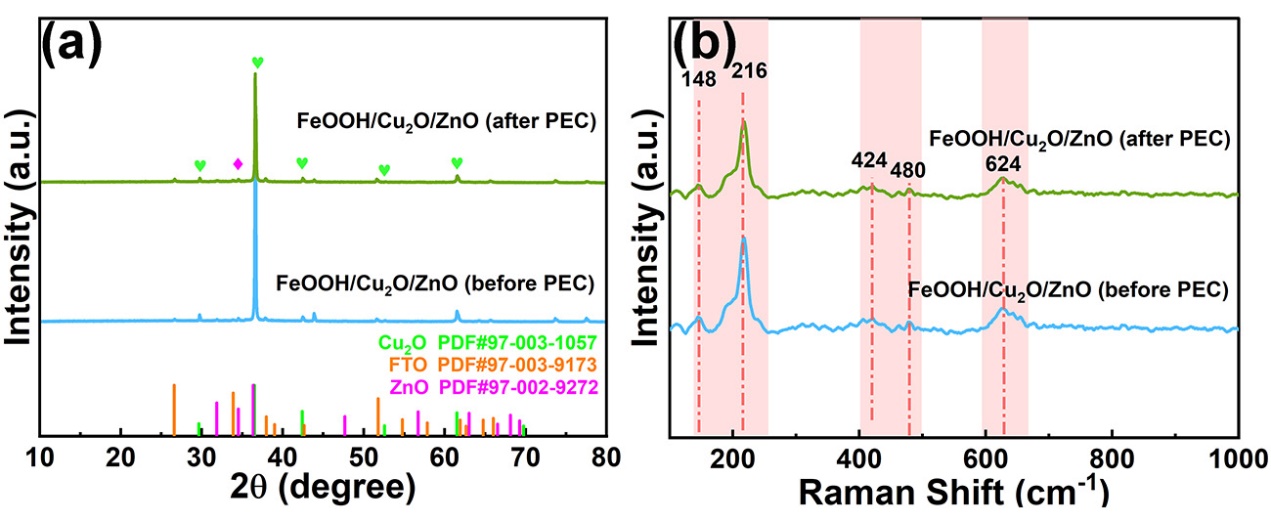


**Figure S31.** XRD patterns (a) and Raman spectra (b) of FeOOH/Cu_2_O/ZnO before and after PEC measurements.


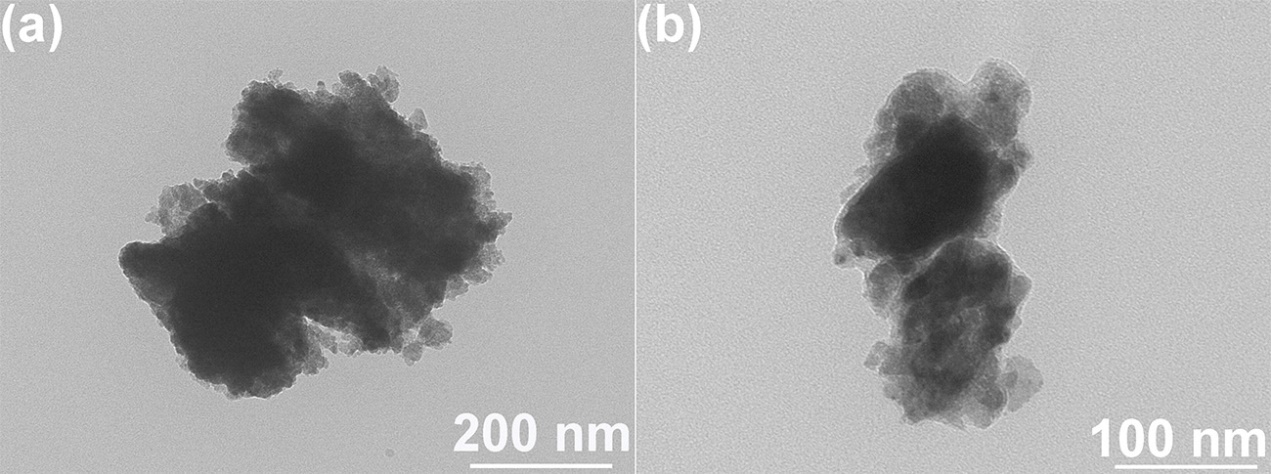


**Figure S32.** TEM images of FeOOH/Cu_2_O/ZnO after PEC measurements.


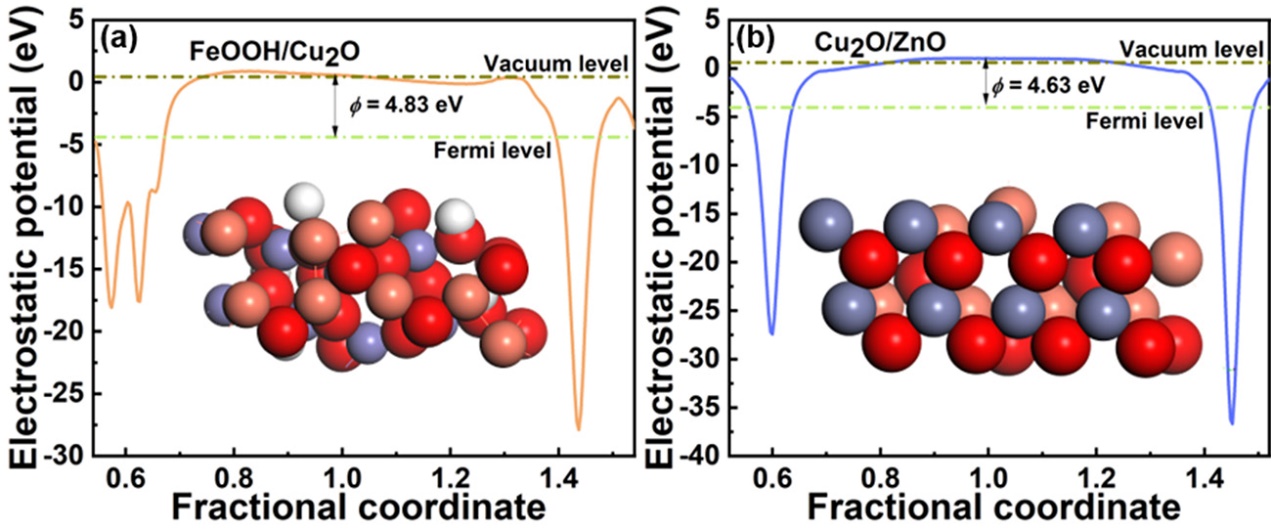


**Figure S33.** The electrostatic potential distribution of FeOOH/Cu_2_O (a) and Cu_2_O/ZnO (b) along Z direction.


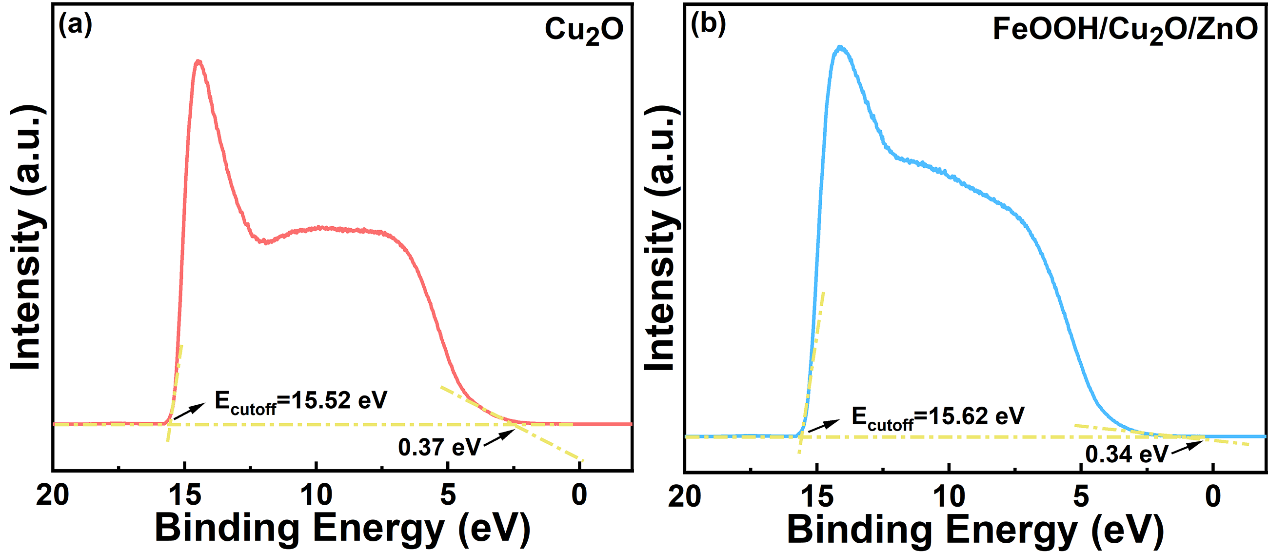


**Figure S34.** UPS spectra of Cu_2_O (a) and FeOOH/Cu_2_O/ZnO (b).


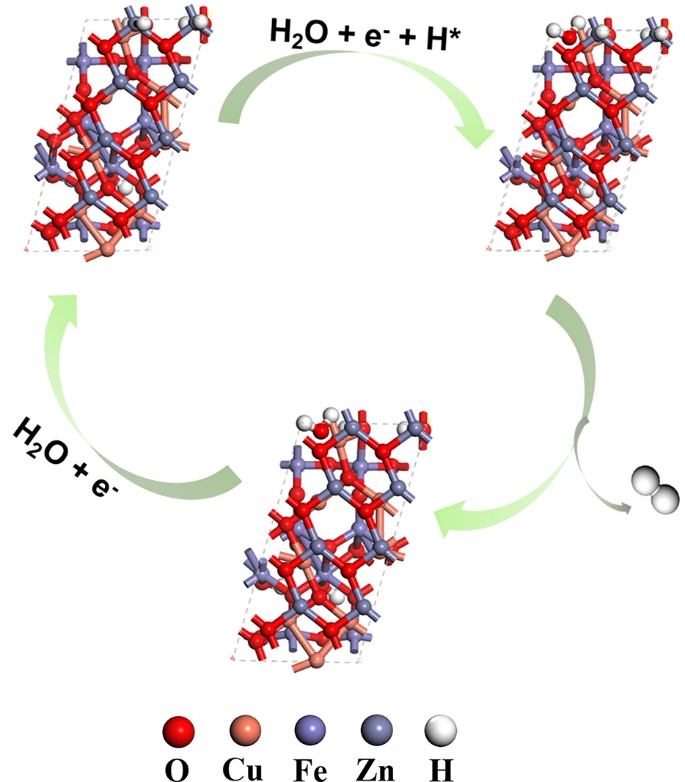


**Figure S35.** Reaction mechanism diagram of FeOOH/Cu_2_O/ZnO photocathodes.


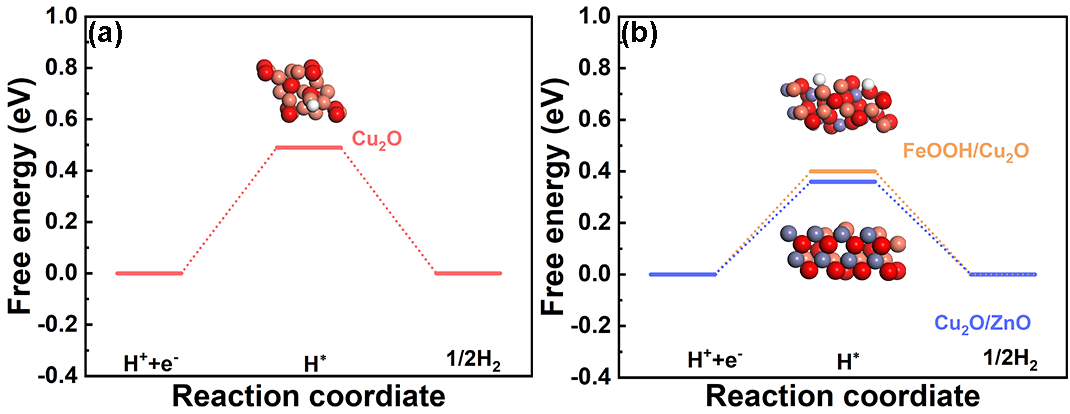


**Figure S36.** HER reaction steps of Cu_2_O photocathodes (a) and FeOOH/Cu_2_O and Cu_2_O/ZnO photocathodes (b).


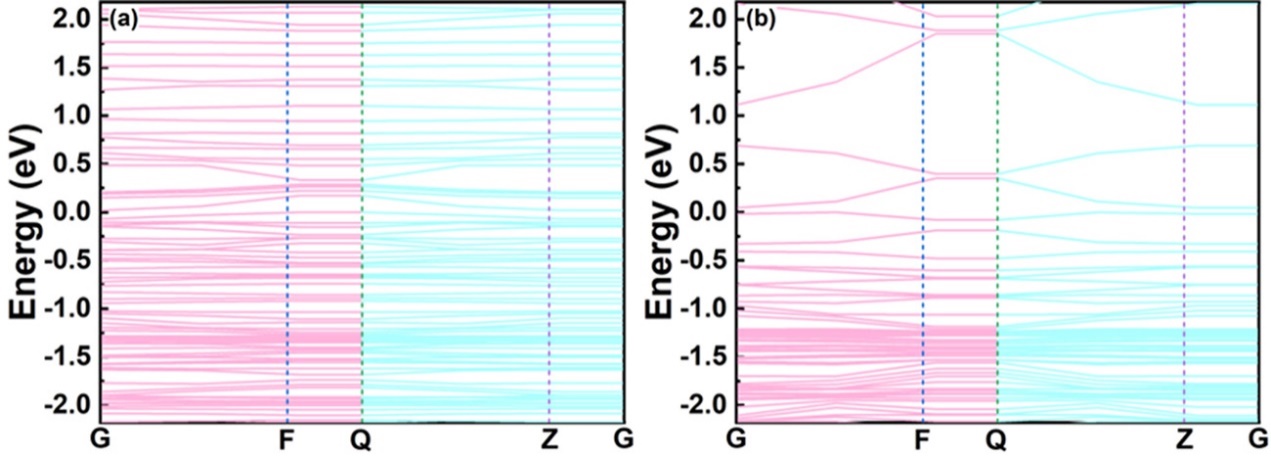


**Figure S37.** The energy band structure of FeOOH/Cu_2_O (a) and Cu_2_O/ZnO (b).


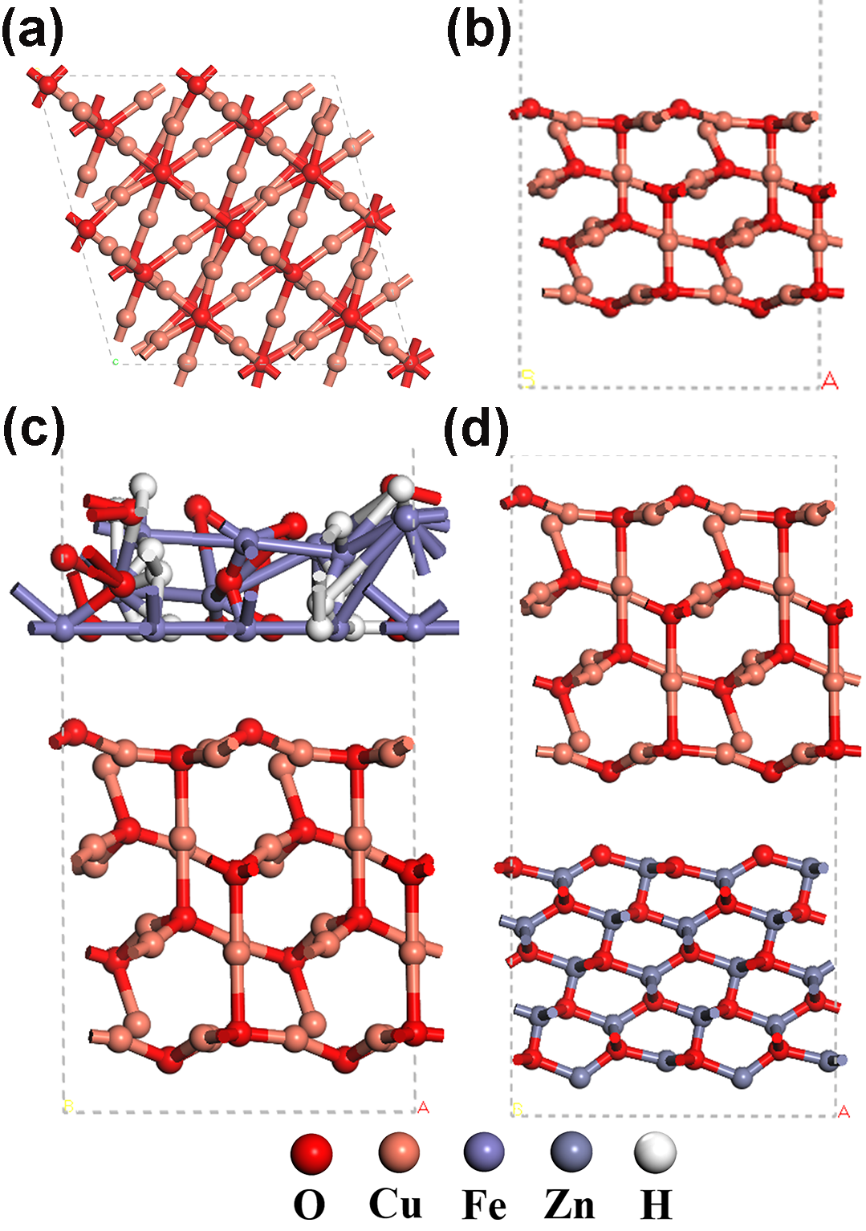


**Figure S38.** The optimal theoretical structure of Cu_2_O (a, b), FeOOH/Cu_2_O (c) and Cu_2_O/ZnO (d).


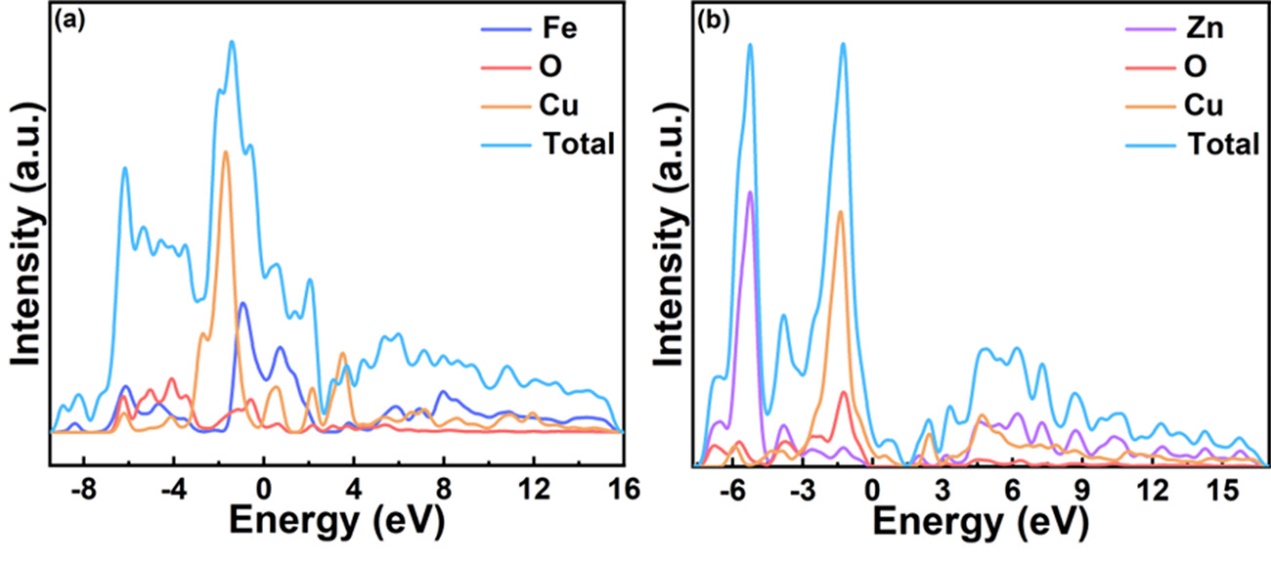


**Figure S39.** The density of states of FeOOH/Cu_2_O (a) and Cu_2_O/ZnO (b).


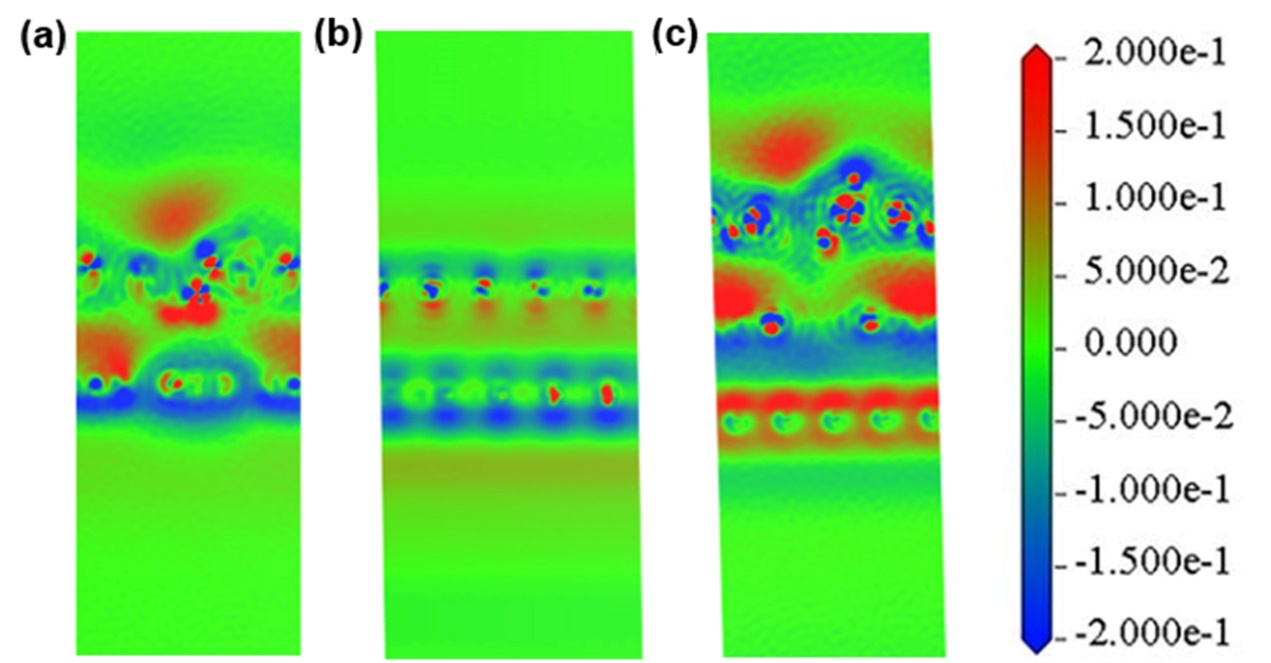


**Figure S40.** Electron density difference maps of FeOOH/Cu_2_O (a), Cu_2_O/ZnO (b) and FeOOH/Cu_2_O/ZnO (c).


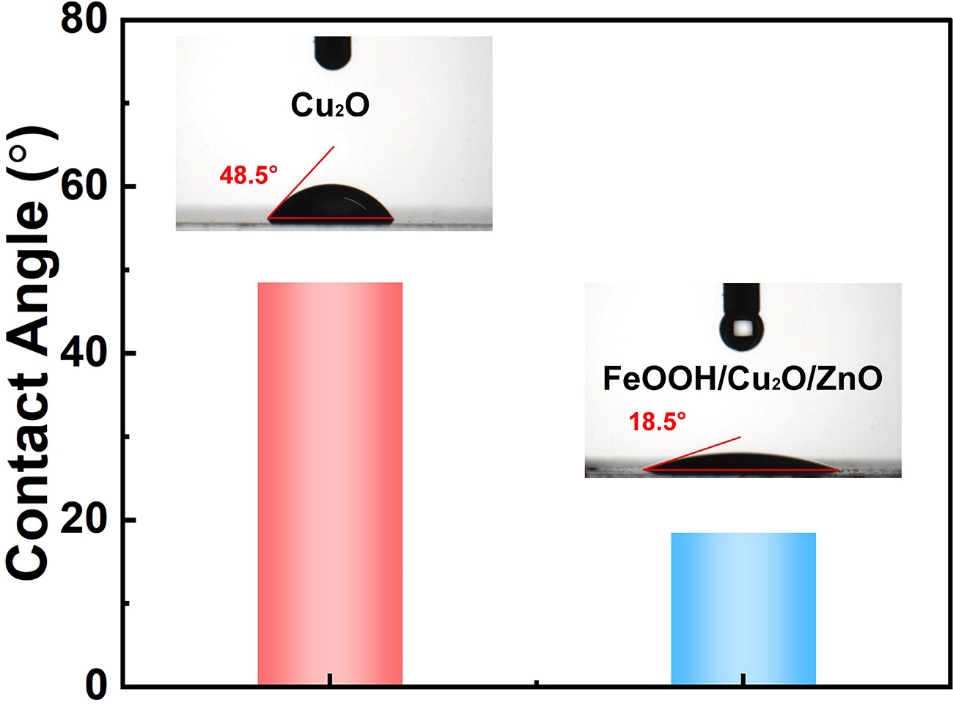


**Figure S41.** Views of surface contact measurements for the photocathodes.


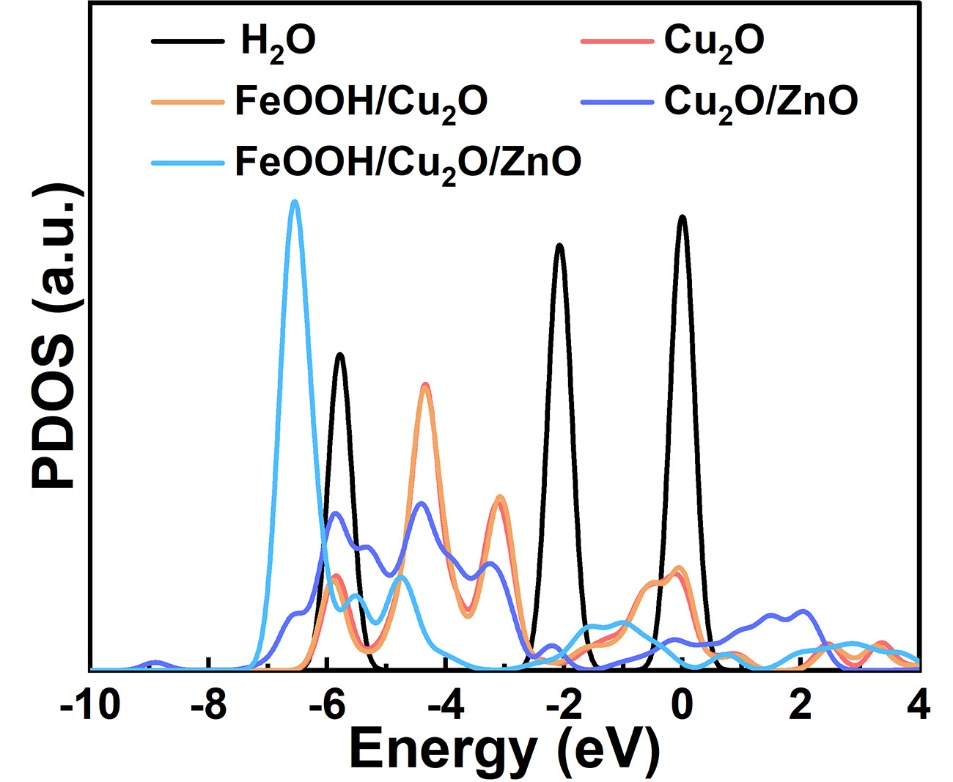


**Figure S42.** PDOS of H_2_O after adsorption of photocathodes.


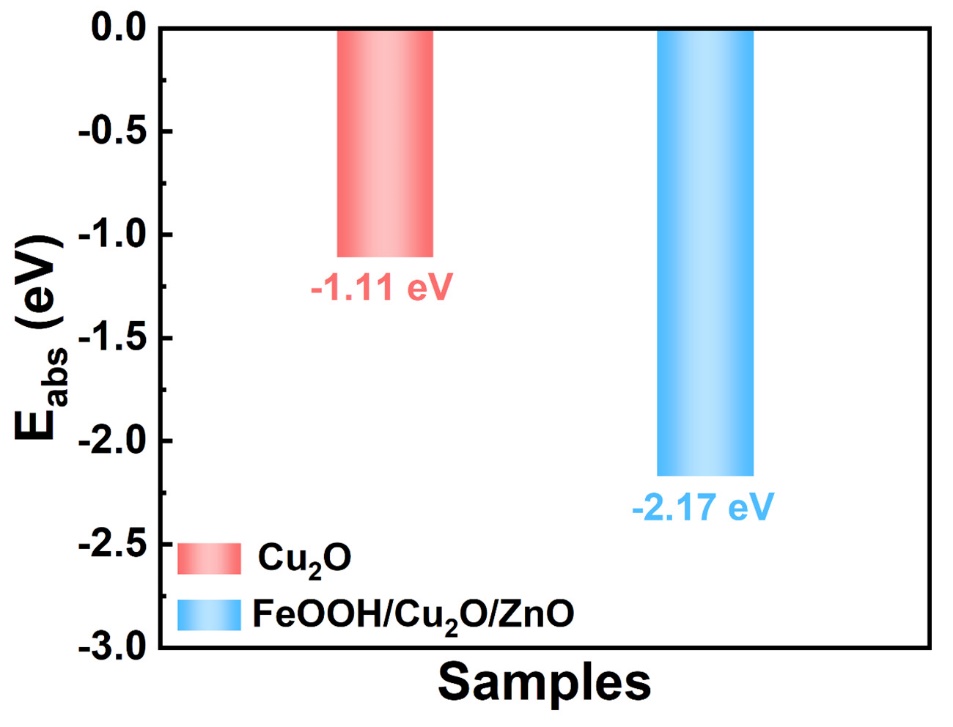


**Figure S43.** H_2_O energy diagram after adsorption of Cu_2_O and FeOOH/Cu_2_O/ZnO.


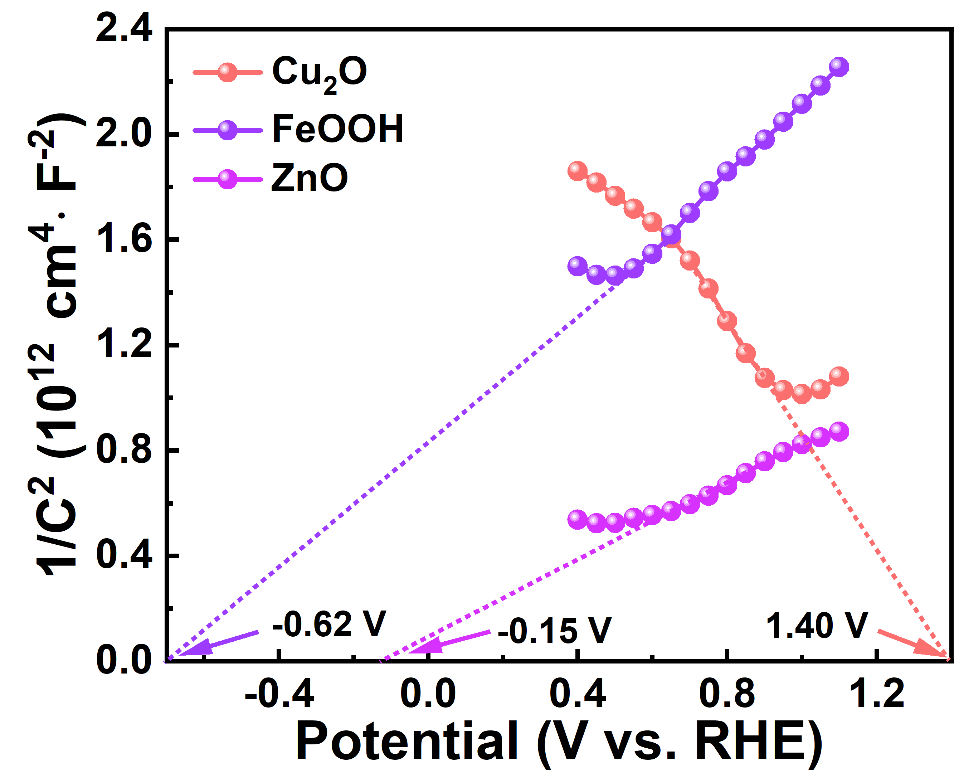


**Figure S44.** The M-S plots measured at 1 kHz under dark conditions.


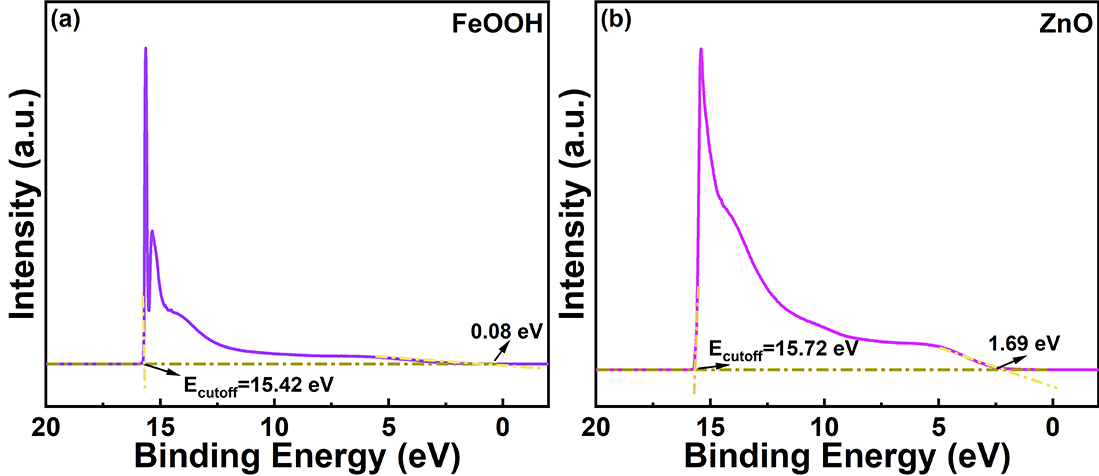


**Figure S45.** UPS spectra of FeOOH (a) and ZnO (b).


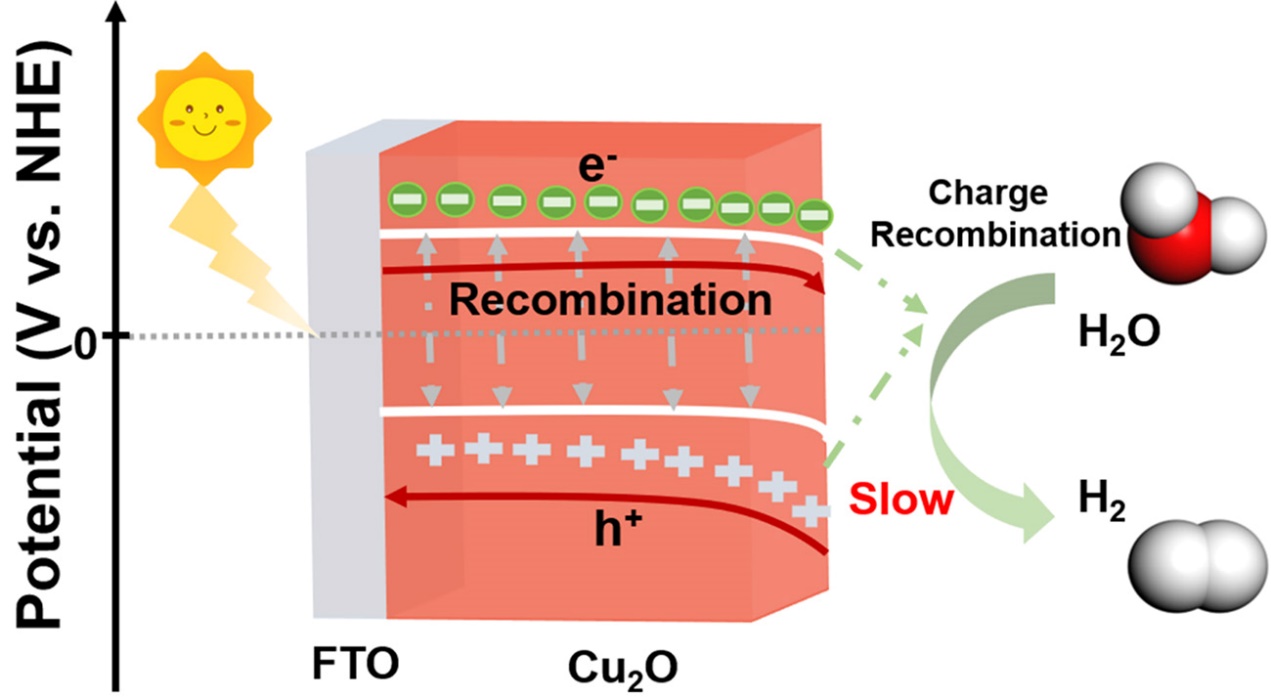


**Figure S46.** Schematic diagram of the photogenerated carrier transport behavior in the Cu_2_O photocathode under illumination.

**Table S1.** Comparison of PEC performance of the FeOOH/Cu_2_O/ZnO photocathodes with representative literature reports.^[1-10]^

(All the photocurrent is in unit of mA⋅cm^-2^ and all the potentials are referred to V vs. RHE)

| **Photocathodes** | **Electrolyte** | **Onset potential** | **Photocurrent density** | **Stability** | **Reference** |
| --- | --- | --- | --- | --- | --- |
| **FeOOH/Cu_2_O/ZnO** | **0.5 M**  **Na_2_SO_4_** | **+0.98** | **-5.4** | **10h** | **This work** |
| Cu_2_O/Ga_2_O_3_/ZnGeOx/TiO_2_ | 0.5M Na_2_SO_4_ and 0.1M sodium phosphate | +1.07 | -5.5 | 10 h | 1 |
| Cl-Cu_2_O/ZnO | 0.1 M KHCO_3_ | -0.2 | -4.7 | 5 h | 2 |
| FeOOH/Cu_2_O/PPy | 0.1 M Bu_4_NPF_6/_ACN | -1.5 | -3.5 | 2 h | 3 |
| Si/ZnO/Cu_2_O | 0.1 M KHCO_3_ | +0.2 | -5.5 | / | 4 |
| Cu_2_O/Al-doped ZnO/TiO_2_ | 0.5 m Na_2_SO_4_ solution buffered with 0.2 M potassium borate | +0.73 | -11.9 | 8 h | 5 |
| ME-Cu_2_O/ZnO/TiO_2_/Pt | 0.5 M Na_2_SO_4_ and 0.1 M KH_2_PO_4_ | +0.65 | -4.0 | 0.2 h | 6 |
| NaCo_2_O_4_/CuBi_2_O_4_ | 0.1 M  Na_2_S_2_O_8_ | +0.9 | -2.38 | / | 7 |
| Cu_2_O/WO_3_ | 0.5 M  Na_2_SO_4_ | +0.95 | -1.5 | 2.5 h | 8 |
| pn^+^-Si/W-TMOS/Pt | 1M  HClO_4_ | +0.55 | -32.5 | / | 9 |
| Cu_2_O/Ga_2_O_3_/TiO_2_/Sn-ED | 0.5 M  KHCO_3_ | +0.44 | -1.18 | 0.4 h | 10 |

**Table S2.** Related parameters derived from EIS Nyquist data by fitting with the equivalent circuit of samples.

| **Samples** | **Element** | **Parameter** | **Value** | **Significance** |
| --- | --- | --- | --- | --- |
| Cu_2_O | R_0_ | R | 1.77 KΩ | 0.981 |
|  | R_ct_ | R | 29.0 KΩ | 0.320 |
|  | CPE | Ceq | 67.4 µF | 0.564 |
|  | CPE | α | 515 m | 1.059 |
| FeOOH/Cu_2_O | R_0_ | R | 1.90 KΩ | 0.979 |
|  | R_ct_ | R | 18.7 KΩ | 0.325 |
|  | CPE | Ceq | 78.3 µF | 0.529 |
|  | CPE | α | 496 m | 0.935 |
| Cu_2_O/ZnO | R0 | R | 1.73 KΩ | 0.984 |
|  | R_ct_ | R | 16.0 KΩ | 0.287 |
|  | CPE | Ceq | 86.9 µF | 0.547 |
|  | CPE | α | 519 m | 0.957 |
| FeOOH/Cu_2_O/ZnO | R_0_ | R | 320 Ω | 0.979 |
|  | R_ct_ | R | 9.03 KΩ | 0.020 |
|  | CPE | Ceq | 36.9 µF | 0.523 |
|  | CPE | α | 684 m | 2.123 |

**Table S3.** Bode plot parameters of Cu_2_O

| **Bias** | **Element** | **Parameter** | **Value** | **Significance** |
| --- | --- | --- | --- | --- |
| 0.35 V | R_0_ | R | 2.24 KΩ | 0.991 |
|  | R_ct_ | R | 30.5 KΩ | 0.358 |
|  | CPE | Ceq | 40.6 µF | 0.558 |
|  | CPE | α | 604 m | 1.305 |
| 0.3 V | R_0_ | R | 1.76 KΩ | 0.979 |
|  | R_ct_ | R | 24.0 KΩ | 0.318 |
|  | CPE | Ceq | 68.0 µF | 0.564 |
|  | CPE | α | 512 m | 1.057 |
| 0.25 V | R_0_ | R | 1.82 KΩ | 0.981 |
|  | R_ct_ | R | 18.5 KΩ | 0.337 |
|  | CPE | Ceq | 65.4 µF | 0.558 |
|  | CPE | α | 520 m | 1.057 |
| 0.2 V | R_0_ | R | 1.83 KΩ | 0.982 |
|  | R_ct_ | R | 16.6 KΩ | 0.353 |
|  | CPE | Ceq | 68.0 µF | 0.544 |
|  | CPE | α | 520 m | 1.026 |
| 0.15 V | R_0_ | R | 1.86 KΩ | 0.981 |
|  | R_ct_ | R | 15.3 KΩ | 0.351 |
|  | CPE | Ceq | 73.1 µF | 0.531 |
|  | CPE | α | 512 m | 0.979 |

**Table S4.** Bode plot parameters of FeOOH/Cu_2_O/ZnO

| **Bias** | **Element** | **Parameter** | **Value** | **Significance** |
| --- | --- | --- | --- | --- |
| 0.35 V | R_0_ | R | 317 Ω | 0.981 |
|  | R_ct_ | R | 24.2 KΩ | 0.025 |
|  | CPE | Ceq | 35.5 µF | 0.524 |
|  | CPE | α | 695 m | 2.128 |
| 0.3 V | R_0_ | R | 316 Ω | 0.980 |
|  | R_ct_ | R | 19.2 KΩ | 0.046 |
|  | CPE | Ceq | 40.6 µF | 0.638 |
|  | CPE | α | 683 m | 2.099 |
| 0.25 V | R_0_ | R | 344 Ω | 0.983 |
|  | R_ct_ | R | 8.98 KΩ | 0.046 |
|  | CPE | Ceq | 43.3 µF | 0.696 |
|  | CPE | α | 685 m | 2.047 |
| 0.2 V | R_0_ | R | 375 Ω | 0.987 |
|  | R_ct_ | R | 5.43 KΩ | 0.086 |
|  | CPE | Ceq | 40.2 µF | 0.695 |
|  | CPE | α | 703 m | 2.040 |
| 0.15 V | R_0_ | R | 374 Ω | 0.987 |
|  | R_ct_ | R | 3.99 KΩ | 0.196 |
|  | CPE | Ceq | 40.4 µF | 0.691 |
|  | CPE | α | 702 m | 2.050 |

**Table S5.** The summary of fitted lifetime from the TRPL spectra.

| **samples** | **τ_1_ (ns)** | **τ_2_ (ns)** | **A_1_** | **A_2_** | **τ_ave_ (ns)** |
| --- | --- | --- | --- | --- | --- |
| Cu_2_O | 3.83 | 1.28 | 2.66 | 0.44 | 3.70 |
| FeOOH/Cu_2_O | 4.38 | 1.78 | 2.83 | 0.48 | 4.21 |
| Cu_2_O/ZnO | 5.23 | 2.24 | 2.26 | 0.51 | 4.97 |
| FeOOH/Cu_2_O/ZnO | 7.85 | 7.33 | 2.04 | 0.93 | 7.69 |

**References**

[1] J. Cheng, L. Wu, J. Luo, Improving the photovoltage of Cu_2_O photocathodes with dual buffer layers, *Nat. Commun*. **2023**, *14*, 7228.

[2] S.-T. Guo, Z.-Y. Tang, Y.-W. Du, T. Liu, T. Ouyang, Z.-Q. Liu, Chlorine anion stabilized Cu_2_O/ZnO photocathode for selective CO_2_ reduction to CH_4_, *Appl. Catal. B Environ*. **2023**, *321*, 122035.

[3] Y. Jia, Z. Tian, G. Jingyu, An effective integrated Cu_2_O photocathode to boost photoelectrocatalytic CO_2_ conversion, *J. Mater. Chem. A*. **2023**, *11*, 11411.

[4] M. Kan, C. Yang, Q. Wang, Q. Zhang, Y. Yan, K. Liu, A. Guan, G. Zheng, Defect‐assisted electron tunneling for photoelectrochemical CO_2_ reduction to ethanol at low overpotentials, *Adv. Energy Mater*. **2022**, *12*, 2201134.

[5] D. S. Kim, Y. B. Kim, J. H. Choi, H. W. Suh, H. H. Lee, K. W. Lee, S. H. Jung, J. J. Kim, N. G. Deshpande, H. K. Cho, Toward simultaneous achievement of outstanding durability and photoelectrochemical reaction in Cu_2_O photocathodes via electrochemically designed resistive switching, *Adv. Energy Mater*. **2021**, *11*, 2101905.

[6] C. Qin, X. Chen, N. Jiang, R. Liang, Z. Li, Z. Zheng, J. Wu, H. Chi, Z. Ye, L. Zhu, Surface densification strategy assisted efficient Cu_2_O heterojunction photocathode for solar water splitting, *Mater. Today Nano* **2023**, *21*, 100294.

[7] M. Sun, B. Liu, W. Han, Z. Zhang, M. Xie, CuBi_2_O_4_ photocathode with integrated electric field for enhanced H_2_O_2_ production, *Appl. Catal. B Environ*. **2022**, *304*, 120980.

[8] X. Wen, C. Fu, H. Zhan, J. Dai, R. Zhang, Y. Xia, H. Peng, H. Chu, F. Xu, L. Sun, WO_3_ coating enhances the performance of Cu_2_O photocathodes in solar water splitting cells, *ACS Appl. Nano Mater*. **2024**, *7*, 14936.

[9] B. Wu, T. Wang, B. Liu, H. Li, Y. Wang, S. Wang, L. Zhang, S. Jiang, C. Pei, J. Gong, Stable solar water splitting with wettable organic-layer-protected silicon photocathodes, *Nat. Commun*. **2022**, *13*, 4460.

[10] M. Xia, L. Pan, Y. Liu, J. Gao, J. Li, M. Mensi, K. Sivula, S. M. Zakeeruddin, D. Ren, M. Grätzel, Efficient Cu_2_O photocathodes for aqueous photoelectrochemical CO_2_ reduction to formate and syngas, *J. Am. Chem. Soc*. **2023**, *145*, 27939.
